# Dual tumour–myeloid targeting of glioblastoma with GPNMB CAR-T cells

**DOI:** 10.1038/s41586-026-10641-1

**Published:** 2026-07-01

**Authors:** Neil Savage, Shan Grewal, Muhammad Vaseem Shaikh, Franz J. Zemp, Dillon Mckenna, Nicholas Mikolajewicz, Hinda Najem, Joanna Pyczek, Jiuran Wei, Mohamed A. B. Taleb, Lucas C. Asselstine, Alisha Anand, Shawn C. Chafe, Kui Zhai, William T. Maich, Chirayu R. Chokshi, Hardikkumar Patel, Tiegan E. Korman, Minomi Subapanditha, Zoya Tabunshchyk, Nazanin Tatari, Petar Miletic, David Chen, Sebastian Pacheco, Abdelsimar T. Omar, Bill Wang, Hong Han, Jennifer A. Chan, Kevin R. Brown, Chitra Venugopal, Thomas Kislinger, Amy B. Heimberger, Jason Moffat, Douglas J. Mahoney, Sheila K. Singh

**Affiliations:** 1 Department of Biochemistry and Biomedical Sciences, McMaster University, Hamilton, Ontario Canada; 2 Centre for Discovery in Cancer Research, McMaster University, Hamilton, Ontario Canada; 3 Department of Surgery, Faculty of Health Sciences, McMaster University, Hamilton, Ontario Canada; 4 Arnie Charbonneau Cancer Institute, Cumming School of Medicine, University of Calgary, Calgary, Alberta Canada; 5 Department of Biochemistry and Molecular Biology, University of Calgary, Calgary, Alberta Canada; 6 Department of Molecular Genetics, University of Toronto, Toronto, Ontario Canada; 7 Program in Genetics and Genome Biology, The Hospital for Sick Children, Toronto, Ontario Canada; 8 Feinberg School of Medicine, Northwestern University, Chicago, IL USA; 9 McMaster Immunology Research Centre, McMaster University, Hamilton, Ontario Canada; 10 Division of Neurosurgery, Department of Surgery, Faculty of Health Sciences, McMaster University, Hamilton, Ontario Canada; 11 Department of Medical Biophysics, University of Toronto, Toronto, Ontario Canada; 12 Department of Microbiology, Immunology and Infectious Disease, University of Calgary, Calgary, Alberta Canada; 13 School of Cancer and Pharmaceutical Sciences, Comprehensive Cancer Centre, King’s College London, London, UK

**Keywords:** Cancer immunotherapy, Target validation, CNS cancer

## Abstract

Glioblastoma is a lethal brain tumour for which current multimodal treatment rarely prevents recurrence^1^. Therapeutic failure is driven by extensive intratumoural cellular heterogeneity^2^ with a microenvironment dominated by tumour-associated macrophages that sustain tumour growth and immunosuppression^3^. Although chimeric antigen receptor (CAR)-T cell therapies are being developed for glioblastoma, sustained response has been undermined by non-uniform antigen expression, antigen loss and microenvironmental barriers that are not directly engaged by tumour-targeting designs^4^. These limitations motivate new strategies that address the disease as a coupled tumour–immune system rather than a single malignant compartment. Here we use a multi-omic target discovery platform to identify GPNMB as a dual-compartment antigen in glioblastoma. Anti-GPNMB CAR-T cells showed potent anti-tumour activity, with long-term disease control in orthotopic patient-derived xenografts and syngeneic glioma models through concomitant depletion of GPNMB^+^ tumour and immunosuppressive myeloid populations. By collapsing tumour control and microenvironmental reprogramming, these findings provide a new strategy for antigen selection and targeting in heterogenous, myeloid-rich solid cancers.

## Abstract (web summary)

Integrated multi-omic profiling of glioblastoma reveals GPNMB as a shared antigen in tumour cells and the surrounding microenvironment, and GPNMB-targeted chimeric antigen receptor T cells demonstrate therapeutic activity in vitro and in animal models.

## Main

Glioblastoma (GBM) is the most aggressive primary brain tumour in adults and remains uniformly fatal. An aggressive standard of care—surgery, radiation and chemotherapy—provides only transient benefit, with a median overall survival of less than 15 months and near-universal recurrence^1,5^. A central challenge of GBM is its profound spatial and temporal heterogeneity, which enables rapid adaptation under therapeutic pressure and limits durability of targeted approaches^2^. In parallel, an immunosuppressive tumour microenvironment (TME), dominated by tumour-associated macrophages (TAMs), further constrains anti-tumour immunity^3^. A substantial body of work supports hierarchical organization within GBM in which glioma stem cells (GSCs)—tumour-propagating cells with self-renewal capacity and multilineage differentiation potential—contribute disproportionately to therapy resistance and relapse^6^. These cells can cycle between phenotypic states and occupy specialized niches, enabling them to survive stress and reconstitute heterogenous tumour populations after treatment^7^. As a result, effective therapies probably need to pursue targets that are present not only on bulk tumour cells but also on GSCs.

Adoptive cell therapies, including chimeric antigen receptor (CAR)-T cells, offer a compelling strategy to eliminate tumour cells, but durable responses in GBM have been limited by target non-uniformity, antigen loss and local immune suppression^4^. These challenges motivate efforts to identify novel antigens that are cell surface-accessible, broadly represented across the tumour, and functionally linked to stromal or immune compartments that sustain disease. Here we apply an integrated target discovery approach using multi-omic profiling of tumours from patients to nominate new therapeutically targetable surface targets in GBM. We identified GPNMB as a candidate with multi-compartment expression in GBM. We developed anti-GPNMB CAR-T cells and evaluated their activity across complementary preclinical models to show that targeting a shared antigen across tumour and myeloid cells can overcome key barriers to durable immunotherapeutic efficacy in GBM.

## GPNMB is a therapeutic target in GBM

**Fig. 1:**
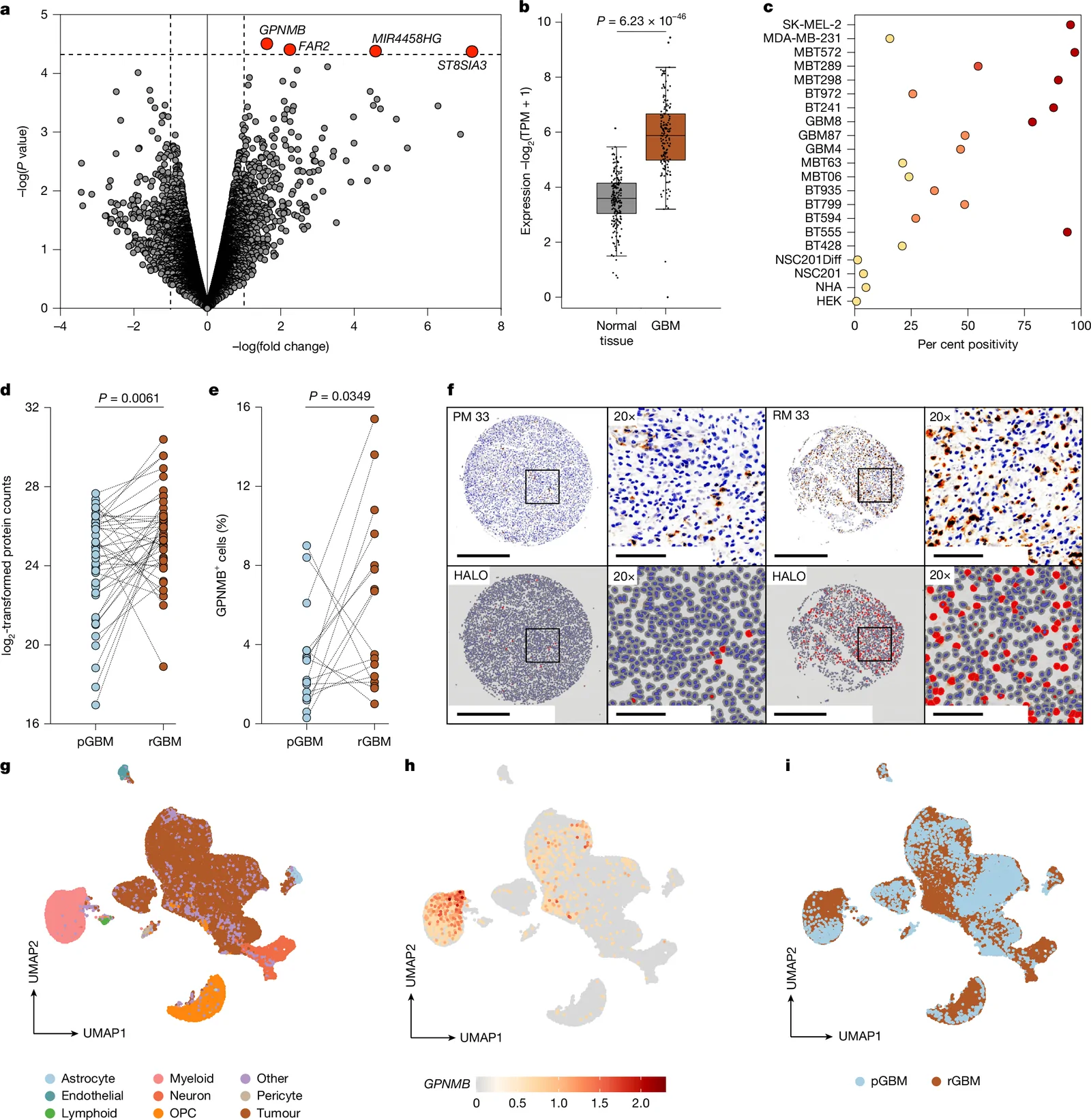
Identification of GPNMB as a clinically relevant target in GBM. **a**, RNA-seq differential gene expression analysis comparing CD133^+^ and CD133^−^ GSC fractions. GPNMB is highlighted among the most significantly enriched transcripts in the CD133^−^ populations. *P* value calculated by quasi-likelihood *F*-test. **b**, *GPNMB* transcript abundance in GBM tumours from TCGA (*n* = 160) versus normal brain tissues from GTEx (*n* = 207). Data presented as mean ± s.e.m. In box plots, the centre line shows the median, box limits delineate the interquartile range and whiskers extend to the range of values. *P* value calculated by unpaired two-sided Student’s *t*-test. TPM, transcripts per million. **c**, Flow cytometry quantification of surface GPNMB across 15 patient-derived GSC lines, non-GBM cancer cell lines (melanoma, SK-MEL-2; triple-negative breast cancer, MDA-MB-231) and non-malignant control cell types (HEK293T (HEK), normal human astrocytes (NHA), fetal NSCs (NSC201) and differentiated NSC201 (NSC201Diff)). Per cent positivity defined relative to fluorophore-minus-one control. **d**, Matched primary GBM (pGBM) and recurrent GBM (rGBM) proteomics demonstrating increased GPNMB protein abundance at recurrence (paired samples connected; *n* = 43; *P* value from unpaired two-sided Student’s *t*-test). **e**, Quantification of GPNMB^+^ cells from matched primary and recurrent GBM IHC using HALO image analysis (paired tumours connected; *n* = 16; *P* value from unpaired two-sided Student’s *t*-test). **f**, Representative IHC images from a matched primary (PM33) and recurrent (RM33) tumour show increased GPNMB staining at recurrence, with corresponding HALO detection overlays (red). Scale bars, 500 µm (whole-section views) and 100 µm (enlarged views). **g**–**i**, UMAP of scRNA-seq from primary (*n* = 10 samples) and recurrent (*n* = 8 sample) GBM specimens annotated by major cell populations (**g**), *GPNMB* expression (**h**) and primary or recurrent status (**i**). Source data

CD133 is an established surface marker that is used to identify both healthy neural stem cells (NSCs)^8^ and GSCs^9^, including chemoresistant populations^10^. Residual and recurrent disease, however, often reflects heterogenous malignant states that are not fully captured by CD133 expression alone^11^. We therefore performed RNA sequencing (RNA-seq) on flow-sorted CD133^+^ and CD133^–^ fractions isolated from four primary patient-derived GSC lines to nominate targets that may persist alongside CD133^+^ cells. Multidimensional scaling analysis comparing CD133^+^ and CD133^–^ GSC populations identified four genes that were significantly upregulated in CD133^–^ GSCs (Fig. 1a). Among these, *GPNMB* emerged as the most significantly upregulated gene. *GPNMB* encodes a glycosylated type I transmembrane protein that is overexpressed in several cancers, including GBM, melanoma, osteosarcoma and triple-negative breast cancer^12^.

**Extended Data Fig. 1:**
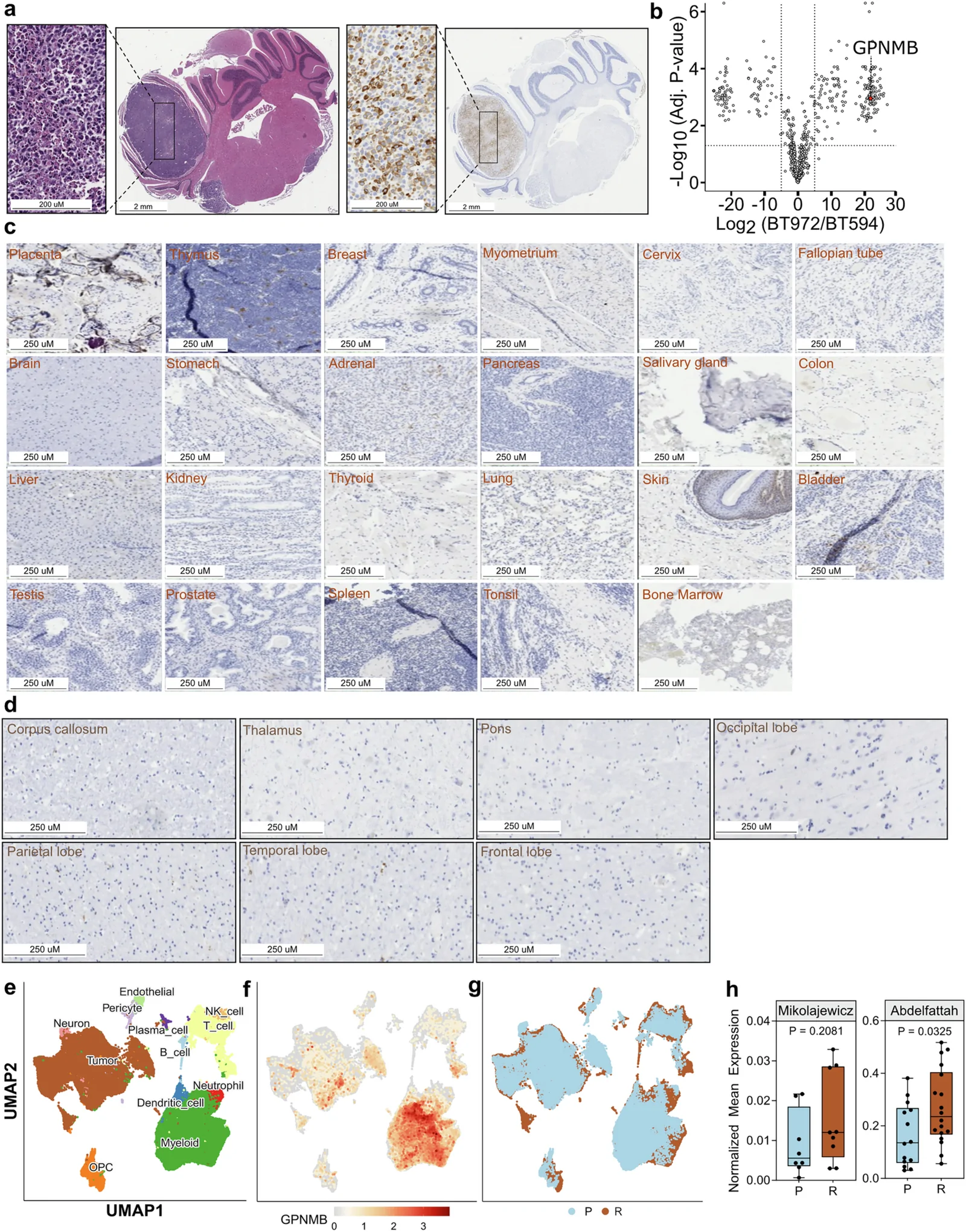
GPNMB protein is enriched in glioblastoma and shows minimal staining across normal tissues. (**a**) H&E and GPNMB IHC of an orthotopic BT972 patient-derived xenograft brain section showing GPNMB staining localized to tumor regions (whole-section views with corresponding magnified insets). Scale bars, 2 mm (whole section) and 200 µm (insets). Representative image from n = 3 mice. (**b**) Proteomic differential expression between BT972 and matched primary GSC line BT594 (log_2_[BT972/BT594] versus −log_10_[adjusted *P* value]). *P* value determined by Wilcoxon rank-sum test. (**c**) GPNMB IHC cores from normal human tissue microarray spanning major organ systems (tissue labels as shown). Scale bars, 250 µm. (**d**) GPNMB IHC across normal adult brain regions (white matter and multiple cortical/subcortical regions as labeled). Scale bars, 250 µm. (**e**) UMAP of single-cell RNA-seq (scRNA-seq) dataset of primary (n = 14) and recurrent (n-18) GBM from Abdelfattah et al.^18^. (**f-g**) GPNMB expression (f) and primary/recurrent status (g) projected onto the UMAP. (**i**) The same UMAP colored by primary (P) or recurrent (R) tumor class. (**h**) GPNMB expression in primary versus recurrent GBM samples across independent scRNA-seq datasets. Boxplots show distribution of per-patient mean normalized GPNMB expression in primary (P; blue) and recurrent (R; brown) tumors for Mikolajewicz et al.^17^ (n = 18) and Abdelfattah et al.^18^ (n = 32) datasets; each dot represents one non-zero GPNMB expressing sample. Centre line, median; box limits, interquartile range; whiskers, minimum and maximum values. *P* values from two-sided unpaired Student’s *t*-test. Source data

To establish clinical utility, we evaluated GPNMB expression in orthogonal human GBM datasets and patient samples. In bulk transcriptomic data, *GPNMB* expression was significantly higher in GBM (The Cancer Genome Atlas (TCGA)) than in normal brain tissue from the Genotype-Tissue Expression (GTEx) project (Fig. 1b). At the protein level, flow cytometry profiling of surface GPNMB across 15 patient-derived GSC samples demonstrated increased expression relative to normal cell controls (HEK293T, NSCs, astrocytes and differentiated NSCs) in which GPNMB was nearly absent (Fig. 1c). Benchmarking against previous indications of anti-GPNMB therapy showed high expression in melanoma (SK-MEL02; 95% GPNMB^+^) and modest expression in breast cancer (MDA-MB-231; 15%) (Fig. 1c). Consistent with these findings, GPNMB was detected by immunohistochemistry (IHC) in our GSC patient-derived xenografts (PDXs) (Extended Data Fig. 1a).

Relapsed GBM tumours are molecularly and phenotypically distinct from their newly diagnosed counterparts^13^, prompting us to explore whether GPNMB expression changes after treatment. In our previously published whole-cell proteomic profiling of matched primary and recurrent GBM samples^14^, GPNMB was significantly increased in recurrent GBM compared with primary specimen (Fig. 1d). GPNMB was detected in all 86 tumours profiled and ranked among the top percentile of proteins upregulated at relapse. We corroborated this by IHC in an independent subset of patient-matched primary and recurrent GBM tissues, observing higher GPNMB staining in recurrent tumours (Fig. 1e,f). Whole-cell proteomics performed on patient-matched GSC lines demonstrated higher GPNMB abundance in the recurrent line compared with its matched primary-derived counterpart (Extended Data Fig. 1b).

To gauge potential on-target, off-tumour risk, we stained a normal human tissue microarray (*n* = 23) using IHC (Extended Data Fig. 1c). Low-level GPNMB expression was observed in previously reported cell types, such as bone^15^ and skin^16^, or where highly dense immune populations exist, such as the thymus. Notably, for intracranially delivered therapies, GPNMB was not detected across multiple regions of healthy adult brain (Extended Data Fig. 1d), supporting a therapeutic window for targeting GBM while minimizing risk to normal central nervous system (CNS) tissue.

To further characterize the distribution of GPNMB expression within patient tumour specimens, we performed single-cell RNA sequencing (scRNA-seq) on 18 GBM samples (8 primary GBM, 10 recurrent GBM; 14 out of 18 matched tissue)^17^. Cell-type annotation resolved malignant cells, immune populations and non-malignant brain cells (Fig. 1g). Across patients, *GPNMB* transcripts localized predominantly to malignant and myeloid cells (Fig. 1h), and expression was higher in recurrent tumours (Fig. 1i). These expression patterns were corroborated across other publicly available scRNA-seq datasets^18^ (Extended Data Fig. 1e-h).

Collectively, these data establish GPNMB as a tumour-associated cell surface antigen in GBM that is present on malignant cells and the myeloid compartment, but largely absent from healthy adult brain. This supports its prioritization for therapeutic targeting to engage both GBM cells and the TME.

## GPNMB drives GBM growth

**Fig. 2:**
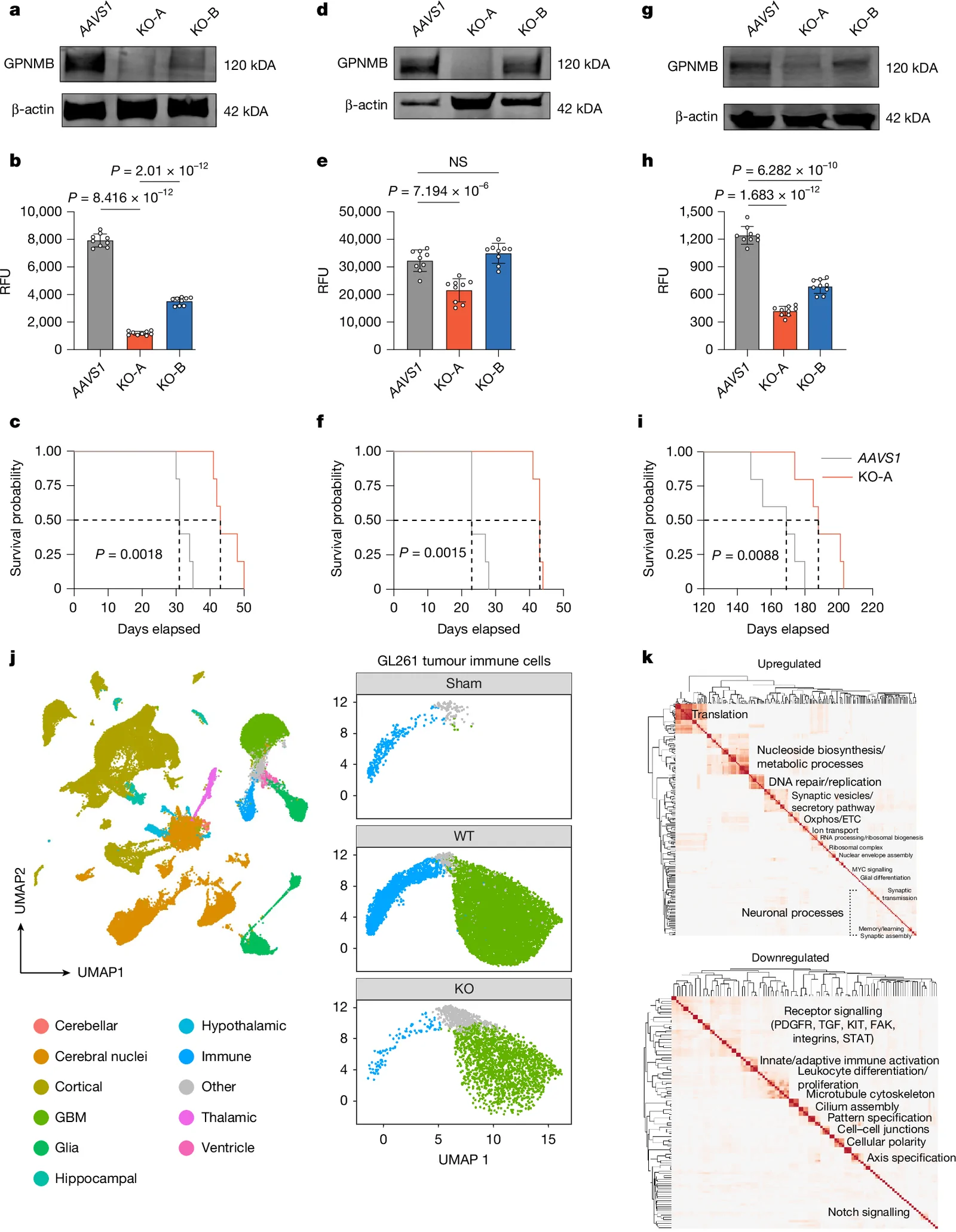
GPNMB functionally supports GBM progression and reprogrammes malignant states in vivo. **a**,**d**,**g**, Immunoblot validation of CRISPR–Cas9-mediated *GPNMB* knockout in GBM8 (**a**), GBM4 (**d**) and MBT06 (**g**) GSCs using two independent synthetic guide RNAs (KO-A and KO-B), with *AAVS1*-targeted cells as editing controls. **b**,**e**,**h**, Cell proliferation measured as relative fluorescence units (RFU) following GPNMB knockout in GBM8 (**b**), GBM4 (**e**) and MBT06 (**h**). Data are mean ± s.d. *n* = 9 experimental replicates; unpaired two-sided Student’s *t*-test. **c**,**f**,**i**, Kaplan–Meier survival analysis of NSG mice bearing orthotopic *GPNMB*-knockout or * AAVS1*-targeted GBM8 (**c**), GBM4 (**f**) or MBT06 (**i**) tumours (*n* = 5 mice per group). *P* values by log-rank Mantel–Cox test. **j**, UMAP embedding of scRNA-seq data from intracranial GL261 tumours and sham brains, annotated by major cell populations, with stratified views of GBM and immune cells recovered from each group. KO, *Gpnmb* knockout; WT, wild type. **k**, GSEA summarizing pathways that are differentially regulated in wild-type versus *Gpnmb*-knockout GL261 malignant cells, with representative enriched gene sets grouped as upregulated and downregulated programmes (pathway labels shown). ETC, electron transport chain; oxphos, oxidative phosphorylation. NS, not significant (*P* ≥ 0.05). Source data

To investigate the functional contribution of GPNMB to tumorigenesis, we used CRISPR–Cas9 to knock out *GPNMB* in patient-derived GSCs (Fig. 2a). *GPNMB* knockout led to a marked decrease in cell proliferation in vitro (Fig. 2b). To assess the effect of GPNMB on GBM tumorigenicity in vivo, *GPNMB*-knockout GSCs were orthotopically implanted into NSG mice. Mice carrying *GPNMB*-knockout tumours exhibited significantly prolonged survival compared with those with *GPNMB*-expressing tumours (transduced with * AAVS1*-targeting single guide RNA (sgRNA)) (Fig. 2c). These findings were consistent across multiple GSC samples (Fig. 2d–i), demonstrating that GPNMB contributes to tumour proliferation and growth.

## GPNMB supports MES states and immune recruitment

**Extended Data Fig. 2:**
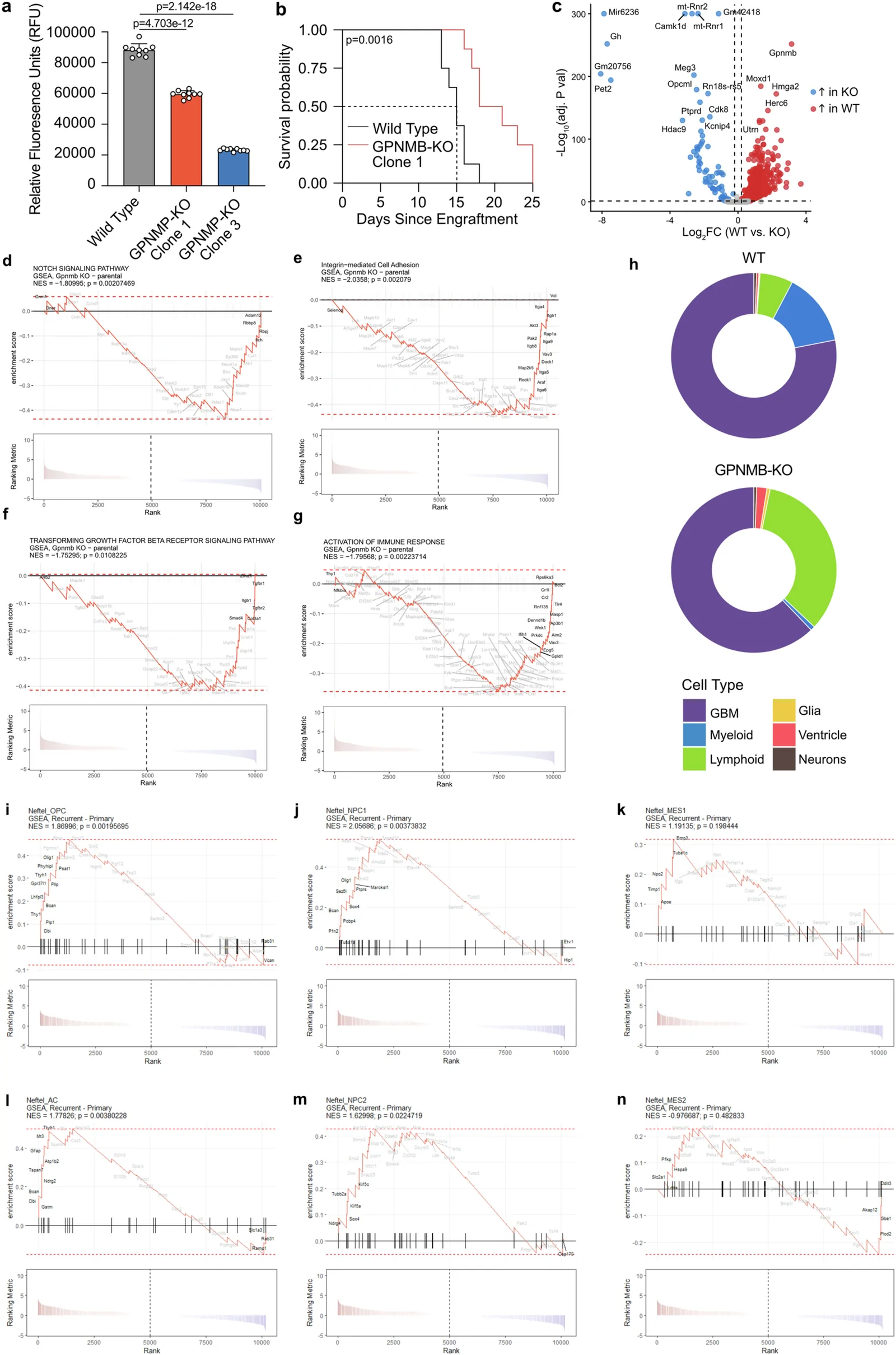
GPNMB loss suppresses GL261 growth and shifts malignant programs towards developmental/astrocytic states. (**a**) GL261 proliferation measured as relative fluorescence units (RFU) for wild-type (WT) versus two independent GPNMB-knockout (KO) clones (mean ± s.d., n = 9 experimental replicates; unpaired two-sided Student’s t-test). (**b**) Kaplan-Meier survival of C57BL/6 mice bearing orthotopic WT versus GPNMB^KO^ GL261 tumors (n = 8; log-rank Mantel-Cox test). (**c**) Volcano plot showing differential expression between GL261 WT and GPNMB^KO^ tumor cell. Significant DEGs (|log_2_FC| > 0.2, adjusted *P* value < 0.05 based on two-sided Wilcoxon rank-sum test) colored as indicated. (**d–g**) Gene set enrichment analysis (GSEA) comparing WT versus GPNMB^KO^ GL261 malignant cells for selected pathways (gene sets labeled; statistics shown in panels). (**h**) Cell-type composition of WT versus GPNMB^KO^ GL261 tumors quantified from scRNA-seq (pie charts; proportions as shown). (**i–n**) GSEA using Neftel malignant cell-state signatures^20^ comparing WT versus GPNMB^KO^ GL261 malignant cells, showing enrichment of OPC/NPC and astrocyte-like programs (i,j,l,m) and no enrichment of mesenchymal programs (k,n) (signatures labeled; statistics shown in panels). Gene rankings generated using Seurat *FindMarkers* with a two-sided Wilcoxon rank-sum test, and reported *P* values correspond to enrichment *P* values calculated within the GSEA framework. Source data

To complement PDX models that incompletely capture tumour–immune interactions, we turned to the syngeneic GL261 mouse glioma model to study GPNMB function within an intact TME. *Gpnmb*-knockout GL261 clones were generated using CRISPR–Cas9 and characterized. Consistent with our observations in human GSCs, *Gpnmb* knockout reduced glioma cell proliferation in vitro (Extended Data Fig. 2a). In vivo, intracranial implantation of *Gpnmb*-knockout GL261 cells in C57BL/6 mice conferred a significant survival advantage over wild-type cells (Extended Data Fig. 2b).

At end-point, tumour-bearing hemispheres were sectioned for scRNA-seq analysis (two sham, three bearing wild-type tumours and two bearing *Gpnmb*-knockout tumours). Uniform manifold approximation and projection (UMAP) analysis resolved major brain and tumour-associated populations (Fig. 2j). Cell types were annotated following the framework established by Zeisel et al.^19^. Malignant cells formed a dense cluster marked by canonical tumour markers such as *Sox6* and *Hmga2*. Consistent with the genetic perturbation, *Gpnmb* transcripts were reduced in knockout tumours, along with 975 other significant differentially expressed genes (DEGs) within the tumour compartment (Extended Data Fig. 2c). Gene set enrichment analysis (GSEA) highlighted activation of metabolic and growth-associated programmes, including increased oxidative phosphorylation and MYC signalling in *Gpnmb*-knockout tumours (Fig. 2k). Downregulated pathways reveal a decrease in Notch and TGFβ signalling and integrin-mediated adhesion pathways (Extended Data Fig. 2d–f). Gene sets linked to innate and adaptive immune activation, and leukocyte differentiation were also downregulated (Fig. 2k and Extended Data Fig. 2g), consistent with altered immune cell engagement following loss of *Gpnmb*.

Additionally, GPNMB status was associated with differences in both tumour composition and cell state^20^: *Gpnmb*-knockout tumours showed proportionally less myeloid recruitment and more lymphoid infiltration than wild-type tumours (Fig. 2j and Extended Data Fig. 2h). *Gpnmb*-knockout GL261 cells preferentially adopted developmental-like transcriptional states characterized by enrichment of developmental-like (oligodendrocyte progenitor (OPC)-like, neural progenitor cell 1 (NPC1)-like and NPC2-like) and astrocyte-like, but not mesenchymal (MES)-like programmes, compared with wild-type tumours (Extended Data Fig. 2i–n). Together, these findings indicate that *Gpnmb* perturbation in gliomas is accompanied by broad transcriptional reprogramming and TME alterations with less immune activation, consistent with GPNMB contributing to tumour–myeloid interactions.

## GPNMB CAR-T cells show potent anti-GBM activity

**Fig. 3:**
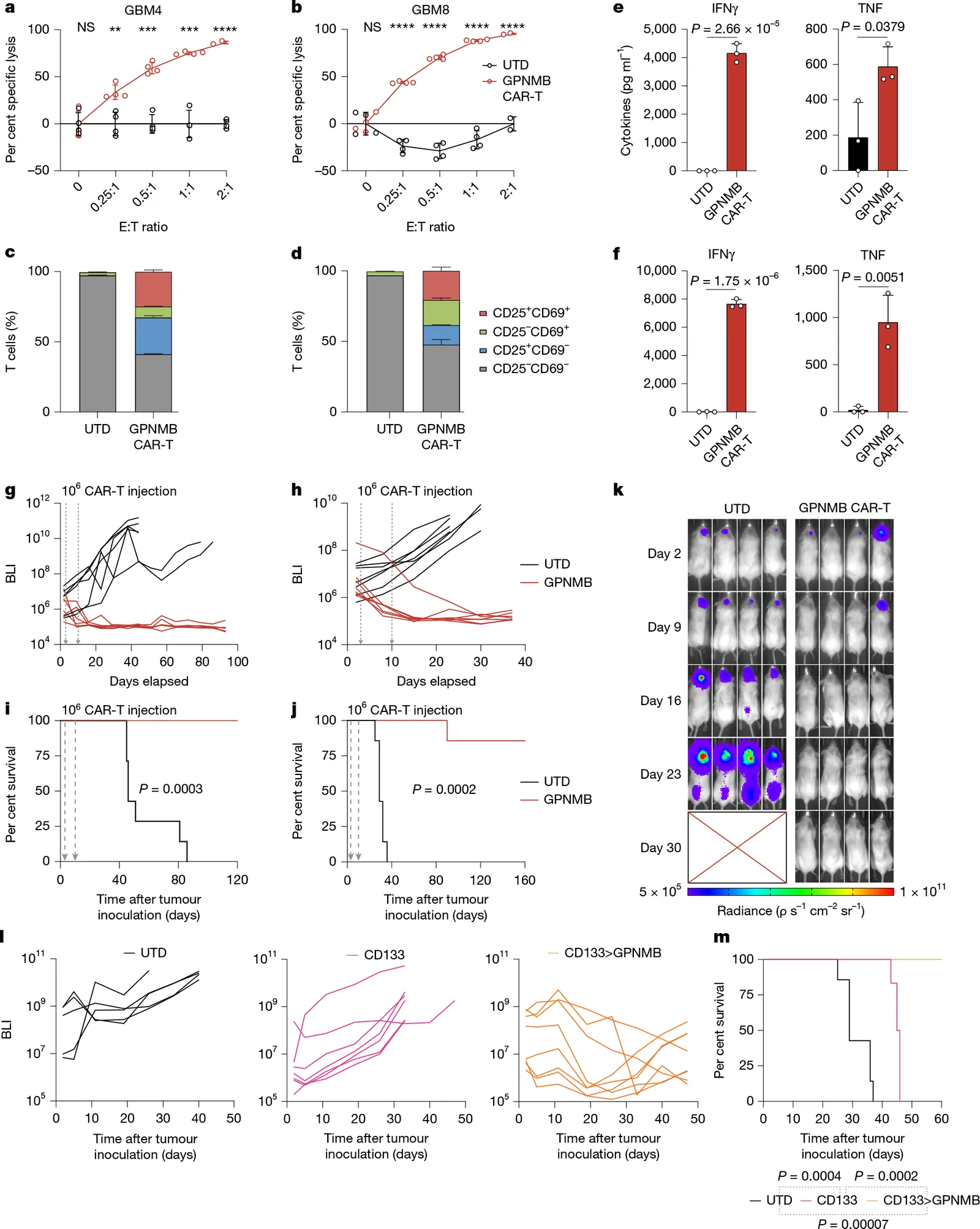
GPNMB CAR-T cells mount potent and curative anti-tumour response against GBM. **a**,**b**, Per cent specific lysis of ffluc-expressing GBM4 (**a**) and GBM8 (**b**) target cells co-cultured with UTD or anti-GPNMB CAR-T cells at various effector:target (E:T) ratios for 48 h. Data normalized against BLI of viable tumour cells when cultured in the absence of effector cells. Data are mean ± s.d.; *n*  =  4 experimental replicates; unpaired two-sided Student’s *t*-test. **c**,**d**, Flow cytometry assessment of T cell activation markers (CD25 and CD69) after 24 h co-culture with GBM4 (**c**) or GBM8 (**d**) cells at a 1:1 E:T ratio. Data are mean ± s.d.; *n*  =  4 experimental replicates. **e**,**f**, Quantification of IFNγ and TNF secretion in GBM4 (**e**) and GBM8 (**f**) cells co-cultured with UTD or GPNMB CAR-T cells at a 1:1 E:T ratio for 24 h, as determined using enzyme-linked immunosorbent assay (ELISA). Data are mean ± s.d.; *n*  =  3 experimental replicates; unpaired two-sided Student’s *t*-test. **g**–**j**, Orthotopic GBM PDX CAR-T trials: longitudinal tumour BLI and Kaplan–Meier survival following intracranial treatment with UTD or GPNMB CAR-T cells at indicated time points in mice engrafted with GBM4-ffluc (**g**,**i**; *n* = 6 mice per cohort) or GBM8-ffluc (**h**,**j**; *n* = 7 mice per cohort). *P* values from log-rank Mantel–Cox test. **k**, Representative BLI images of GBM8 trial from **h**. **l**,**m**, Sequential therapy in GBM8-ffluc PDXs: BLI (**l**) and survival (**m**) following intracranial CD133 CAR-T cell dosing (3 and 10 days post-inoculation) alone, or followed by GPNMB CAR-T cell dosing (CD133 > GPNMB; 12 and 18 days post-inoculation), compared with time-matched UTD controls (UTD: *n* = 5 mice; CD133: *n* = 7 mice; CD133>GPNMB: *n* = 8 mice). *P* values from log-rank (Mantel–Cox) test. *P < 0.05, **P < 0.01, ***P < 0.001, ****P < 0.0001. Source data

**Extended Data Fig. 3:**
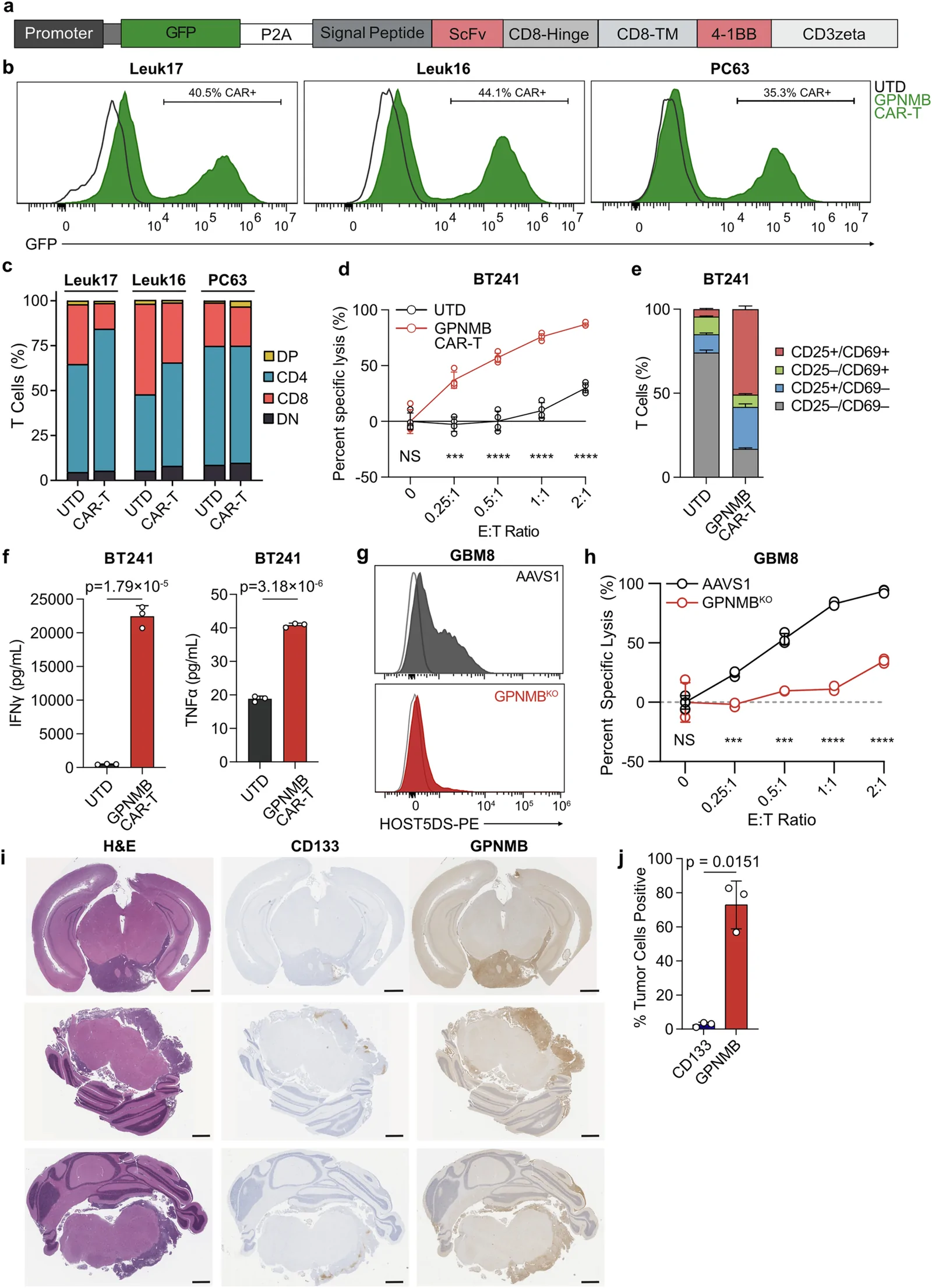
Generation of anti-GPNMB CAR-T Cells with antigen-dependent cytotoxicity against GBM cells. (**a**) Schematic of anti-GPNMB CAR cassette (GFP reporter, signal peptide, glembatumumab vedotin scFv, CD8 hinge/transmembrane, 4-1BB costimulatory and CD3ζ signaling domain). Created in BioRender; Venugopal, C. https://BioRender.com/dfzup0y (2026). (**b**) Flow cytometry of CAR transduction efficiency (GFP^+^) in T cells generated from three PBMC donors (Leuk17, Leuk16, PC63), with percentage of CAR^+^ cells indicated. (**c**) CD4/CD8 composition of untransduced (UTD) and CAR-transduced products from each donor (double-negative [DN], CD4^+^, CD8^+^, double-positive [DP] fractions). (**d-f**) Functional validation of Leuk17-derived CAR-T cells against BT241-ffluc targets: percent specific lysis across effector:target (E:T) ratios (d; mean ± s.d., n = 4 experimental replicates), T cell activation by CD25/CD69 expression (e; mean proportion ± s.d., n = 3 experimental replicates), and IFNγ and TNFα secretion measured by ELISA (f; mean ± s.d., n = 3 experimental replicates) after 24 h co-culture at 1:1 E:T. (**g**) Flow cytometry validation of CRISPR/Cas9-mediated GPNMB knockout in GBM8-ffluc targets relative to AAVS1-targetting controls. (**h**) Cytotoxicity assay comparing GPNMB CAR-T cell killing of AAVS1 versus GPNMB-knockout GBM8-ffluc targets across E:T ratios, demonstrating attenuated lysis in the absence of GPNMB (bars denote s.d., n = 4 experimental replicates). (**i**) Representative immunohistochemistry (IHC) for CD133 and GPNMB on brains harvested after intracranial CD133 CAR-T treatment, showing residual tumor regions with reduced CD133 and diffuse GPNMB staining; one representative section from each of three treated mice is shown (scale bars = 2 mm). (**j**) Quantification of percent CD133 and GPNMB positive cells across all tumor-burdened regions (defined on matched H&E) from CD133 CAR-T treated brains (mean ± s.d., n = 3 biological replicates). All *P* values from unpaired two-sided Student’s *t*-test (**P  <  0.01, ***P  <  0.001 and ****P  <  0.0001 or as specified). Source data

**Extended Data Fig. 4:**
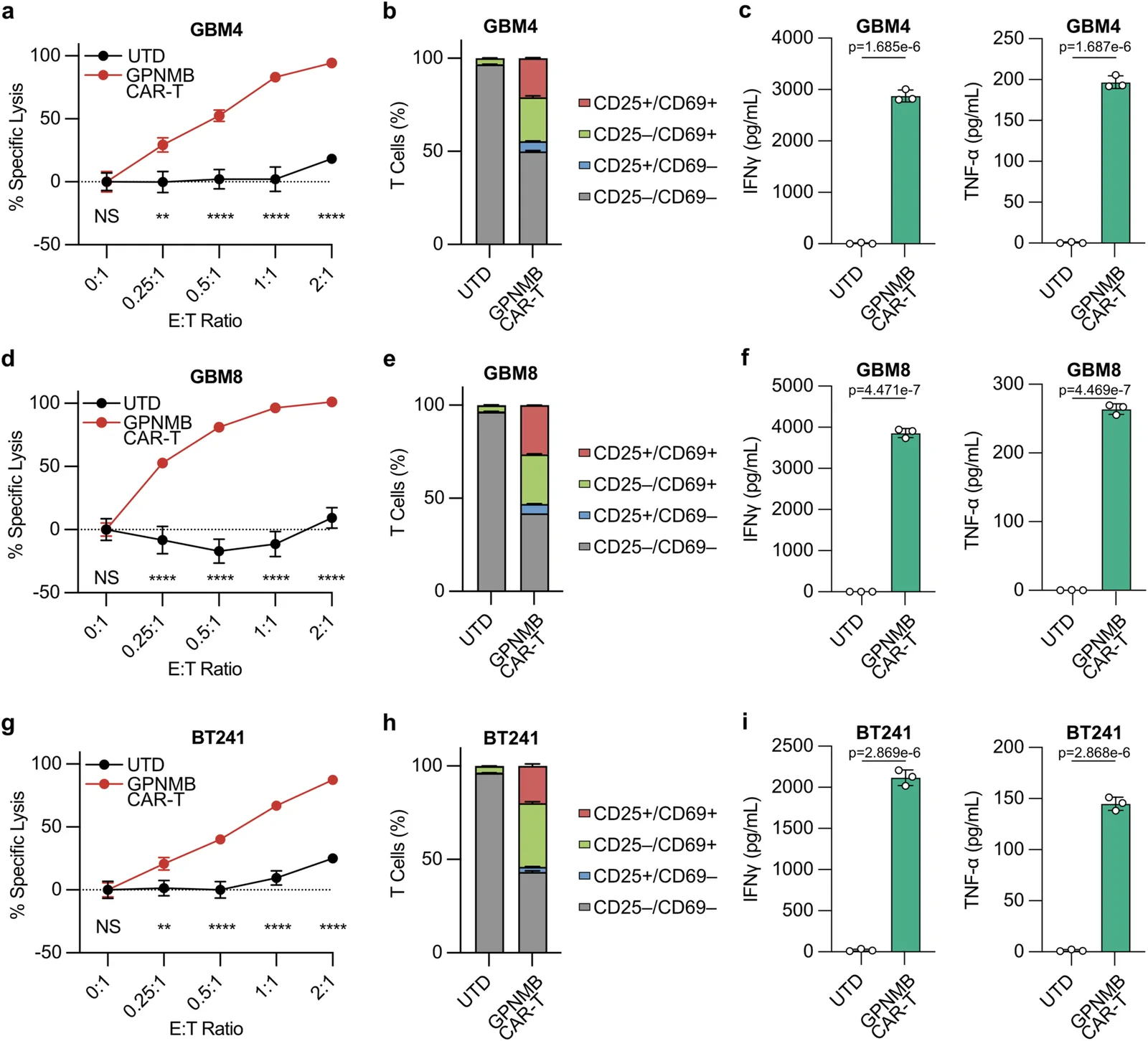
Leuk16-derived anti-GPNMB CAR-T cells demonstrate equivalent activity. (**a,d,g**) Percent specific lysis of ffluc-expressing GBM4 (a), GBM8 (d), and BT241 (g) targets after co-culture with Leuk16-derived untransduced (UTD) or anti-GPNMB CAR-T cells across effector:target (E:T) ratios after 48 h (mean ± s.d.; n = 4 experimental replicates). (**b,e,h**) Flow cytometry of T cell activation (CD25/CD69) following co-culture with GBM4 (b), GBM8 (e), or BT241 (h) at 1:1 E:T for 24 h, reporting the fraction of T cells in CD25/CD69 single or dual activation quadrants for UTD versus GPNMB CAR-T. (mean ± s.d.; n = 3 experimental replicates). (**c,f,i**) IFNγ and TNFα secretion measured by ELISA following co-culture of T cells with GBM4 (c), GBM8 (f), or BT241 (i) at 1:1 E:T for 24 h (mean ± s.d.; n = 3 experimental replicates). All *P* values from unpaired two-sided Student’s *t*-test (**P  <  0.01, ***P  <  0.001, ****P  <  0.0001, or as specified). Source data

**Extended Data Fig. 5:**
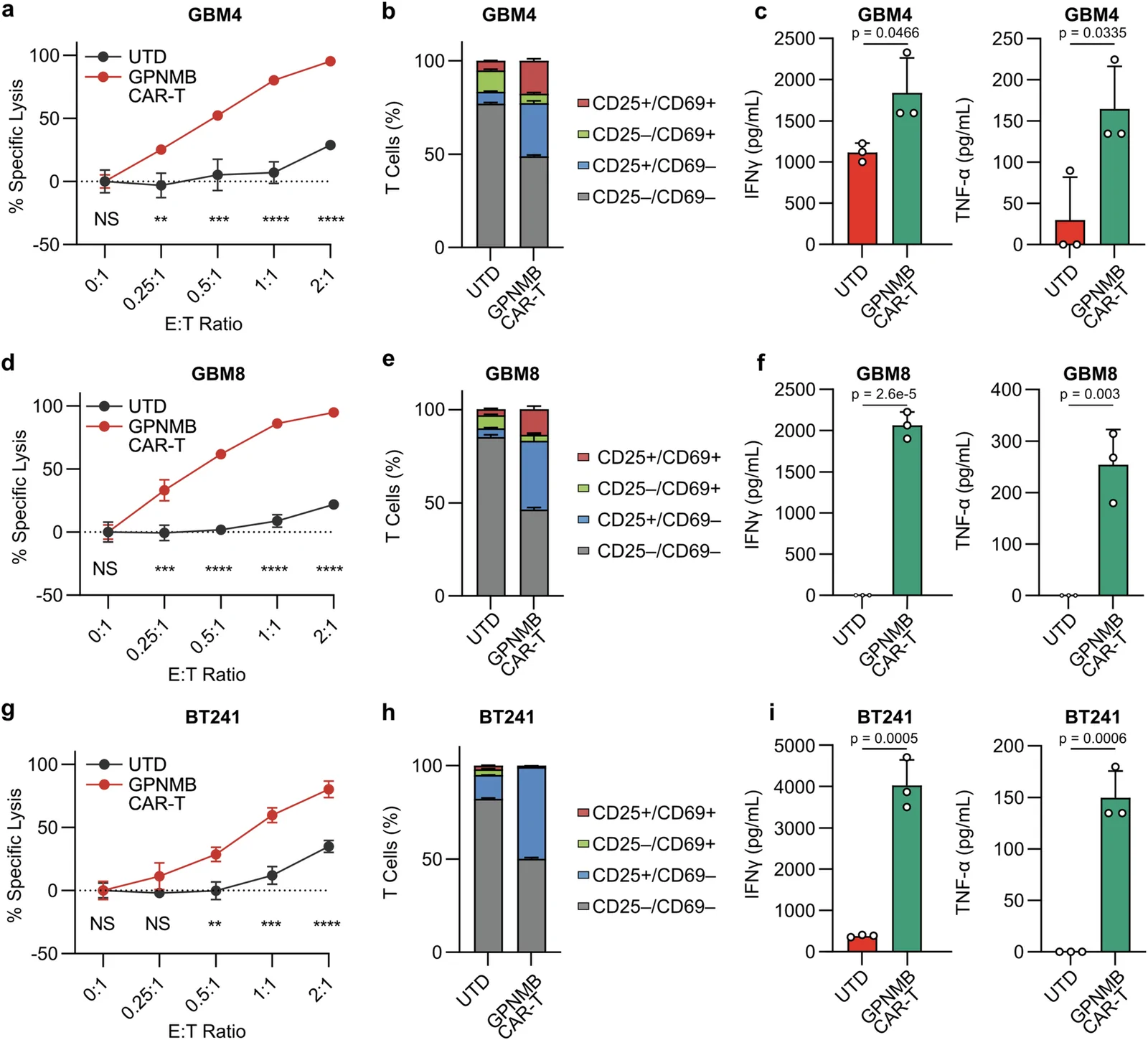
PC63-derived anti-GPNMB CAR-T cells demonstrate equivalent activity. (**a,d,g**) Percent specific lysis of ffluc-expressing GBM4 (a), GBM8 (d), and BT241 (g) targets after co-culture with PC63-derived untransduced (UTD) or anti-GPNMB CAR-T cells across effector:target (E:T) ratios after 48 h (mean ± s.d.; n = 4 experimental replicates). (**b,e,h**) Flow cytometry of T cell activation (CD25/CD69) following co-culture with GBM4 (b), GBM8 (e), or BT241 (h) at 1:1 E:T for 24 h, reporting the fraction of T cells in CD25/CD69 single or dual activation quadrants for UTD versus GPNMB CAR-T. (mean ± s.d.; n = 3 experimental replicates). (**c,f,i**) IFNγ and TNFα secretion measured by ELISA following co-culture of T cells with GBM4 (c), GBM8 (f), or BT241 (i) at 1:1 E:T for 24 h (mean ± s.d.; n = 3 experimental replicates). All *P* values from unpaired two-sided Student’s *t*-test (**P  <  0.01, ***P  <  0.001, ****P  <  0.0001, or as specified). Source data

Our data suggest that GPNMB supports malignancy by acting inherently to GBM cells and extrinsically to modulate the TME. Consequently, we explored whether GPNMB expression could be exploited therapeutically using CAR-T cell therapy. We utilized the single-chain variable fragment (scFv) from glembatumumab vedotin, a fully human IgG2 monoclonal antibody that was previously tested as an antibody–drug conjugate in multiple cancers^21–24^. The second-generation CAR design consisted of a CD8A signal peptide, glembatumumab vedotin scFv, CD8 hinge and transmembrane domain, 4-1BB costimulatory domain and CD3${\rm{\zeta }}$ signalling motif, along with a GFP reporter (Extended Data Fig. 3a). Following lentiviral packaging, we transduced T cells from three independent healthy blood donors to generate anti-GPNMB CAR-T cells (Extended Data Fig. 3b-c). Across T cell and GSC donors, GPNMB CAR-T cells mediated robust anti-tumour activity in vitro: CAR-T cells demonstrated potent dose-dependent cytotoxicity against several GPNMB^+^ GSC lines (Fig. 3a,b and Extended Data Fig. 3d), accompanied by upregulation of the activation markers CD25 and CD69 (Fig. 3c,d and Extended Data Fig. 3e) and significantly increased effector cytokine production of IFNγ and TNF after 24 h of co-culture with target cells (Fig. 3e,f and Extended Data Fig. 3f). This activity was consistent across all donor–target pairings (Extended Data Figs. 4 and 5). By contrast, CAR-T cells displayed significantly attenuated toxicity when challenged with *GPNMB*-knockout GSCs (Extended Data Fig. 3g,h).

We next evaluated whether this potency translated to established intracranial disease using orthotopic PDX models in immunodeficient NSG mice. Firefly luciferase (ffluc)-expressing GBM4 and GBM8 cells were implanted intracranially, enabling longitudinal assessment by bioluminescence imaging (BLI). Of note, treatment with two doses of GPNMB CAR-T cells resulted in complete clearance of tumour burden and durable disease control (Fig. 3g,h,k), with responses persisting beyond 160 days in 6 out of 7 GBM8-bearing mice and beyond 120 days in 6 out of 6 GBM4-bearing mice (Fig. 3i,j). These PDX outcomes show durable and curative anti-tumour activity.

Given our initial observation of *GPNMB* upregulation in CD133^−^ GSC subsets, we tested whether GPNMB CAR-T cells could be useful in the post-CD133 CAR-T cell landscape. Indeed, post-CD133 CAR-T-treated residual PDX tumours showed near-complete elimination of CD133^+^ tumour cells, but diffuse GPNMB positivity (Extended Data Fig. 3i,j) We therefore administered two doses of anti-GPNMB CAR-T cells to mice with tumours that recurred following CD133 CAR-T cell therapy. Despite initiating treatment at high tumour burden, anti-GPNMB CAR-T cells again achieved complete tumour clearance and superior control compared with CD133 CAR-T cells alone (Fig. 3l,m). Together, these preclinical findings support anti-GPNMB CAR-T cells as a highly effective therapy for GBM with activity in orthotopic PDX models and in tumours that persist following prior treatment pressure.

## GPNMB CAR-T cells are active in humanized PDXs

**Fig. 4:**
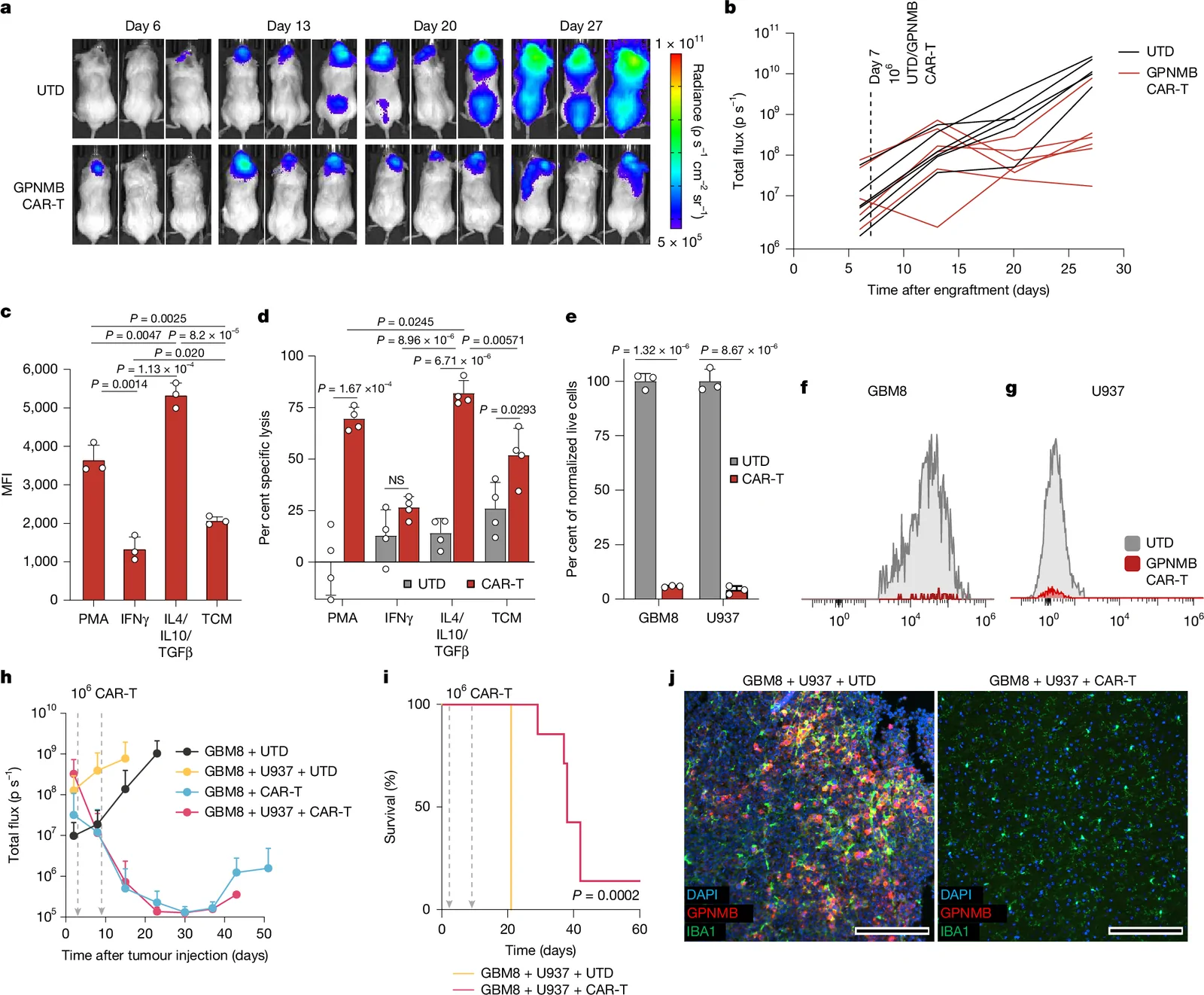
GPNMB CAR-T cells eliminate GPNMB^+^ tumour and myeloid cells. **a**,**b**, Representative IVIS images (**a**) and BLI measurements (**b**) of humanized NOG-EXL mice (*n* = 6 mice per cohort) bearing orthotopic recurrent GSCs (BT972) treated with CAR-T cells or untransduced (UTD) control cells. **c**, Flow cytometry analysis of GPNMB surface expression (measured as mean fluorescence intensity (MFI)) on U937 macrophages exposed to various stimuli. Data are mean MFIs ± s.d.; *n* = 3 technical replicates; unpaired two-sided Student’s *t*-test. **d**, Per cent specific lysis of conditioned U937-ffluc targets by UTD or GPNMB CAR-T cells at 1:1 E:T ratio for 48 h. Data are mean ± s.d.; *n* = 4 experimental replicates; unpaired two-sided Student’s *t-*test. **e**, Triple co-culture of GBM8-iRFP, U937 cells and UTD or GPNMB CAR-T cells at a 1:1:1 ratio for 24 h. Cell viability measured as percentage of live cells normalized to control. Data are mean ± s.d.; *n* = 3 experimental replicates; unpaired Student’s *t*-test. **f**,**g**, Representative histograms showing relative abundance of GSCs (**f**) and U937 cells (**g**) after co-culture with UTD control (grey) or GPNMB CAR-T cells (red). **h**,**i**, NSG mouse co-inoculation model: tumour bioluminescence (**h**) and Kaplan–Meier survival (**i**) for mice co-injected intracranially with 10^5^ GBM8-ffluc plus 10^5^ IL-4, IL-10 and TGFβ-conditioned U937 cells, followed by intracranial treatment with 10^6^ UTD controls or GPNMB CAR-T cells at the indicated time points. Data are mean BLI + s.e.m.; UTD: *n* = 8 mice; CAR-T: *n* = 6 mice; log-rank Mantel–Cox test. GBM8-ffluc-only UTD and GPNMB CAR-T cells mice from Fig. 3h are shown for reference. **j**, Multiplex immunofluorescence of end-point brains from UTD control-treated mice and time-matched CAR-T cell-treated mice, stained for GPNMB and IBA1 plus DAPI nuclear counterstain, demonstrating clearance of GPNMB^+^ tumour cells and GPNMB^+^IBA1^+^ macrophages. Scale bars, 200 μm. Source data

To test the efficacy of GPNMB CAR-T cells in vivo in a functional immune background while maintaining use of tissue from human patients, we utilized humanized NOG-EXL mice. NOG-EXL mice are transgenic NOG (NOD.Cg-*Prkdc*^*scid*^*Il2rg*^*tm1Sug*^) mice that express human IL-3 and GM-CSF, transplanted with CD34^+^ human haematopoietic stem cells, enabling reconstitution of both lymphoid and myeloid lineages^25^. We xenografted these mice with BT972-ffluc cells, then subsequently administered two rounds of untransduced (UTD) or anti-GPNMB CAR-T cells intracranially (Fig. 4a). Of the cohort treated with UTD T cells, two out of six mice experienced stable tumour progression week by week before growth continued. For comparison, four out of six mice in the GPNMB CAR-T cell cohort experienced substantial tumour reduction, including the two mice with the largest tumours (Fig. 4b). Despite individual variation, GPNMB CAR-T cell therapy demonstrated overall therapeutic efficacy against this humanized GBM model.

**Extended Data Fig. 6:**
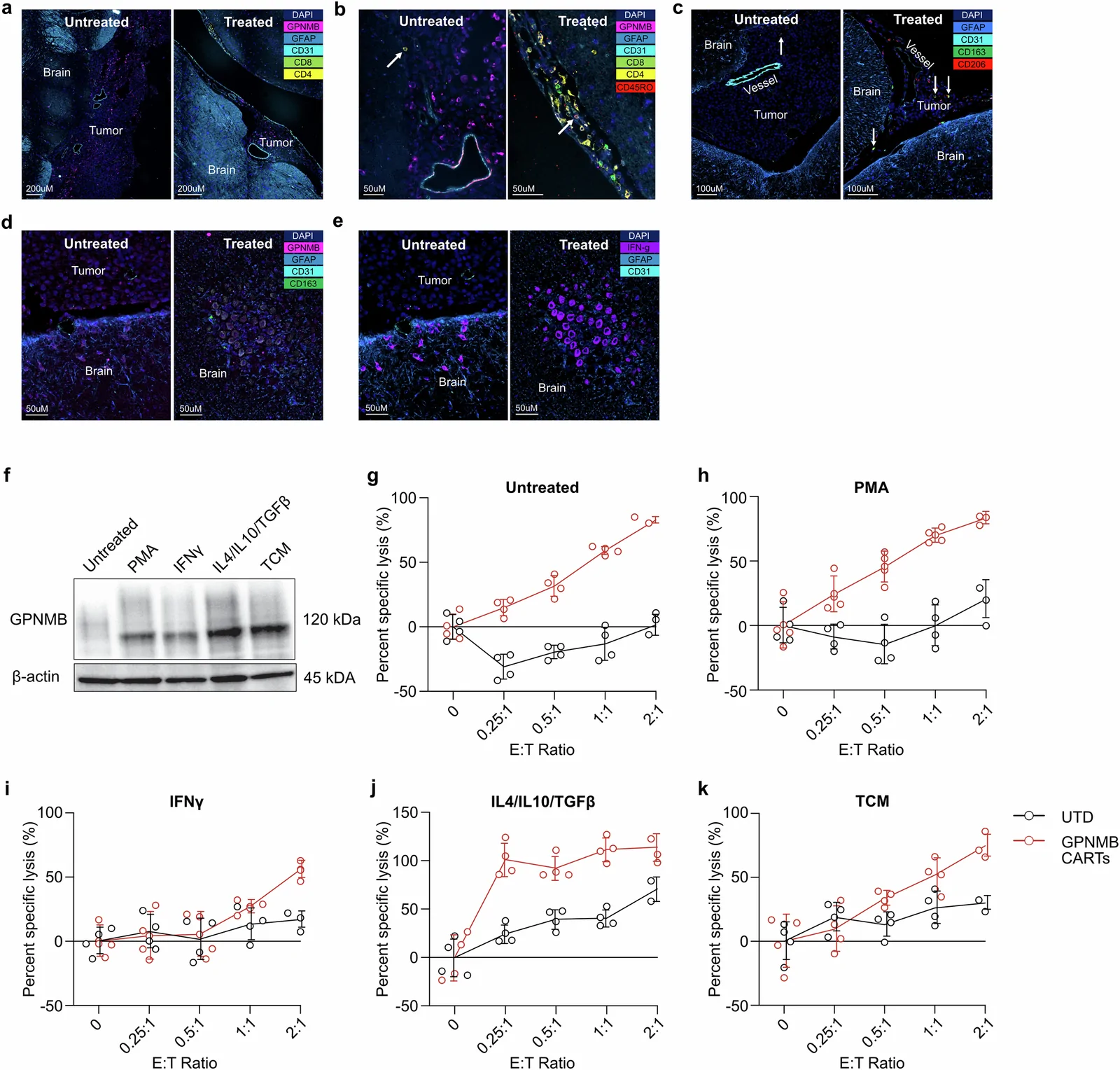
GPNMB CAR-T treatment reshapes the intracranial immune landscape and preserves cytotoxicity against cytokine-conditioned macrophages. (**a**) Sequential immunofluorescence of brains from humanized mice bearing BT972 tumors, comparing UTD versus CAR-T treated animals, stained for DAPI (nuclei), GFAP, CD31, CD8, and CD4, and GPNMB as indicated (scale bars, 200 µm). (**b**) Magnified view highlighting increased T cell infiltration in treated mice, including CD45RO (scale bars, 50 µm). (**c**) Sequential immunofluorescence for macrophage scavenger receptors CD163 and CD206 (arrows; scale bars, 100 µm). (**d-e**) Representative field highlighting CD163^+^ macrophages with increased cytosolic GPNMB foci and IFNγ staining in treated versus untreated brains (scale bars, 50 µm). (**f**) Immunoblot of GPNMB in U937 macrophages following conditioning with the indicated stimuli (Untreated, phorbol 12-myristate- 13-acetate [PMA], IFNγ, IL4/IL10/TGFβ, or tumor-conditioned media [TCM]), with GAPDH as a loading control. (**g-k**) Percent specific lysis of U937-ffluc targets by untransduced (UTD) or GPNMB CAR-T cells across E:T ratios following U937 conditioning with basal media (g), PMA (h), IFNγ (i), IL4/IL10/TGFβ (j), or TCM (k) (mean ± s.d., n = 4 experimental replicates). Source data

We utilized this model to visualize the spatial architecture of human GBM and the TME following CAR-T cell treatment using highly multiplexed IHC^26^. We sampled three UTD and three CAR-T cell-treated humanized mouse brains for GPNMB and a variety of immune cell markers. Brains treated with CAR-T cells showed nearly complete eradication of GPNMB^+^ tumour cells (Extended Data Fig. 6a), as well as enhanced CD4^+^ and CD8^+^ T cell infiltration, including GFP^+^ CAR-T cells and GFP^–^ endogenous T cells, and memory T cells (Extended Data Fig. 6b).

To determine whether the GPNMB CAR-T treatment overcame the immunomodulatory effect of TAMs, we checked whether the humanized mice had an immunosuppressive niche resembling human GBM. Indeed, CAR-T cell-treated mice displayed tumour eradication despite the presence of CD163^+^ macrophages (Extended Data Fig. 6c,d). TAMs displayed increased expression of the scavenger receptor CD206 (Extended Data Fig. 6c), suggesting active participation in phagocytosis and efferocytosis in the treated tumour regions. Closer inspection revealed increased cytoplasmic GPNMB^+^ foci (Extended Data Fig. 6d), as well as abundant IFNγ expression in CD163^+^ TAMs (Extended Data Fig. 6e). These observations suggest that post-treatment TAMs phagocytose GPNMB^+^ tumour cells and debris following CAR-T cell therapy.

## GPNMB CAR-T cells co-target tumour and TAMs

We next sought to further study the ability of GPNMB CAR-T cells to overcome the immunomodulatory effect of GBM TAMs. First, we examined whether GPNMB expression varies on the basis of the phenotypic plasticity observed in TAMs. We treated phorbol 12-myristate-13-acetate (PMA)-differentiated U937 macrophages with IFNγ, a combination of IL-4, IL-10 and TGFβ, or tumour-conditioned medium to mimic the spectrum of TAM phenotypes. Macrophages showed increased GPNMB expression when polarized with immunosuppressive and GSC-derived cytokines (Fig. 4c and Extended Data Fig. 6f). This led us to hypothesize that GPNMB CAR-T cells may exhibit preferential cytotoxicity toward such immunoregulatory macrophage populations. Indeed, GPNMB CAR-T cells spared IFNγ-primed macrophages but lysed those polarized by immunosuppressive or tumour-derived stimuli (Fig. 4d and Extended Data Fig. 6g–k).

**Fig. 5:**
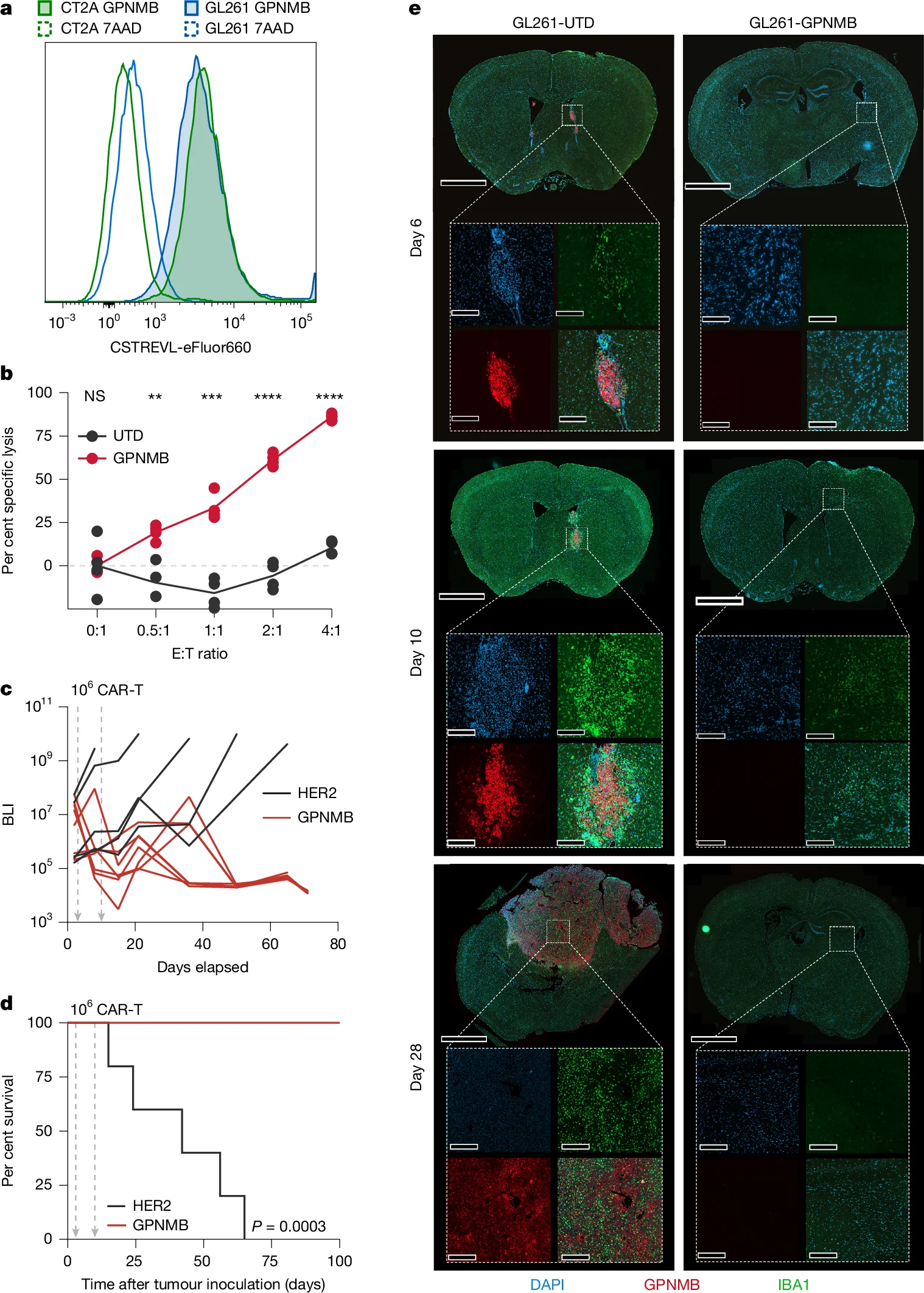
Murinized anti-mouse GPNMB CAR-T cells show control over syngeneic glioma models. **a**, Flow cytometry of surface mouse GPNMB (GPNMB) on CT2A and GL261 mouse glioma lines. **b**, Per cent specific lysis of GL261-ffluc target cells co-cultured with non-targeting (truncated HER2) or anti-mouse GPNMB mouse CAR-T cells across effector-to-target ratios after 48 h. Data are mean ± s.d.; *n* = 4; unpaired two-sided Student’s *t*-test. **c**,**d**, Syngeneic orthotopic GL261-ffluc trial following administration of HER2 (*n* = 5 mice) or mouse GPNMB (*n* = 7 mice) CAR-T cells in C57BL/6 mice at indicated time points: tumour bioluminescence (**c**) and Kaplan–Meier survival (**d**). *P* value from log-rank Mantel–Cox test. **e**, Immunofluorescence of intracranial tumours from control or mouse GPNMB CAR-T-treated mice at 6, 10 or 23 days post-tumour inoculation, stained with DAPI and for GPNMB and IBA1. Insets show magnified views of tumour-burdened regions around injection sites. Scale bars, 2 mm (whole-brain sections); 200 μm (insets). Source data

Next, we evaluated whether GPNMB CAR-T cells could simultaneously target tumour cells and TAMs in a direct co-culture. UTD or CAR-T cells were co-cultured with infrared fluorescent protein (iRFP)-expressing GBM8 and U937 cells, followed by quantification of each live cell population by flow cytometry. CAR-T cells effectively lysed both GBM8-iRFP tumour cells and macrophages with no hindrance in cytotoxicity against either target (Fig. 5f,g). These data indicate that GPNMB CAR-T cells can surmount the immunomodulatory effect of TAMs in vitro and simultaneously eliminate GPNMB^+^ tumour cells and immunoregulatory macrophages.

To gauge this effect in vivo, we established a GSC–macrophage co-inoculation model in NSG mice. Given the paucity of functional endogenous bone marrow-derived macrophages in NSG mice^27^, this strategy enables introduction of a defined immunosuppressive macrophage compartment while maintaining a human GBM xenograft. Consistent with the established pro-tumorigenic role of macrophages in GBM^28^, intracranial co-injection of GBM8-ffluc cells and immunosuppressive cytokine-conditioned U937 cells substantially accelerated tumour growth and shortened survival relative to macrophage-deficient (that is, GBM8-only) tumours (Fig. 4h). GPNMB CAR-T cell treatment induced profound tumour regression in the co-inoculated setting and significantly prolonged survival compared with UTD control-treated mice (Fig. 4h,i). To directly assess intracranial co-targeting, we performed multiplexed immunofluorescence on brains collected at end-point from UTD control-treated mice and time-matched CAR-T cell-treated mice. We observed near-complete clearance of both GPNMB^+^ tumour cells and GPNMB^+^IBA1^+^ macrophages (Fig. 4j). Evidently, GPNMB CAR-T cell therapy remained effective in the context of an enforced, macrophage-rich, tumour-promoting microenvironment and can eradicate GPNMB^+^ tumour and myeloid populations in vivo.

## scRNA-seq atlas of GPNMB^+^ CNS myeloid cells

**Extended Data Fig. 7:**
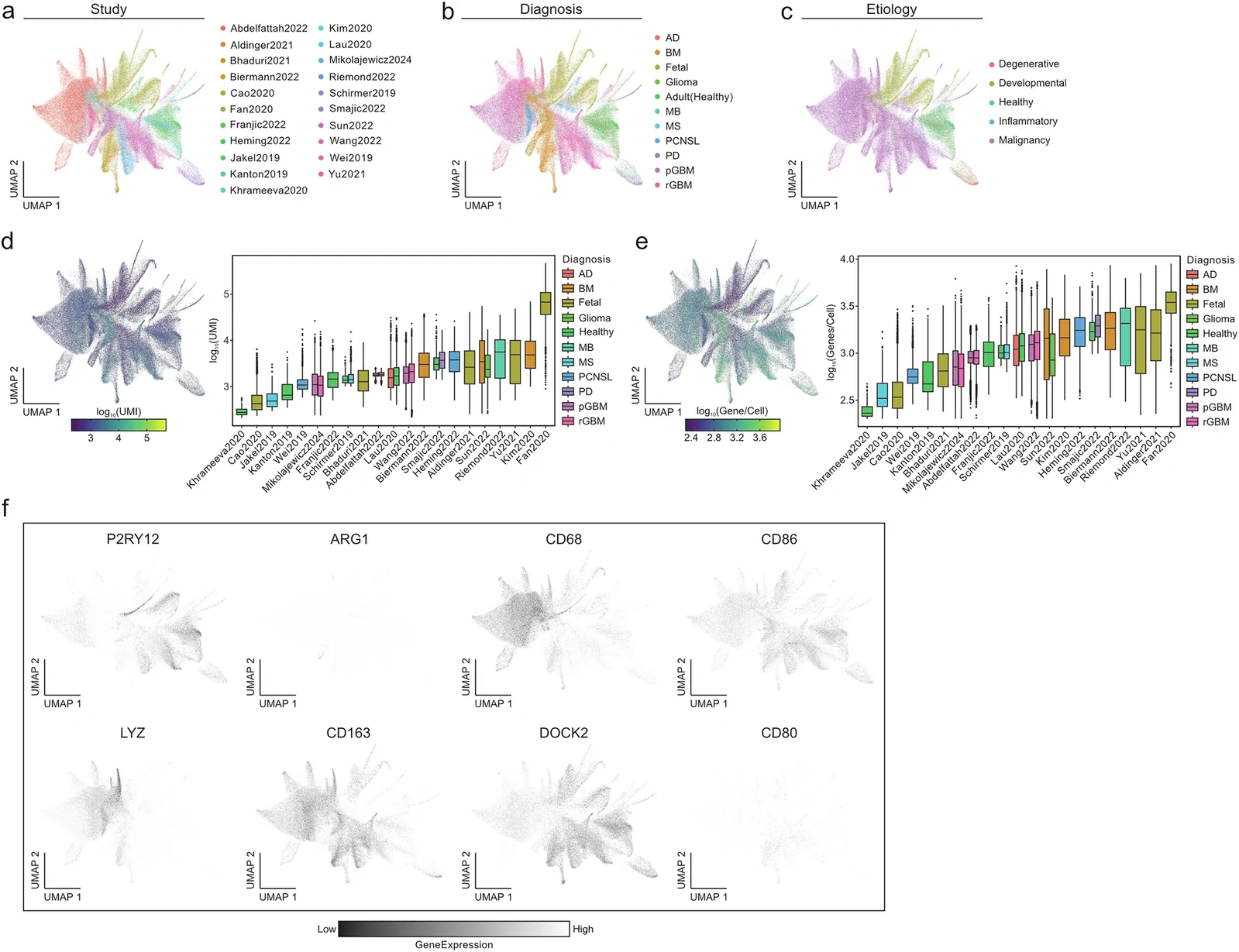
Characterization and quality control of the integrated myeloid scRNA-seq atlas. (**a**-**c**) UMAP of myeloid cells colored by study (**a**), diagnosis (**b**), and disease etiology (c) (**d**) Unique molecular identifiers (UMI) per cell visualized using UMAP (left) and boxplot (right), stratified by study and diagnosis. (**e**) Unique genes detected per cell visualized using UMAP (left) and boxplot (right), stratified by study and diagnosis. Centre line, median; box limits, IQR; whiskers, range within 1.5×IQR. (**f**) UMAP of select myeloid markers. Atlas comprises 279 samples and 207,997 myeloid cells across disease states: Alzheimer’s disease (AD; n = 10; 3929 cells), brain metastasis (BM; n = 45; 28,424 cells), fetal (n = 54; 29,484 cells), glioma (n = 4; 2141 cells), healthy (n = 26; 22,150 cells), medulloblastoma (MB; n = 13; 1841 cells), multiple sclerosis (MS; n = 13; 2006 cells), primary CNS lymphoma (PCNSL; n = 2, 9001 cells), Parkinson’s disease (PD; n = 5; 2698 cells), primary GBM (pGBM; n = 39; 37,502 cells), and recurrent GBM (rGBM; n = 68; 68,821 cells). Source data

**Extended Data Fig. 8:**
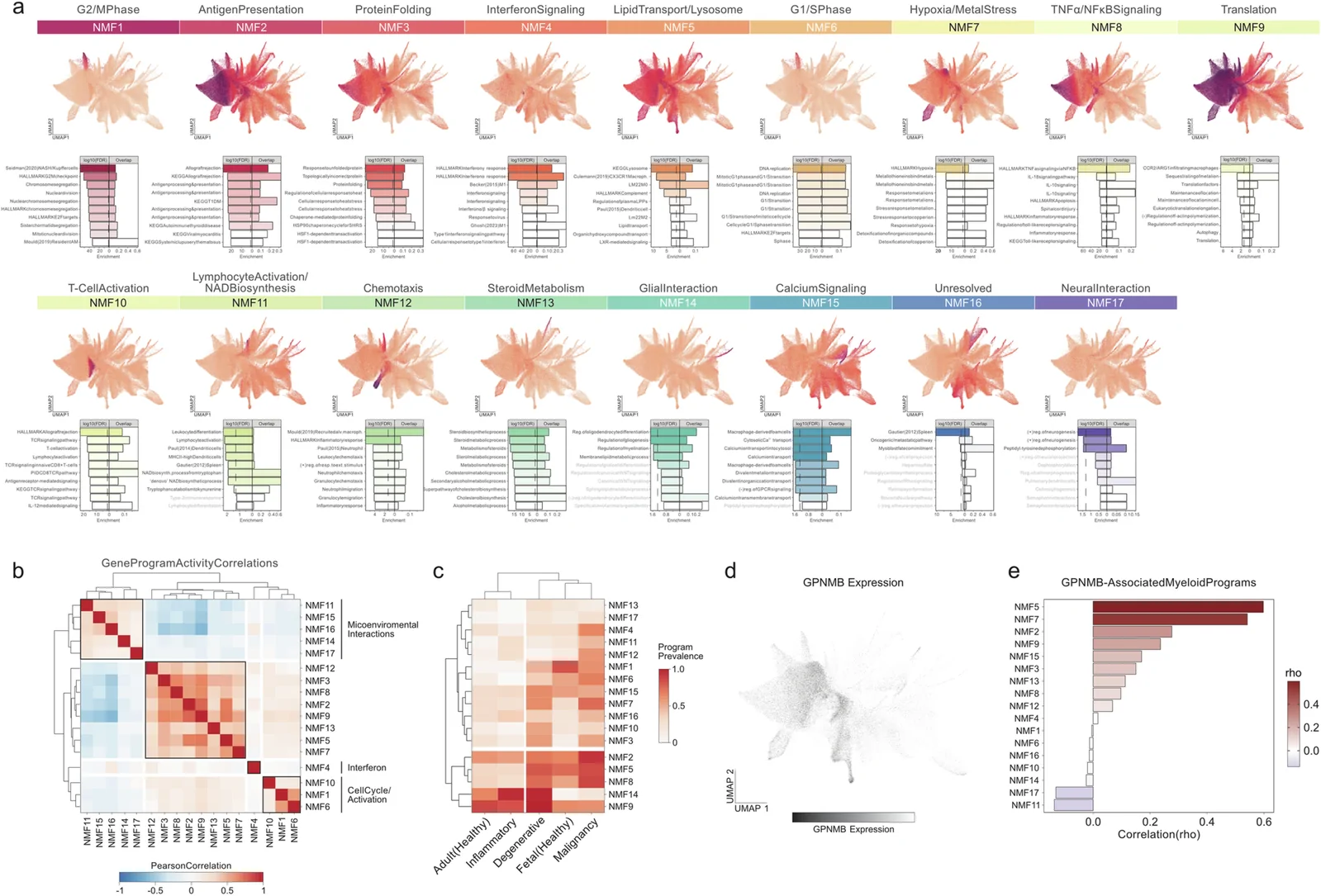
Unsupervised gene program discovery identifies GPNMB- associated programs. (**a**) NMF analysis identified 17 gene programs (NMF1–17) in myeloid cells. UMAPs (top) and bar plots (bottom) show each program’s activity and functional annotation, respectively. Annotations were performed using hypergeometric overrepresentation analysis with a 5% FDR threshold. (**b**) Heatmap of Pearson correlations between myeloid gene program activities, with hierarchically clustered groups. (C) Heatmap of gene program prevalence among patients in each disease etiology group. (D) UMAP plot of GPNMB expression. (E) NMF gene programs ranked by correlation with GPNMB expression. Abbreviations: NMF, non-negative matrix factorization; UMAP, uniform manifold approximation and projection.

To gain functional insight on GPNMB^+^ macrophages, an integrative analysis was performed of scRNA-seq data from myeloid populations across various CNS contexts, including both developmental aetiologies and disease-related aetiologies. UMAP plots demonstrated distinct clustering patterns on the basis of study (Extended Data Fig. 7a), diagnosis (Extended Data Fig. 7b) and aetiology (Extended Data Fig. 7c). Quality control metrics validated the robustness of the scRNA-seq datasets, with sufficient unique molecular identifier (UMI) counts (Extended Data Fig. 7d) and gene detection per cell (Extended Data Fig. 7e) across all studies. Additionally, canonical myeloid markers were projected onto the UMAP to confirm accurate representation of heterogenous myeloid cell populations within the atlas (Extended Data Fig. 7f). Unsupervised gene programme discovery using non-negative matrix factorization (NMF) identified 17 distinct gene programmes that can be functionally annotated (Extended Data Fig. 8a). Correlations between gene programmes showed hierarchical clustering, suggesting functionally related groups of programmes (Extended Data Fig. 8b). Furthermore, the prevalence of each gene programme varied across aetiologies (Extended Data Fig. 8c). For example, macrophages in malignant diseases were enriched for antigen presentation (NMF2) and lipid transport (NMF5), fetal brain macrophages for cell cycling (NMF1) and neuroinflammatory macrophages for glial interaction pathways (NMF14).

**Extended Data Fig. 9:**
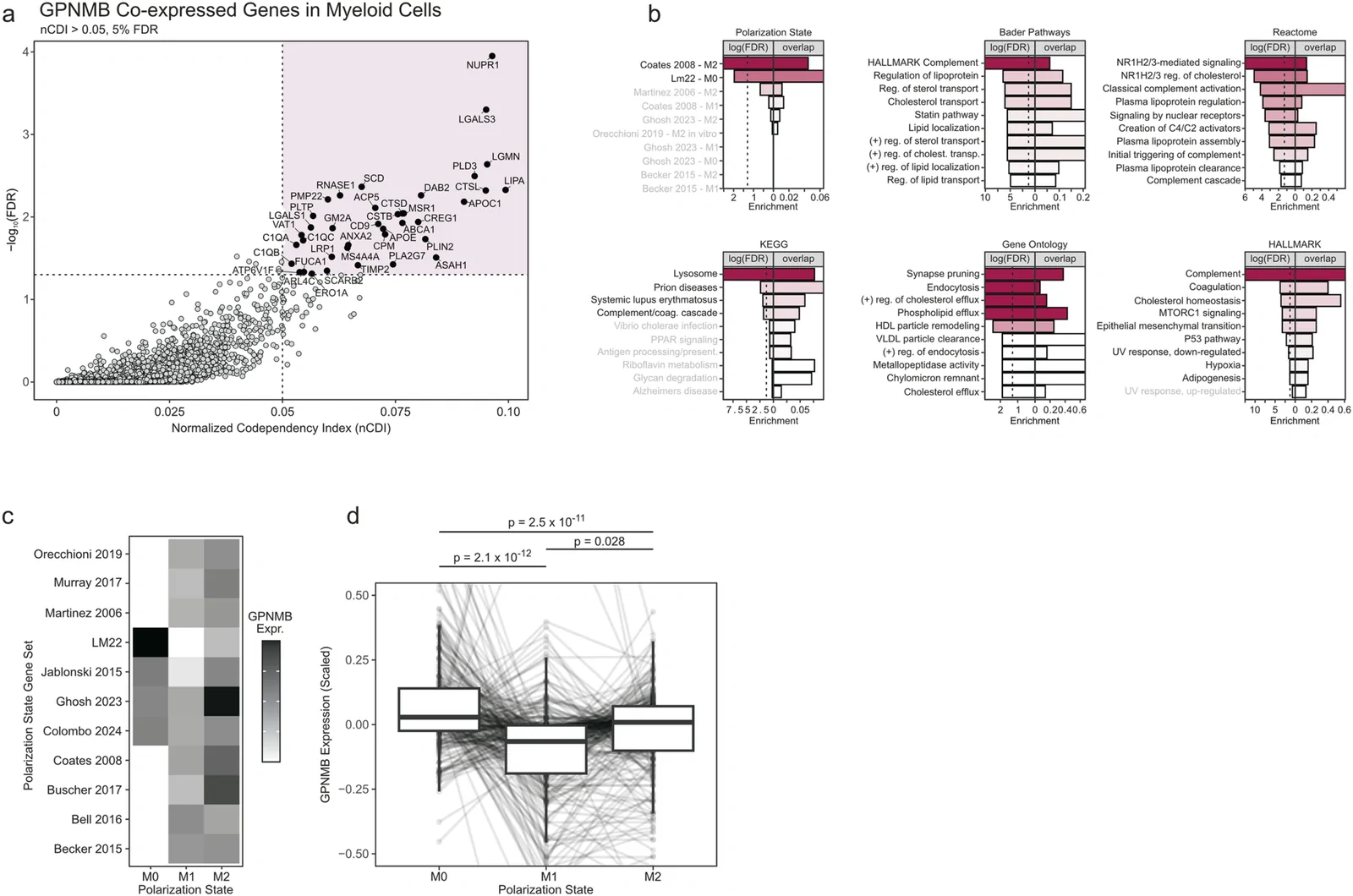
GPNMB expression correlates with lipid metabolism and polarization phenotypes. (**a**) Scatterplot of GPNMB co-expressed genes identified using normalized co- dependency index (nCDI). Significance was determined as nCDI > 0.05 and 5% FDR. **(b)** Functional annotation of GPNMB-associated genes determined using hypergeometric overrepresentation analysis, with Benjamini-Hochberg (BH)-adjusted *P* values. **(c)** Heatmap of GPNMB expression in myeloid cells grouped by polarization state. **(d)** Boxplot of GPNMB expression in M0 (n = 279 samples; 91,542 cells), M1 (n = 270 samples; 59,310 cells), and M2-like (n = 277 samples, 57,145 cells) myeloid cells, with lines representing expression patterns within individual patients. Centre line, median; box limits, IQR; whiskers, range within 1.5×IQR. Overall group differences were assessed using a Kruskal-Wallis test, followed by pairwise *t*-tests with BH correction for composite-state contrasts.

Ranking programmes on the basis of correlation with GPNMB expression revealed that lipid transport and hypoxia are most associated with GPNMB (Extended Data Fig. 8d,e). Further examination of genes that were co-expressed with GPNMB revealed several genes involved in lipoprotein formation and transportation (*APOC1*, *APOE*, *PLTP* and *LRP1*) (Extended Data Fig. 9a). GSEA further highlighted cholesterol and lipoprotein transport, and liver X receptor signalling pathways enriched in co-dependent genes (Extended Data Fig. 9b).

To assess our previous findings of GPNMB upregulation in response to immunosuppressive signals, we analysed *GPNMB* expression in relation to simplified myeloid cell polarization states. Indeed, *GPNMB* expression correlated more strongly with immunosuppressive M2-like gene signatures (Extended Data Fig. 9c), and less with pro-inflammatory M1-like gene signatures in individual patients (Extended Data Fig. 9d). This atlas comprehensively supports a role for GPNMB^+^ myeloid cells in lipid metabolism and export, and their association with specific polarization states, providing further insight into the pathophysiological role of GPNMB^+^ macrophages in disease progression.

## Murinized GPNMB CAR-T cells are curative

**Extended Data Fig. 10:**
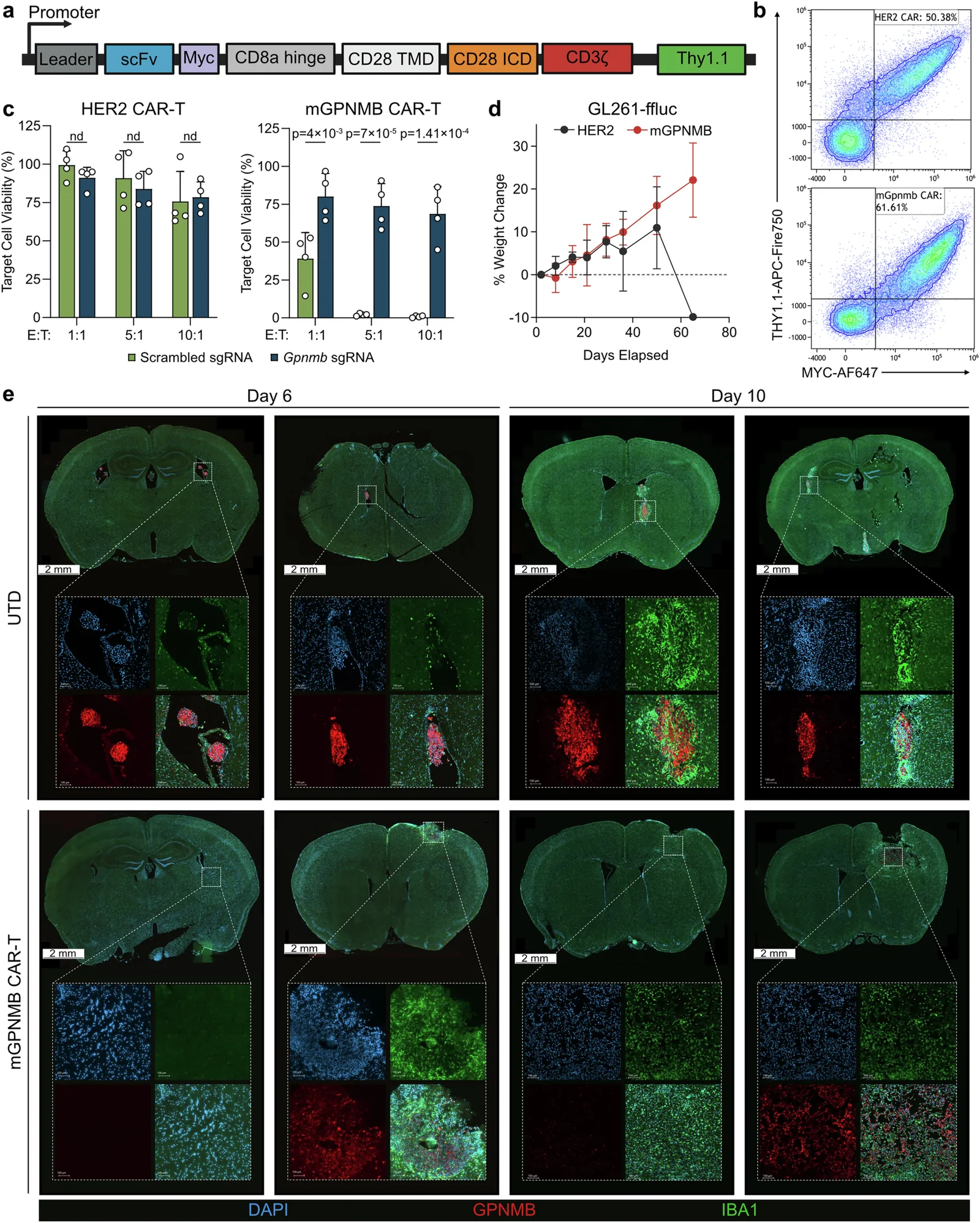
Generation and testing of anti-mouse GPNMB CAR-Ts. **(a)** Schematic of the murine anti-GPNMB CAR cassette, including Thy1.1 as a surface reporter. Created in BioRender; Venugopal, C. https://BioRender.com/dfzup0y (2026). (**b**) Flow cytometry demonstrating CAR expression in transduced murine T cells (Thy1.1 versus Myc tag; gating shown). (**c**) In vitro target cell viability of B16F10 cells edited with a scrambled or *Gpnmb*-targeting sgRNA and co-cultured with murine non-targeting (HER2) versus anti-GPNMB CAR-T cells (mean ± s.d., n = 4 experimental replicates, *P* values from unpaired two-sided Student’s T test). (**d**) Percent body weight change over in vivo study course for HER2 (n = 5 mice) versus anti-GPNMB CAR-T (n = 7 mice)–treated mice (mean ± s.d. as plotted). (**e**) Representative whole-brain immunofluorescence at the indicated days post intracranial tumor inoculation from control or anti-GPNMB CAR-T–treated mice, stained for DAPI, GPNMB, and IBA1; insets show tumor-burdened regions surrounding injection sites (scale bars, 2 mm whole brain and 100 μm insets).

Finally, to test the curative potential of GPNMB CAR-T therapy in an immunocompetent model, we generated and validated a murinized anti-mouse GPNMB CAR-T cell using the binder sequence derived from the commercial antibody clone CSTREVL (Extended Data Fig. 10a,b). We confirmed specificity and on-target activity using GPNMB^+^ B16F10 melanoma cells (Extended Data Fig. 10c), and demonstrated potent cytotoxicity against GL261 cells in vitro (Fig. 5a,b). In C57BL/6 mice bearing orthotopic GL261 tumours, murinized GPNMB CAR-T cell treatment again elicited a strong therapeutic response and achieved durable, long-term tumour control exceeding 100 days (Fig. 5c,d) with no overt toxicity (Extended Data Fig. 10d).

To directly assess effects on the TME, we analysed brains from control and treated mice at early post-treatment time points (6 and 10 days post-tumour inoculation), as well as at end-point of control mice or time-matched treated mice. Consistent with our observations in the PDX co-injection setting, we found robust depletion of GPNMB^+^IBA1^−^ tumour cells and GPNMB^+^IBA1^+^ macrophages following CAR-T cell treatment (Fig. 5e). These studies demonstrate that GPNMB CAR-T cell therapy retains potent, curative efficacy in an intact immune system and mediates dual targeting of tumour and myeloid compartments within a representative syngeneic GBM microenvironment.

## Discussion

We identified GPNMB as a therapeutically tractable antigen in GBM, that is present in both treatment-resistant malignant cells and TAMs. We generated anti-GPNMB CAR-T cells showing robust activity across multiple preclinical settings, with curative responses in most tested models. Together, these findings establish a dual-compartment targeting paradigm for GBM by producing a single CAR that can engage both the malignant population that sustains growth and the myeloid compartment that enforces immunosuppression.

GBM has remained a challenging setting for adoptive cell therapy. CAR-T cell trials have shown that intracranial delivery is feasible and can induce radiographic response^29–31^, yet disease control is often transient, with progression driven by antigen loss, limited persistence and myeloid-mediated immunomodulation within the TME^4,32^. As our understanding of TAM diversity has evolved, GPNMB has emerged as a marker for immunoregulatory macrophages in GBM^33,34^. Consistent with these reports, our scRNA-seq of samples from human patients with GBM identified the myeloid population as a major source of *GPNMB* expression. Our scRNA-seq CNS myeloid atlas revealed that GPNMB^+^ TAMs show enhanced lipoprotein metabolism signatures and are strongly associated with immunosuppression, directly confirming a recent study^35^ that identified identical lipid transport phenotypes in a GPNMB^hi^ TAM population in mouse glioma models. Additional reports describe paracrine circuits in which interactions between GSCs and TAMs induce GPNMB expression and reinforce tumour-promoting phenotypes in the TAMs^36^, and in which GPNMB^+^ TAMs crosstalk with fibroblasts to promote immunosuppression and vascular fibrosis^37^. Studies using *Gpnmb*-knockout mice, generally in the context of obesity, largely agree that GPNMB loss of function leads to heightened inflammatory phenotypes in obese mice^38,39^, further supporting the role of GPNMB^+^ macrophages as an immunosuppressive population.

Targeting TAMs with CAR-T cells has been shown to promote anti-tumour immunity^40–43^, although there may be added benefit to dual tumour–myeloid targeting: GPNMB CAR-T cells simultaneously overcame immunomodulation and maintain cytotoxicity in macrophage-inclusive assays, including both native syngeneic and GSC–macrophage co-inoculation studies. Additionally, intratumoural heterogeneity is another major driver of therapeutic escape, and multi-antigen strategies, such as the CD133 co-targeting approach used here, may further reduce the probability of relapse.

GPNMB has previously been implicated in tumour progression across multiple cancer types, supporting its plausibility as a therapeutic target. Its oncogenic roles—proliferative signalling^44–46^, extracellular matrix remodelling^47^ and activation of epithelial–mesenchymal transition programmes^48^—led to clinical trials testing glembatumumab vedotin in multiple cancers and showing safety but not efficacy^21–24^. In GBM, the relevance of GPNMB is reinforced by its association with mesenchymal biology: Neftel et al.^20^ described a recurring set of GBM transcriptional programmes that can co-exist within individual tumours. Of these states, MES-like cells are often seen as the most aggressive and treatment refractory^49,50^. In parallel, MES-like programmes can be promoted by immune and myeloid-derived cues^51^. Recent scRNA-seq and spatial transcriptomic studies have identified GPNMB^hi^ TAMs that promote proneural-to-MES transitions^52^ and co-localize with MES-like niches in glioma^53^. Consistent with this, in our scRNA-seq analysis of *Gpnmb*-knockout versus wild-type mouse gliomas, *Gpnmb* loss was associated with a shift toward developmental (NPC-like and OPC-like) and astrocyte-like transcriptional states, but not MES-like states, suggesting *Gpnmb*-dependent myeloid–tumour interactions contribute to maintenance of MES-like malignant programmes.

Although our study demonstrates strong preclinical efficacy, it prompts translational refinement. First, delivery route is likely to be a key determinant of clinical performance, and locoregional administration offers a rational strategy to maximize tumour exposure while limiting toxicity as GPNMB expression is low in normal brain tissue, although further study is needed on potential CNS toxicity and long-term safety evaluation of myeloid targeting. Second, although our current CAR achieves strong efficacy, advanced CAR engineering may further facilitate clinical translation, such as targeted CAR delivery into the *TRAC* locus to produce an autologous, ‘off-the-shelf’ CAR^+^ TRAC-knockout T cell product, and logic-gated circuits to further increase tumour selectivity^54,55^. Multi-antigen^31^ or tandem^56^ CAR-T cells directed against GBM-associated antigens could be combined with the GPNMB-targeting module to preserve dual-compartment activity while broadening malignant cell coverage. Armored CARs that deliver cytokines (such as IL-12 (ref. ^42^), IL-15 (ref. ^57^) or IL-18 (ref. ^58^) or checkpoint blockade^59^ may further counter immunosuppression.

Anti-GPNMB CAR-T cell therapy can prevent the ability of GBM cells to evade immune detection and pleiotropic cytotoxicity of GBM cells and their cognate TME presents a new and promising therapeutic strategy for myeloid-rich cancers such as GBM with currently limited treatment options. More broadly, our findings support a treatment paradigm that addresses GBM as a coupled tumour–myeloid system, in which durable control requires simultaneous engagement of malignant programmes and the immunosuppressive myeloid niche that sustains them.

## Methods

### Cell lines and patient-derived GSC cultures

Human GBM samples were obtained from patients who provided written informed consent for tissue collection and use in research and publication, under the approval of the Hamilton Health Sciences and McMaster Health Sciences Research Ethics Board (no. 16078). Tumour samples were processed using established protocols^60^. GBM cells were cultured in Neurocult Complete (NCC) medium, a commercially available serum-free NSC medium (STEMCELL Technologies, 05751), supplemented with human recombinant epidermal growth factor (20 ng ml^−1^; STEMCELL Technologies, 78006), basic fibroblast growth factor (20 ng ml^−1^; STEMCELL Technologies, 78006), heparin (2 μg ml^−1^; STEMCELL Technologies, 07980) and antibiotic–antimycotic solution (1×; Wisent, 450-115-EL). NSCs were cultured and maintained using similar protocols as described previously^61^. SK-MEL-2 cells, MDA-MB-231 and HEK293T cells were purchased from the American Type Culture Collection (ATCC) and grown in Dulbecco’s Modified Eagle Medium (DMEM) supplemented with 10% FBS and 1% non-essential amino acids (NEAA; Thermo Fisher, 11140050). NHAs were purchased from ATCC and grown in DMEM/F12 medium (Gibco, 11320033) supplemented with 10% FBS, and epidermal growth factor and fibroblast growth factor as above.

### Animal studies

All animal experiments were conducted in compliance with the ethical guidelines approved by the Animal Use Protocols (22-12-38) of the McMaster University Central Animal Facility. Mice were housed in pathogen-free, temperature-controlled, 12 h light and dark cycle environment and were fed ad libitum. Intracranial injections were performed on 6–12-week-old NOD/SCID gamma (NSG) or C57BL/6 mice, following previously described methods^60^. GBM4, GBM8 and MBT06 cell lines (10^6^ cells per mouse) or GL261 cells (10^5^ cells per mouse) were suspended in 10 μl PBS and injected into the right frontal lobe using a Hamilton syringe (Hamilton, 7635-01). A burr hole, 2 mm posterior to the coronal suture and 3 mm lateral to the sagittal suture, was drilled for intracranial access^60^. At the humane end-point, mice were euthanized, perfused with 10% formalin, and the brains were sectioned into 2 mm slices using a brain matrix for paraffin embedding and haematoxylin and eosin (H&E) staining. Digital images were captured using an Aperio Slide Scanner (Leica Biosystems) and analysed with ImageScope v11.1.2.760 software. Kaplan–Meier survival analyses were based on the time from surgery to end-point. For humanized studies, NOG-EXL mice (Taconic, NOD.Cg-*Prkdc*^*scid*^*Il2rg*^*tm1Sug*^Tg(SV40/HTLV-IL3,CSF2)10-7Jic/JicTac, 13395-F) were purchased and utilized as outlined above. Intracranial injections followed the previously described protocol for the BT972 cell line (10^6^ cells per mouse). Mice were monitored post-injections, and survival data were analysed using the Kaplan–Meier method.

### RNA-seq and differential gene expression ranking

Cells were subjected to RNA extraction, followed by RNA sequencing on the Illumina HiSeq 2500 platform. Four comparative analyses were performed: (1) GBM CD133^+^ versus CD133^−^; (2) NSC CD133^+^ versus CD133^−^; (3) CD133^+^ NSC versus CD133^+^ GBM; and (4) CD133^−^ NSC versus CD133^−^ GBM. Data filtering was performed using a CPM threshold of 3.5. Multidimensional scaling plots indicated clear sample separation across all comparisons. A smear plot analysis confirmed consistent gene expression patterns without significant artifacts at this CPM threshold. DEGs were identified using the quasi-likelihood *F*-test in edgeR, chosen due to its stringency and appropriateness for datasets with minimal sample sizes (*n* = 4, with ≥2 per group). Genes were ranked on the basis of *P* values and fold changes using the formula: ranking score = sign(log(fold change)) × –log_10_(*P* value), where the sign(log(fold change)) indicates the direction of expression change (positive for upregulation, negative for downregulation), and –log_10_(*P* value) reflects the significance level. Genes were ranked from highest upregulation to highest downregulation, with rankings exported as.rnk files for GSEA.

### GSEA and enrichment mapping

GSEA was conducted for all four comparisons using the.rnk files and the Human_GOBP_AllPathways_with_GO_iea_December_24_2015_symbol.gmt gene set. A total of 1,000 permutations were performed using a random seed of 349. Comparative GSEA results for the four analyses were compiled in Pathway.xlsx, including normalized enrichment scores and false discovery rate (FDR) *q*-values. Differences in gene filtering between the comparisons necessitated reanalysis of the RNA-seq data using only protein-coding genes, allowing consistent gene sets across all analyses. Owing to high similarity between: (1) CD133^+^ versus CD133^−^ in NSC (GSEA2) and GBM (GSEA1); and (2) NSC versus GBM in CD133^+^ (GSEA3) and CD133^−^ (GSEA4), combined enrichment maps were generated (map A: GSEA1 and GSEA2; map B: GSEA3 and GSEA4). These maps were constructed using a Jaccard coefficient of 0.25 (for edges) and an FDR *q*-value cutoff of 0.0001 (for nodes). Cytoscape files (.cys) containing both stringent and relaxed conditions (FDR *q*-value < 0.1, *P* value < 0.05) are available. To ensure direct comparability across the GSEA analyses,.rnk files were regenerated using all protein-coding genes without CPM filtering. *z*-scores were calculated for each pathway on the basis of the direction of enrichment and nominal *P* values. Pathways with significant differences (*P* < 0.01) were identified, and corresponding enrichment maps were generated.

### Interrogation of public databases

Publicly available datasets were incorporated using the GEPIA2 interface to compare gene expression correlations between GBM samples from TCGA and normal brain tissue from the GTEx portal, following established methods^62^.

### Flow cytometry

Cells were dissociated using TrypLE (Thermo Fisher, 12605010), resuspended in PBS containing 2 mM EDTA (Thermo Fisher, AM9260G) and stained with anti-human GPNMB-PE (Invitrogen, HOST5DS, 12-9838-42, 1:20) or anti-mouse GPNMB-eFluor 660 (Invitrogen, CTSREVL, 50-5708-82, 1:20) on ice for 45 min. Following washes, stained cells were analysed on a CytoFLEX flow cytometer (Beckman Coulter), with dead cells excluded using 7-AAD viability dye (1:100 dilution; Beckman Coulter, A07704). Compensation was performed using mouse IgG CompBeads (BD Biosciences, 552843). Cells were gated on unstained controls.

### Immunohistochemistry

Formalin-fixed mouse brains were sectioned coronally into 6–7 slices, then paraffin-embedded and mounted onto slides. Slides were deparaffinized in xylene (2 × 5 min) and rehydrated through graded ethanol (100% ethanol, 2 × 3 min; 95% ethanol, 1 × 3 min; 70% ethanol, 1 × 3 min). Antigen retrieval was performed in Tris-based antigen unmasking solution (Vector Laboratories, H-3302-251; 1:100), slides were heated in a microwave, then quenched by incubation in 10% methanol dissolved in H_2_O_2_ for 10 min. Slides were blocked for 1 h at room temperature on a shaker using 3% milk, 1% BSA in TBS. Primary antibodies were diluted in blocking buffer and applied overnight at 4 °C (rabbit anti-CD133, D2V8Q, CST, 64326 T, 1:200; goat anti-hGPNMB, R&D AF2550, 3:500). HRP-conjugated secondary antibody was prepared as a 1:1 dilution of secondary reagent (Donkey Anti-Rabbit IgG HRP, ab205722; Donkey Anti-Goat IgG HRP, Abcam, ab214881) and applied for 2 h at room temperature, followed by development with DAB (Vector Laboratories, SK-4100). Slides were counterstained with haematoxylin, blued in ammonia water (20 s) dehydrated through graded ethanol, and cover slipped with xylene-based mounting medium. Slides were cured overnight prior to imaging or whole-slide scanning with a Leica Aperio Scanscope XT. Images were processed and positive cells quantified with QuPath^63^. Tumour-burdened areas were marked using H&E sections across all coronal sections, and cells were quantified in all regions of interest.

### Immunofluorescence

Tissue sections were deparaffinized and rehydrated as with IHC. Antigen retrieval was performed in Tris–EDTA buffer (10 mM, pH 9.0) supplemented with 0.05% Tween-20 by microwaving (3 min on high, then 15 min on low) with slides fully submerged, followed by cooling at room temperature for 30 min. Slides were permeabilized in 0.05% TBS-T (20 min). Tissue was circumscribed with a hydrophobic barrier pen and blocked with CAS-Block (Invitrogen, 00-8120) for 10 min at room temperature in a humidified chamber. Primary antibodies diluted in blocking solution were applied overnight at 4 °C (rabbit anti-IBA1, Abcam EPR16588, 1:1,000; goat anti-mGPNMB, R&D AF2330, 1:500, goat anti-hGPNMB, R&D AF2550, 3:500). Fluorophore-conjugated secondary antibodies (donkey anti-rabbit AF488, Invitrogen, A-21206; donkey anti-goat AF594, Invitrogen, A-11058, chicken anti-goat AF647, Invitrogen, A-21469) diluted 1:500 in blocking solution were applied for 2 h at room temperature. Slides were mounted with DAPI aqueous mounting medium (Abcam, ab104139). Slides were imaged with a BioTek Cytation 5 Cell Imaging Multimode Reader at 20× objective, with brightness and contrast set in comparison to a 2° antibody-only condition. Images were processed and saved using ImageJ^64^. Whole-brain slices were scanned using a ZEISS Axioscan 7 at 20× objective at the McMaster Centre for Advanced Light Microscopy. Images were processed equally using ZEISS ZEN Lite.

### Generation of gene-knockout constructs

sgRNAs targeting *AAVS1* (GGGGCCACTAGGGACAGGAT) and human *GPNMB* (KO-A: AATGATGGTACAGACCTCCG, KO-B: AGGAATCCTACTCAGCTCCA) mouse *Gpnmb* (GAAAGUCUCUGCGGGGUCCU, AAAGGGCCUGGCCCAUCAUU and UCACGCUUGGCAGCCUGGAG) were obtained from the TKOv3 library^65^ and cloned into lentiCRISPRv2 constructs (Addgene #52961). Successful ligations were packaged independently into lentiviruses using second-generation packaging constructs. In brief, HEK293T cells were seeded at 1.5 × 10^7^ cells per T75 flask and incubated overnight in high-glucose DMEM medium with 2 mM L-glutamine and 1 mM sodium pyruvate (Thermo Fisher,11995065), supplemented with 1% non-essential amino acid solution (Thermo Fisher, 11140050) and 10% fetal bovine serum (Gibco, 12483020). The following day, the HEK293T medium was replaced with viral harvesting medium consisting of HEK medium supplemented with 10 mM HEPES (Thermo Fisher, 15630080) and 1 mM sodium butyrate (Sigma Aldrich, 303410). Transfection was conducted with a mixture of pMD2.G (VSV-g; 2.6 μg; Addgene #12259), pRSV-REV (REV; 2.6 μg; Addgene #12253), pMDLG/RLE (gag/pol; 5.3 μg; Addgene #12251), transfer plasmid (10.6 μg), polyethylenimine (63.5 μg; Sigma Aldrich, 408719) in 1.3 ml of Opti-MEM. After incubating for 15 min at room temperature, PEI/DNA mixture was added dropwise to T75 flasks. Viral supernatants were collected 48 h after transfection and then concentrated using ultracentrifugation (20,000 rpm for 2 h at 4 °C) before being snap-frozen and stored at −80 °C.

### Cell proliferation assays

Single cells were plated in 96-well plates at 1,000 cells per well in 200 μl of medium and incubated at 37 °C, 5% CO_2_ for 5 days. Presto Blue (20 μl; Thermo Fisher, A13262) was added 4 h prior to fluorescence readout using a FLUOstar Omega Microplate Reader with 544 nm excitation and 590 nm emission wavelengths. Proliferation was calculated by subtracting the background fluorescence from medium-only control wells.

### Western blotting

Cell pellets were lysed in RIPA buffer (50 mM Tris-HCl pH 8.0, 150 mM NaCl, 1% NP-40, 0.5% sodium deoxycholate, 0.1% SDS) supplemented with HALT protease/phosphatase inhibitor cocktail (Thermo Fisher, 78440) for 30 min at 4 °C. Lysates were clarified by centrifugation (14,000*g*, 15 min, 4 °C), and protein concentration was determined by Bradford assay (Bio-Rad). Equal amounts of protein were resolved on 4–12% Bis-Tris gradient gels (Novex, Invitrogen) and transferred to PVDF membranes (Immobilon-P, Millipore) at 200 mA for 2 h. Membranes were blocked in 5% BSA for 1 h at room temperature and incubated with primary antibodies overnight at 4 °C with gentle agitation. Following TBS-T washes, membranes were incubated with HRP-conjugated secondary antibodies diluted in 5% BSA for 1 h at room temperature. Blots were developed using Clarity Western ECL substrate (Bio-Rad, 1705061), imaged on a ChemiDoc MP imaging system (Bio-Rad), and quantified using Image Lab software.

Primary antibodies were used as follows: human GPNMB (Cell Signaling Technology, E4D7P, 38313; 1:1,000), mouse GPNMB (Abcam, EPR18226-147, ab188222; 1:1,000), β-actin (Cell Signaling Technology, 13E5, 4970; 1:5,000) and GAPDH (Cell Signaling Technology, 14C10, 2118; 1:1,000). Secondary antibodies were goat anti-mouse IgG (H+L)–HRP (Bio-Rad, 1706516; 1:3,000) and goat anti-rabbit IgG (H+L)-HRP (Bio-Rad, 1706515; 1:10,000).

### Generation of CAR-T cells

CAR constructs were packaged into lentiviral vectors as described above. Viral supernatants were collected 48 h post-transfection, concentrated by ultracentrifugation at 20,000 rpm for 2 h at 4 °C, reconstituted in 50 μl of ImmunoCult-XF T Cell Expansion Medium (StemCell, 10981), and stored at −80 °C. Peripheral blood mononuclear cells (PBMCs) were isolated from consenting healthy donors and cultured in ImmunoCult-XF medium, activated with ImmunoCult Human CD3/CD28/CD2 T Cell Activator (25 μl ml^−1^; StemCell, 10970) in 96-well roundbottom plates. Sixteen hours following activation, T cells were transduced with 15 μl of CAR lentivirus. T cells were expanded in ImmunoCult-XF medium with 2.5 μg ml^−1^ recombinant human IL-2 (StemCell, 78036) in 24-well plates for 14 days post-activation before plating assays. Transduction efficiency was determined using flow cytometry for GFP expression 7 days post-transduction.

### CAR-T cytotoxicity assay

Ffluc-expressing target cells were plated at 1 × 10^4^ cells per well in 96-well plates containing 100 μl of NCC medium with D-firefly luciferin potassium salt (Revvity, 122799; 75μg ml^−1^). Effector T cells were added at various effector-to-target ratios and incubated at 37 °C for up to 48 h. Spontaneous lysis controls contained target cells without CAR-T cells, and maximal lysis controls were treated with 1% NP-40 (Thermo Fisher, 98379). Bioluminescence was measured periodically with a luminometer (FLUOstar Omega Fluorescence 566 Microplate Reader; BMG LABTECH) as relative luminescence units (RLU). Per cent viability was normalized to target cells cultured without effectors, and subtracted from 100% to attain percentage specific lysis.

### CAR-T activation assays

Effector cells were co-cultured with 2.5 × 10^5^ target cells at a 1:1 ratio for 24 h in a 24-well plate. T cells were stained with anti-human CD3 (PE-Cy7-conjugated; BD Biosciences, 563423, 1:20) and analysed for activation markers CD25 (BV421-conjugated anti-human CD25 antibody; clone M-A251; BioLegend, 356113, 1:20) and CD69 (APC-conjugated mouse anti-human CD69 antibody; BD Biosciences, 555533, 1:20) by flow cytometry as described above.

### CAR-T cytokine release assays

Effector cells were co-cultured with 2.5 × 10^5^ target cells at a 1:1 ratio for 24 h. Supernatants were collected and stored at −80 °C. The DuoSet ELISA human IFNγ (R&D Systems, DY285B) and human TNF (R&D Systems, DY210) kits were used for quantification of cytokines, according to the manufacturer’s descriptions.

### Generation of murinized anti-GPNMB CAR-T cells

Mouse T cell activation and gamma retroviral production has been described previously^66^. In brief, single-cell suspensions were generated from freshly isolated mouse spleens. Spleens were mechanically dissociated and filtered through a 70-µm mesh and red bloods cells lysed (BioLegend, 420301). Cells (3 × 10^6^ per ml) were stimulated with 0.1 µg ml^−1^ each of anti-mouse CD3 (BioLegend, 100339) and CD28 (BioLegend, 102115) in T cell medium (RPMI supplemented with 10% FBS, L-glutamine, HEPES, non-essential amino acids, sodium pyruvate, penicillin–streptomycin, β-mercaptoethanol and mouse IL-2 (BioLegend, 575402)) for 5–6 days, expanding T cells every 2 days.

Gamma-retroviruses were generated on Plat-E cells transfected with pCL-Eco (Addgene #12371) and retrovirus transfer vector pRV2011 containing Thy1.1 transduction marker and a 2nd generation CD28/CD3z mouse CAR with scFVs targeting either human HER2 or mGpnmb and a MYC tag. Viral supernatants were concentrated 36 h after transfection with 100-kDa centrifugal filters (Amicon) and added to activated T cells 24 h after T cell activation. Transduction efficiency was measured by flow cytometry using Thy1.1–APC-Fire (BioLegend, 202543) and AF647–MYC (BioLegend, 626809).

### CAR-T in vivo trials

Ffluc-expressing GBM4 or GBM8 cells were intracranially implanted in NSG mice as described above. For GBM–macrophage co-inoculation, mice were injected with 1 × 10^5^ GBM8-ffluc and 1 × 10^5^ IL-4/IL-10/TGFβ-conditioned U937 cells (described below) in NSG mice. GL261-ffluc were intracranially implanted into C57BL/6 mice. Engraftment was confirmed by positive IVIS signal 2–3 days post-surgery, at which point mice were randomized to control or treatment arms. Mice received intracranial doses of 10^6^ UTD or CAR-T cells in 5 μl of PBS through the same burr hole at the indicated time points. Mice were monitored weekly by IVIS until the last control mouse succumbed to disease.

### Macrophage culturing and polarization

U937 cells were a gift from A. Rullo and cultured in RPMI medium (Gibco, 11875093) supplemented with 10% Fetal Bovine Serum (Gibco, 10082147). U937 monocytes were primed with 60 ng ml^−1^ PMA (Millipore Sigma, P1585) for 72 h, then treated with, 100 ng ml^−1^ IFNγ (STEMCELL Technologies, 78020), 20 ng ml^−1^ IL-4 (STEMCELL, 78045.1), 20 ng ml^−1^ IL-10 (STEMCELL, 78024), 20 ng ml^−1^ TGFβ (Gibco, 100-21) or conditioned medium collected and filtered from GBM8 cells, for 72 h as indicated. CAR-T cell cytotoxicity assays were performed as described above, with cells incubated in equal parts basal RPMI and Immunocult-XF medium (STEMCELL Technologies, 100-0956).

### GSC–macrophage–T cell triple co-cultures

For triple co-cultures, iRFP-expressing GBM8 cells were co-cultured with U937 cells and UTD- or GPNMB CAR-T cells at a 1:1:1 ratio in a 24-well plate, in equal parts NCC, basal RPMI, and Immunocult-XF medium. Cells were collected after 24 h and analysed by flow cytometry as described above with mouse anti-CD45 (BV421-conjugated, BioLegend, 368521) and anti-CD3 (PE/Cy7-conjugated, BD Pharmingen, 563423) antibodies. Tumour cells were gated on iRFP positivity. T Cells were gated on CD45/CD3 dual positivity. U937 cells were gated on CD45 positivity and CD3 negativity. Live cells were quantified by 7-AAD dye negativity and were normalized to the UTD control.

### scRNA-seq analysis

Patient samples and mouse brain tissue were immediately snap-frozen in liquid nitrogen after collection and stored at −80 °C for sci-RNA-seq3-based single-nucleus RNA-seq processing. Sci-RNA-seq3 libraries were generated as previously described using three-level combinatorial indexing^67^. After barcodes and UMIs were extracted from the read1 of FASTQ files, rawFASTQ files were aligned to the hg19genome using STAR aligner (STAR v2.5.2b) or with the mouse genome (mm10) and GencodevM12 gene annotations. For bioinformatic analyses, including preprocessing, clustering and annotation were performed as described^17^.

### Automated multiplexed sequential immunofluorescence imaging

Automated hyperplex immunofluorescence staining and imaging was performed on formalin-fixed, paraffin-embedded or frozen sections using the COMET platform (Lunaphore Technologies). The multiplex panel included antibodies described in Supplementary Table 2. The 22-plex protocol was generated using the COMET Control Software, and reagents were loaded onto the COMET device to perform the seqIF protocol. All antibodies were validated using conventional IHC and/or immunofluorescence staining in conjunction with corresponding fluorophores and DAPI counterstain (Thermo Fisher Scientific). For optimal concentration and best signal-to-noise ratio, all antibodies were tested at 3 different dilutions, starting with the manufacturer-recommended dilution (MRD), MRD/2 and MRD/4. Secondary Alexa fluorophore 555 (Thermo Fisher Scientific) and Alexa fluorophore 647 (Thermo Fisher Scientific) were used at 1/200 and 1/400 dilutions, respectively. The optimizations and full runs of the multiplexed panel were executed using the seqIF technology integrated in the Lunaphore COMET platform (characterization 2 and 3 protocols, and seqIF protocols, respectively). The seqIF workflow was performed parallelized on a maximum of four simultaneous slides, with automated iterative cycles of two targets with primary and secondary antibodies’ staining at a TME, followed by imaging, and elution of the primary and secondary antibodies. No sample manipulation is required during the entire workflow. All reagents were diluted in Multistaining Buffer (BU06, Lunaphore Technologies). Elution step lasted 2 min for each cycle and was performed with Elution Buffer (BU07-L, Lunaphore Technologies) at 37 °C. Quenching step lasted for 30 sec and was performed with Quenching Buffer (BU08-L, Lunaphore Technologies). Incubation TME was set at 4 min for all primary antibodies and at 2 min for secondary antibodies. The imaging step was performed with Imaging Buffer (BU09, Lunaphore Technologies) with an integrated epifluorescence microscope at 20× magnification. Image registration was performed immediately after concluding the immunofluorescence staining and imaging procedures by COMET Control Software. Each seqIF protocol resulted in a multi-layer OME-TIFF file in which the imaging outputs from each cycle are stitched and aligned. COMET OME-TIFF files contain DAPI image, intrinsic tissue autofluorescence in TRITC and Cy5 channels, and a single fluorescent layer per marker. Markers were subsequently pseudocoloured for visualization of multiplexed antibodies using the HORIZON Viewer software. Spatial bioinformatic analysis and quantification were then conducted using the learning algorithm and phenoplex feature of the Visiopharm Software.

### Reporting summary

Further information on research design is available in the Nature Portfolio Reporting Summary linked to this article.

## Online content

Any methods, additional references, Nature Portfolio reporting summaries, source data, extended data, supplementary information, acknowledgements, peer review information; details of author contributions and competing interests; and statements of data and code availability are available at 10.1038/s41586-026-10641-1.

## Supplementary information

Supplementary Figure 1 Unprocessed western blots related to Fig. 2a,d,g and Extended Data Fig. 7f.

Reporting Summary

Supplementary Table 1 Clinical and mutational analysis of patient tumour models.

Supplementary Table 2 List of antibodies used in seqIF imaging.

Peer Review File

## Source data

Source Data Figs. 1–5 and Extended Data Figs. 1–7 and 11

## Data Availability

Raw data for single-cell RNA experiments have been deposited as follows: primary and recurrent, 10.6084/m9.figshare.25917628.v1 (ref. ^68^); and *Gpnmb* data, 10.6084/m9.figshare.27643794.v1 (ref. ^69^). Raw data for all samples used in mass spectrometry have been deposited to the Mass Spectrometry Interactive Virtual Environment (MassIVE) with ID MSV000087947. NanoString data are also available on the Gene Expression Omnibus under accession code GSE177549. Public scRNA-seq data used the following: GSE182109 (ref. ^18^), https://cbl-dev.cells.ucsc.edu (ref. ^70^), SCR_002001(ref. ^71^), GSE185386 (ref. ^72^), GSE156793 (ref. ^67^), GSE186538 (ref. ^73^), GSE203552 (ref. ^74^), GSE118257 (ref. ^75^), E-MTAB-8230 (ref. ^76^), GSE131907 (ref. ^77^), GSE127774 (ref. ^78^), GSE157827 (ref. ^79^), https://cells.ucsc.edu/?ds=ms# (ref. ^80^), GSE157783 (ref. ^81^), GSE202371 (ref. ^82^), GSE131928 (ref. ^83^) and GSE165388 (ref. ^84^). Source data are provided with this paper.

## Code availability

All analysis code is available at GitHub (https://github.com/NMikolajewicz/Savage-2026).
