# Supplementary material for: Dual tumour–myeloid targeting of glioblastoma with GPNMB CAR-T cells
Source: Nature. 2026 Jul 1;656(8129):1013–22. doi: 10.1038/s41586-026-10641-1 (PMC13518245; doi:10.1038/s41586-026-10641-1)
Supplement: Supplementary file 1 [file 41586_2026_10641_MOESM1_ESM.pdf]

nature portfolio

https://doi.org/10.1038/s41586-026-10641-1

Supplementary information

Dual tumour–myeloid targeting of glioblastoma with GPNMB CAR-T cells

In the format provided by the
authors and unedited

Related to Figure 2a

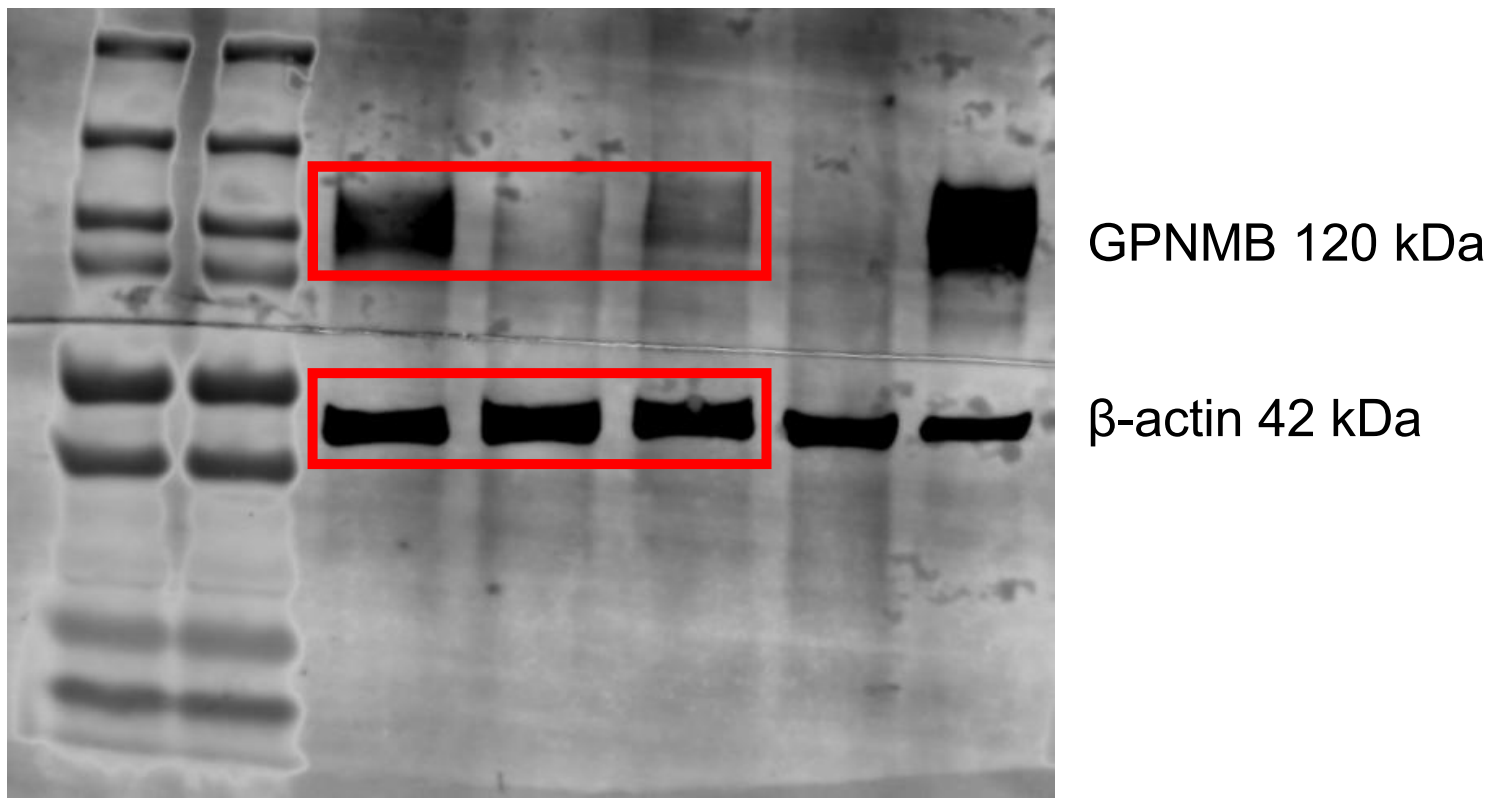

Related to Figure 2d

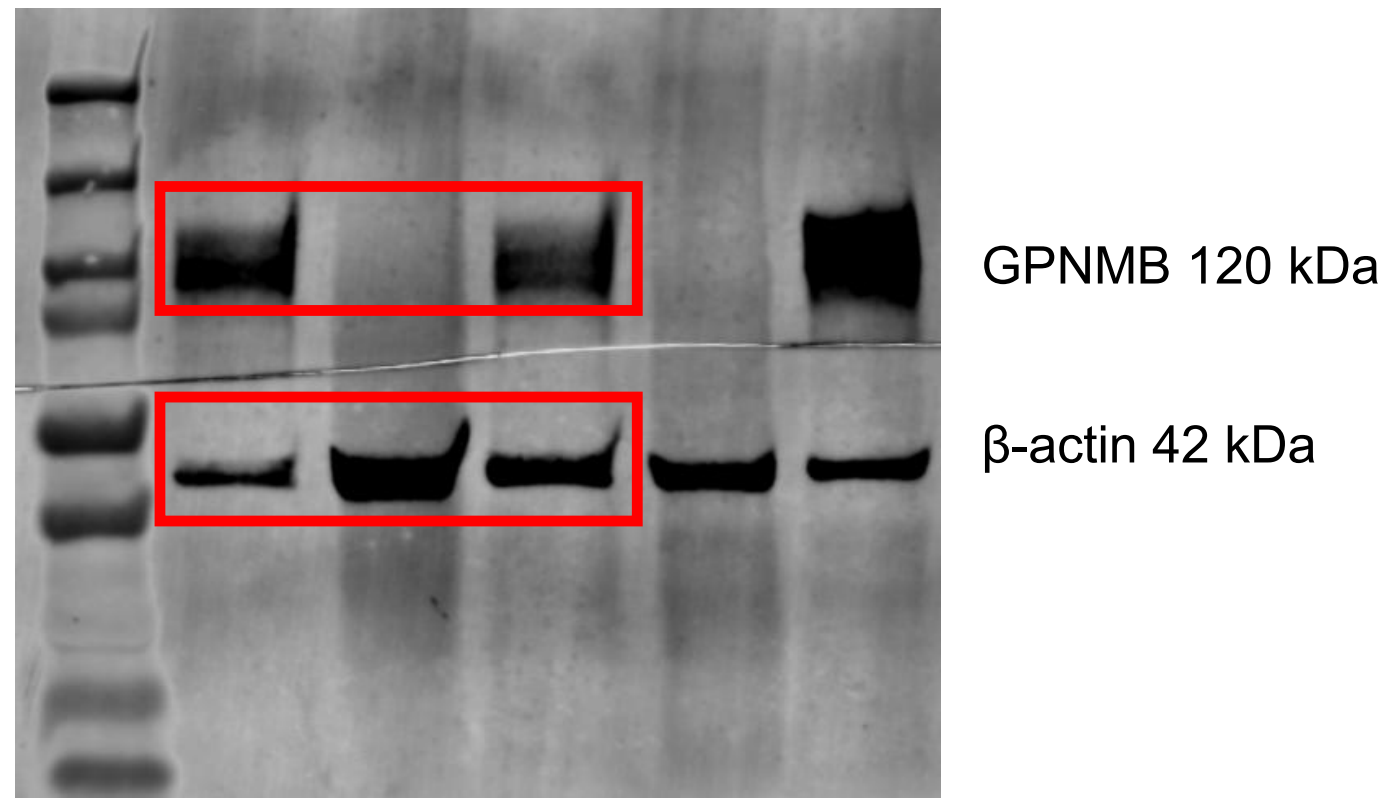

Related to Figure 2g

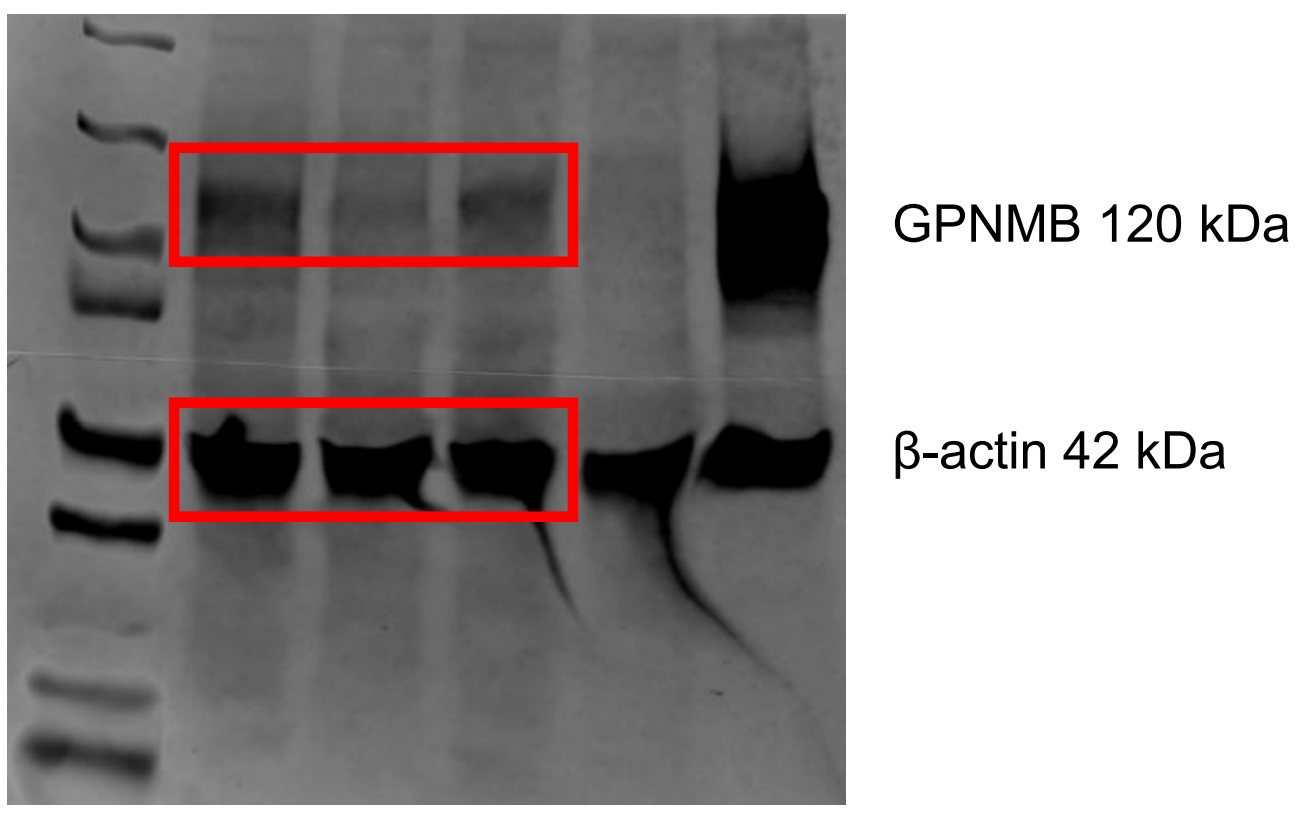

Related to Extended Data Fig 7F

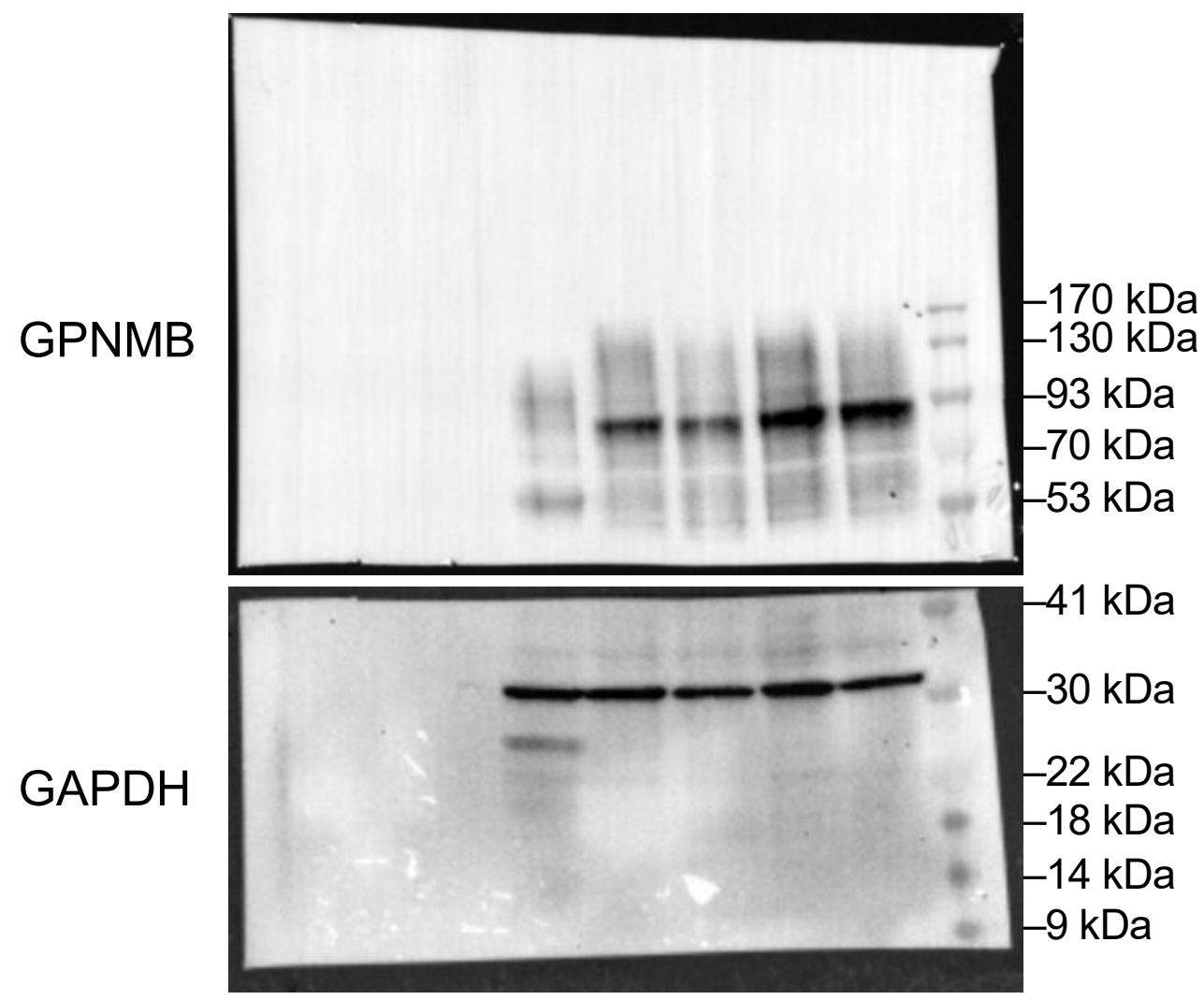

Supplementary Figure 1. Western blot source images.
