# Supplementary material for: Dual tumour–myeloid targeting of glioblastoma with GPNMB CAR-T cells
Source: Nature. 2026 Jul 1;656(8129):1013–22. doi: 10.1038/s41586-026-10641-1 (PMC13518245; doi:10.1038/s41586-026-10641-1)
Supplement: Supplementary file 2 [file 41586_2026_10641_MOESM2_ESM.pdf]

Corresponding author(s): Sheila Singh
Last updated by author(s): Apr 2, 2026

Reporting Summary

Nature Portfolio wishes to improve the reproducibility of the work that we publish. This form provides structure for consistency and transparency in reporting. For further information on Nature Portfolio policies, see our Editorial Policies and the Editorial Policy Checklist.

Statistics

For all statistical analyses, confirm that the following items are present in the figure legend, table legend, main text, or Methods section.

n/a	Confirmed	
☐	☒	The exact sample size (*n*) for each experimental group/condition, given as a discrete number and unit of measurement
☐	☒	A statement on whether measurements were taken from distinct samples or whether the same sample was measured repeatedly
☐	☒	The statistical test(s) used AND whether they are one- or two-sided<br>*Only common tests should be described solely by name; describe more complex techniques in the Methods section.*
☒	☐	A description of all covariates tested
☐	☒	A description of any assumptions or corrections, such as tests of normality and adjustment for multiple comparisons
☐	☒	A full description of the statistical parameters including central tendency (e.g. means) or other basic estimates (e.g. regression coefficient) AND variation (e.g. standard deviation) or associated estimates of uncertainty (e.g. confidence intervals)
☐	☒	For null hypothesis testing, the test statistic (e.g. *F*, *t*, *r*) with confidence intervals, effect sizes, degrees of freedom and *P* value noted<br>*Give P values as exact values whenever suitable.*
☒	☐	For Bayesian analysis, information on the choice of priors and Markov chain Monte Carlo settings
☐	☒	For hierarchical and complex designs, identification of the appropriate level for tests and full reporting of outcomes
☐	☒	Estimates of effect sizes (e.g. Cohen's *d*, Pearson's *r*), indicating how they were calculated

Our web collection on statistics for biologists contains articles on many of the points above.

Software and code

Policy information about availability of computer code

Data collection	Bulk RNAseq was collected using Illumina HiSeq 2500 platform. Single-cell RNA was collected using Illumina NovaSeq platform. Whole cell proteomic data was collected using Orbitrap Fusion Mass Spectrometer (Thermo Fisher Scientific). Flow cytometry data was collected from CytoFLEX (Beckman Coulter) and MoFlo (Beckman Coulter).
Data analysis	All software and related packages are stated in the manuscript and below:<br>R version 4.0.4<br>fgsea (v1.14.0)<br>Enrichment Map (v3.3.0)<br>AutoAnnotate (v1.3.3)<br>STAR short-read aligner (STAR v.2.5.2b)<br>limma (v3.44.3)<br>edgeR (v3.30.3)<br>Genome Analysis Toolkit (GATK) version 3.1<br>UniProt human protein database (Ver 2017-06, 42,173 entries)<br>Perseus software (only one version available and no version number available)<br>FlowJo 10.10.0 (Becton Dickinson and company)<br>ZEISS ZEN Lite (v3.13)

For manuscripts utilizing custom algorithms or software that are central to the research but not yet described in published literature, software must be made available to editors and reviewers. We strongly encourage code deposition in a community repository (e.g. GitHub). See the Nature Portfolio guidelines for submitting code & software for further information.

Data

Policy information about availability of data

All manuscripts must include a data availability statement. This statement should provide the following information, where applicable:

- Accession codes, unique identifiers, or web links for publicly available datasets
- A description of any restrictions on data availability
- For clinical datasets or third party data, please ensure that the statement adheres to our policy

Raw data for single-cell RNA experiments have been deposited to the following: Primary/recurrent: snRNA-seq, Primary-Recurrent GBM (Mikolajewicz Cohort). figshare. Dataset. https://doi.org/10.6084/m9.figshare.25917628.v1. Gpnmb data: nRNA-seq, WT vs. Gpnmb-perturbed GL261 tumors (Savage et al. 2025). figshare. Dataset. https://doi.org/10.6084/m9.figshare.27643794.v1 Raw data for all samples used in mass spectrometry have been deposited to the Mass Spectrometry Interactive Virtual Environment (MassIVE) with the following MassiVE ID: MSV000087947 and FTP link: ftp://massive.ucsd.edu/MSV000087947/. NanoString data is also available on GEO with the accession ID: GSE177549.
Public scRNA-seq data: Abdelfattah et al. was retrieved from Gene Expression Omnibus (GEO: GSE182109) Aldinger et al. from UCSC Cell Browser (https://cbl-dev.cells.ucsc.edu); Bhaduri et al. from NEMO Archive (RRID:SCR_002001); Biermann et al. from GEO (GEO: GSE185386); Cao et al. from GEO (GEO: GSE156793); Franjic et al. from GEO (GEO: GSE186538); Heming et al. from GEO (GEO: GSE203552); Jakel et al. from GEO (GEO: GSE118257); Kanton et al. from ArrayExpress (E-MTAB-8230); Kim et al. from GEO (GEO: GSE131907); Khrameeva et al. from GEO (GEO: GSE127774); Lau et al. from GEO (GEO: GSE157827); Schirmer et al. from UCSC Cell Browser (https://cells.ucsc.edu/?ds=ms#); Smajic et al. from GEO (GEO: GSE157783); Sun et al. from GEO (GEO: GSE202371); Wang et al. from GEO (GEO: GSE131928); Yu et al. from GEO (GEO: GSE165388).

Research involving human participants, their data, or biological material

Policy information about studies with human participants or human data. See also policy information about sex, gender (identity/presentation), and sexual orientation and race, ethnicity and racism.

Reporting on sex and gender	Both male and female patient-derived samples were used in this study for all experiments presented in this manuscript. All the human datasets report findings from both sexes.
Reporting on race, ethnicity, or other socially relevant groupings	Not applicable to this study.
Population characteristics	The patient samples used were from people diagnosed with primary or recurrent GBM. Patient demographics are listed in Supplementary Table 1.
Recruitment	Brain tumor samples were collected from patients only after receiving written consent and in accordance with relevant ethical regulation.
Ethics oversight	Glioblastoma (GBM) specimens and whole fetal brain samples were obtained from consenting patients and families as approved by the Hamilton Health Sciences and McMaster Health Sciences Research Ethics Board. Other lines mentioned was obtained through an MTA.

Note that full information on the approval of the study protocol must also be provided in the manuscript.

Field-specific reporting

Please select the one below that is the best fit for your research. If you are not sure, read the appropriate sections before making your selection.

☒ Life sciences ☐ Behavioural & social sciences ☐ Ecological, evolutionary & environmental sciences

For a reference copy of the document with all sections, see nature.com/documents/nr-reporting-summary-flat.pdf

Life sciences study design

All studies must disclose on these points even when the disclosure is negative.

Sample size	In vivo sample sizes were chosen for in vivo studies based on prior studies which validated the minimum number of mice to determine a significant difference (Gwynne et al., 2023 Cancer Cell PMID 36368321; Vora et al., 2020 Cell Stem Cell PMID 32464096). This was determined at a minimum of 5 mice per treatment arm. In vitro sample sizes were selected based on the number of available biologically independent primary cell lines and established field standards. Each experiment was performed with multiple biological replicates (distinct cell lines where applicable) and repeated independently at least three times. These sample sizes were sufficient to ensure consistent effect sizes and reproducibility across experiments.
Data exclusions	No data was excluded in this study.
Replication	All attempts at data replication were taken to confirm previous results. Unless otherwise stated, each experiment was repeated independently at least 3 times with similar results.

Randomization	Randomization was not relevant to in vitro studies as they did not involve allocation of subjects or samples into treatment groups in a manner that could introduce selection bias. Cells from the same population were plated and exposed to experimental conditions in parallel under standardized culture conditions.<br><br>Mouse studies were divided as evenly as possible while assuring the mean of control tumors were smaller than treatments to assure no bias that efficacy was to be attributed to smaller starting tumor volumes. Prior to CAR-T injections in vivo mouse tumor volumes were measured and ranked smallest to largest volume. Smallest volume was designated to control treatments, with second smallest designated to treatment and alternated from then on until all mice had been allocated.
Blinding	Investigators were blinded to group allocation. Data was combined after complete data collection and processing, allowing for unbiased analyses.

Reporting for specific materials, systems and methods

We require information from authors about some types of materials, experimental systems and methods used in many studies. Here, indicate whether each material, system or method listed is relevant to your study. If you are not sure if a list item applies to your research, read the appropriate section before selecting a response.

Materials & experimental systems

n/a	Involved in the study	
☐	☒	Antibodies
☐	☒	Eukaryotic cell lines
☒	☐	Palaeontology and archaeology
☐	☒	Animals and other organisms
☒	☐	Clinical data
☒	☐	Dual use research of concern
☒	☐	Plants

Methods

n/a	Involved in the study	
☒	☐	ChIP-seq
☐	☒	Flow cytometry
☒	☐	MRI-based neuroimaging

Antibodies

Antibodies used

Western Blot
Human GPNMB (Cell Signaling Technologies, E4D7P Cat.38313)
Human GPNMB (R&D Systems, AF2550)
Mouse GPNMB (Abcam, EPR18226-147, Cat.ab188222)
Beta-Actin (Cell Signaling Technologies, Clone 13E5, Cat.4970)
GAPDH (Cell Signaling Technologies, Clone 14C10, Cat.2118)
anti-Rabbit IgG Secondary (LICOR, 926-32211)
Donkey anti-Goat IgG (H+L) Secondary Antibody, HRP (Invitrogen Cat. A15999)
Goat anti-mouse IgG (H+L)-HRP (Bio-Rad, Cat. 1706516)
Goat anti-rabbit IgG (H+L)-HRP (Bio-Rad, Cat. 1706515)

Flow Cytometry
Human GPNMB PE (Invitrogen Clone HOST5DS, Cat.17-9838-42)
Mouse GPNMB eFluor660 (Invitrogen Clone CTSREVL, Cat.50-5708-80)
CD3 PE-Cy7 (BD Biosciences, Cat.563423)
CD25 BV421 (BioLegend, M-A251, Cat. 356113)
CD69 APC (BD Biosciences, Cat.555533)
Mouse IgG CompBeads (BD Biosciences, Cat#552843)
7AAD viability dye (Beckman Coulter Cat.A07704)

ELISA
IFN-gamma (R&D Systems, Cat.DY285B)
TNF-alpha (R&D Systems, Cat.DY210)

Immunohistochemistry
Human GPNMB (Cell Signaling Technologies, E4D7P, Cat.38313)
Human CD133 (Cell Signaling Technologies, D2V8Q, Cat.64326T)
Donkey Anti-Rabbit IgG HRP (abcam, Cat.ab205722)
Donkey Anti-Goat IgG HRP (Abcam, Cat. ab214881)

seqIF autmated multiplexed imaging
GFAP (Sigma Clone GA5 MAB360)
CD31 (Abcam Clone EPR17259 Ab225883)
CD3 (Dako Agilent Clone F7.2.38 M7254)
CD4 (Abcam Clone EPR19514 Ab183685)
CD8 (Cell signaling Clone D4W2Z XP® 98941)
CD11c (Cell signalingClone D1V9Y 97585)
CD68 (Santa Cruz Clone KP1 Sc-20060)

April 2023

	CD163 (Abcam Clone EPR19518 Ab182422)<br>FOXP3 (Cell signaling tech Clone D2W8E 98377S)<br>IFN-γ (Bioss BS-0480R Polyclonal)<br>MHC II (Abcam MRC OX-6 Ab23990)<br>Granzyme B (Santa Cruz Clone 2C5 Sc-8022)<br>CD45RO (Santa Cruz Clone UCH-L1 Sc-1183)<br>iNOS (Santa Cruz Clone C-11 Sc-7271)<br>CD206 (Abcam Polyclonal Ab64693)<br>GPNMB (ProteinTech 66926-1-Ig Clone 2B10B8)<br>c-Caspase 3 (Cell signaling Asp175 9661S)<br>TNF-α (Abcam Clone 52B83 Ab1793)<br>CD64 (ThermoFisher MA5-29706 Clone 027)<br>GFP (Abcam Ab6556 Polyclonal)<br>AF647 (ThermoFisher Scientific A32733)<br>AF555 (ThermoFisher Scientific A32727)<br>DAPI (ThermoFisher Scientific 62248)<br><br>Immunofluorescence<br>Human GPNMB (R&D AF2550)<br>Mouse GPNMB (R&D AF2330)<br>IBA1 (Abcam EPR16588)<br>DAPI (Abcam Cat. ab104139)<br>Anti-rabbit AF488 (Intvitrogen Cat. A-21206)<br>Anti-goat AF594 (Invitrogen Cat. A-11058)<br>Anti-goat AF647 (Invitrogen Cat. A-21469)
Validation	All above mentioned antibodies were purchased from commercial vendors and were validated by the manufacturer and used in accordance to manufacturer's guidelines or previous publications.

Eukaryotic cell lines

Policy information about cell lines and Sex and Gender in Research

Cell line source(s)	Human adult glioblastoma (GBM) lines were generated internally from patient tumor specimens and were obtained from consenting patients and families as approved by the Hamilton Health Sciences and McMaster Health Sciences Research Ethics Board. These cell lines include Glioblastoma: BT241, BT428, BT555, BT594, BT799, BT935, BT972, MBT06, MBT63, MBT289, MBT298, MBT572; Neural stem cells (NSC201).GBM4, GBM8, GBM87 were a gift from Dr. Hiroaki Wakimoto (Massachusetts General Hospital, Boston, MA, USA). SK-MEL-2, MDA-MB-231, HEK293T cells, and normal human astrocytes (NHA) purchased from the American Type Culture Collection (ATCC).
Authentication	Clinical pathology report and STR profiling was performed on all cell cultures.
Mycoplasma contamination	All cell lines were grown in mycozap prior to experiments and tested frequently for mycoplasma contamination, which tested negative.
Commonly misidentified lines<br>(See ICLAC register)	No commonly misidentified cell lines were used in this study.

Animals and other research organisms

Policy information about studies involving animals; ARRIVE guidelines recommended for reporting animal research, and Sex and Gender in Research

Laboratory animals	Male and female laboratory NSG and C57BL/6 mice of at least 6-12 weeks of age were used. Humanized NOG-EXL mice (6-12 weeks) were all female. Mice were maintained in the animal facilities at the McMaster University Stem Cell Unit (SCU) within the Animal Facility (CAF). They were maintained in a pathogen-free, temperature-controlled, 12h light and dark cycle environment and were fed ad libitum
Wild animals	No wild animals were used in this study.
Reporting on sex	Both male and female mice were used for all in vivo experiments.
Field-collected samples	No field-collected samples were used in this study.
Ethics oversight	All in vivo experiments were performed in accordance to the McMaster University Animal Research Ethics Board (AREB) approved protocols national guidelines and regulations.

Note that full information on the approval of the study protocol must also be provided in the manuscript.

Plants

Seed stocks	*Report on the source of all seed stocks or other plant material used. If applicable, state the seed stock centre and catalogue number. If plant specimens were collected from the field, describe the collection location, date and sampling procedures.*
Novel plant genotypes	*Describe the methods by which all novel plant genotypes were produced. This includes those generated by transgenic approaches, gene editing, chemical/radiation-based mutagenesis and hybridization. For transgenic lines, describe the transformation method, the number of independent lines analyzed and the generation upon which experiments were performed. For gene-edited lines, describe the editor used, the endogenous sequence targeted for editing, the targeting guide RNA sequence (if applicable) and how the editor was applied.*
Authentication	*Describe any authentication procedures for each seed stock used or novel genotype generated. Describe any experiments used to assess the effect of a mutation and, where applicable, how potential secondary effects (e.g. second site T-DNA insertions, mosiacism, off-target gene editing) were examined.*

Flow Cytometry

Plots

Confirm that:

☒ The axis labels state the marker and fluorochrome used (e.g. CD4-FITC).

☒ The axis scales are clearly visible. Include numbers along axes only for bottom left plot of group (a 'group' is an analysis of identical markers).

☒ All plots are contour plots with outliers or pseudocolor plots.

☒ A numerical value for number of cells or percentage (with statistics) is provided.

Methodology

Sample preparation	Cells were dissociated with TrypLE and suspended in PBS+0.5M EDTA to achieve a single cell suspension with subsequent filtration to remove clumps.
Instrument	CytoFlex LX (Beckman Coulter)
Software	Flow Jo v10.10
Cell population abundance	A small fraction of sorted populations were re-run through the MoFlo XDP to check for purity using the same gating strategy. Purity is determined by the percentage of cells that falls under the same gating used for sort. If purity is greater than 95%, sample was considered pure.
Gating strategy	GPNMB flow analysis: (a) FSC-Height vs. SSC-Height (b) Viability gate is set using 7-AAD viability dye to exclude non-viable cells (c) Unstained controls were used to set the gate for expression of GPNMB to exclude baseline expression of fluorophores.

☒ Tick this box to confirm that a figure exemplifying the gating strategy is provided in the Supplementary Information.
