# Supplementary material for: Dual tumour–myeloid targeting of glioblastoma with GPNMB CAR-T cells
Source: Nature. 2026 Jul 1;656(8129):1013–22. doi: 10.1038/s41586-026-10641-1 (PMC13518245; doi:10.1038/s41586-026-10641-1)
Supplement: Supplementary file 3 [file 41586_2026_10641_MOESM3_ESM.pdf]

Table S1. Clinical and mutational analysis of tumor models.

Sample	Age/Sex	Diagnosis	Survival (months)	*PTEN* variants	*TP53* expression	*TP53* variants	IDH status
BT241	69/F	rGBM	23	VL317-318X	+	R280K, P72R	WT
BT428	63/F	pGBM	NA	NA	NA	NA	WT
BT555	65/M	pGBM	NA	NA	NA	NA	WT
BT594	50/M	pGBM	41	WT	+	WT	WT
BT799	77/F	pGBM	NA	NA	NA	NA	WT
BT935	53/F	pGBM	7	F195S	+	E286K, P72R	WT
BT972	53/M	rGBM	41	H123Y	+	P190L, G244D	WT
MBT06	53/F	pGBM	54	NA	–	NA	WT
MBT63	68/F	pGBM	15	NA	+	NA	WT
MBT289	47/F	rGBM	20	NA	–	NA	WT
MBT298	75/F	rGBM	9	NA	+	NA	WT
MBT572	60/F	rGBM	NA	NA	+	NA	WT
GBM4<br>GBM8<br>GBM87	Generous gift from Dr. Hiroaki Wakimoto						
