# Supplementary material for: Dual tumour–myeloid targeting of glioblastoma with GPNMB CAR-T cells
Source: Nature. 2026 Jul 1;656(8129):1013–22. doi: 10.1038/s41586-026-10641-1 (PMC13518245; doi:10.1038/s41586-026-10641-1)
Supplement: Supplementary file 4 [file 41586_2026_10641_MOESM4_ESM.pdf]

Table S2. Antibodies used in seqIF imaging

Marker	Antibody information/Dilution
GFAP	Sigma, Clone GA5, cat# MAB360, dilution 1/3000
CD31	Abcam, Clone EPR17259, Ab225883, 1/1500
CD3	Dako Agilent, Clone F7.2.38, M7254, 1/50
CD4	Abcam, Clone EPR19514, Ab183685, 1/500
CD8	Cell signaling, Clone D4W2Z XP®, 98941, 1/500
CD11c	Cell signaling, Clone D1V9Y, 97585, 1/300
CD68	Santa Cruz, Clone KP1, Sc-20060, 1/200
CD163	Abcam, Clone EPR19518, Ab182422, 1/600
FOXP3	Cell signaling, Clone D2W8E, 98377S, 1/100
IFNγ	Bioss, Polyclonal, BS-0480R, 1/100
MHC II	Abcam, Clone MRC OX-6, Ab23990, 1/500
Granzyme B	Santa Cruz, Clone 2C5, Sc-8022, 1/500
CD45RO	Santa Cruz, Clone UCH-L1, Sc-1183, 1/100
iNOS	Santa Cruz, Clone C-11, Sc-7271, 1/100
CD206	Abcam, Polyclonal, Ab64693, 1/1000
GPNMB	ProteinTech, Clone 2B10B8, 66926-1-Ig, 1/500
c-Caspase 3	Cell signaling, Clone Asp175, 9661S, 1/250
TNFα	Abcam, Clone 52B83, Ab1793, 1/100
CD64	ThermoFisher, Clone 027, MA5-29706, 1/100
GFP	Abcam, Ab6556, Polyclonal, 1/100
AF647	ThermoFisher Scientific, A32733, 1/400
AF555	ThermoFisher Scientific, A32727, 1/200
DAPI	ThermoFisher Scientific, 62248, 1/1500
