# Supplementary material for: Dual tumour–myeloid targeting of glioblastoma with GPNMB CAR-T cells
Source: Nature. 2026 Jul 1;656(8129):1013–22. doi: 10.1038/s41586-026-10641-1 (PMC13518245; doi:10.1038/s41586-026-10641-1)
Supplement: Supplementary file 5 [file 41586_2026_10641_MOESM5_ESM.pdf]

nature portfolio

Peer Review File

Dual tumor-myeloid targeting of glioblastoma with GPNMB CAR-T cells

Corresponding Author: Dr Sheila Singh

Any redactions in this file are there to maintain patient confidentiality, the confidentiality of unpublished data, or to remove third-party material.

This file contains all reviewer reports in order by version, followed by all author rebuttals in order by version.

Version 0:

Reviewer comments:

Referee #1

(Remarks to the Author)
In this manuscript (2024-12-26538), Savage et al. leverage patient glioblastoma (GBM) samples to identify GPNMB as a novel therapeutic target for GBM cells as well as tumor associated macrophages (TAMs). Using this information, the authors engineer chimeric antigen receptor (CAR) T cells, which show efficacy against each population (GBM cells and TAMs) with CAR T cells again CD133. In general, this is an exciting set of findings that shows a novel target against both GBM cells and TAMs, which can drive GBM progression through immune suppression. This is also a great example of using unbiased analysis of human samples to find novel therapeutic targets and then develop CARs against them. While my enthusiasm for this work is quite high, there are some technical issues that need to be resolved/improved to ensure the conclusions made a robustly supported by the data provided.

1. The use of the GL261 model is not optimal in terms of its ability to recapitulate the cellular and molecular complexity of GBM, as well as the TIME and overall immune response. The studies using human models are well done and it would be nice to match these with more representative mouse models. Validation in a more representative syngeneic mouse model and/or GEMM model would be useful here.

2. The authors do an exceptional job of validating GPNMB expression in GBM cells and TAMs, both using RNA and protein data and many relevant controls. However, the cell surface expression and antibodies studies (Extended Data Fig 3) are much less robust. It would be nice to see some additional models (mouse/human GBM) and relevant controls (astrocytes, NSCs, other immune lineages) to show cell surface expression via flow cytometry.

3. The claims in GPNMB targeting impacting both GBM cells and TAMs could be further strengthened by doing some studies with the CAR T cells in the context of TAM depletion (humanized mouse model would be an obvious choice, but in an immune competent mouse model would be fine as well). This would help show the extent to which survival is impacted by TAM targeting via GPNMB.

4. I am not sure the GSC terminology is needed earlier in the paper and the focus could just be on targets in CD133- populations. This could be framed in the context of previous work from the Singh lab marking CAR T cells against CD133+ cells and then supporting the need to find targets for the CD133- population. In line with this, I am a bit confused by the data in Extended Data Fig 5b as GPNMB was identified as the top hit in CD133- cells but these data show it is present in a subset of CD133+ cells. This would be worth a brief explanation.

Referee #2

(Remarks to the Author)
In the submitted manuscript, the authors report on the role on GPNMB in GBM, demonstrate that it is expressed in TAMs, develop GPNMB-CARTs to deplete TAMs, and perform in vitro as well as in vivo studies. While the studies are well conducted and interesting, there are significant concerns.

Major concerns:
1) Limited conceptual novelty: other investigators have published on the role of GPNMB+ macrophages in promoting tumor progression including for GBM (e.g., https://doi.org/10.1186/s40478-024-01754-7). Likewise, targeting TAMs with CARTs has been explored by others (e.g., https://doi.org/10.1158/2326-6066.CIR-21-1075).

2) Limited novelty regarding T cell engineering: no novel CAR design or genetic engineering approach of CARTs is explored.

3) Tumour models: the anti-tumour activity of GPNMB-CARTs as a single agent or in combination with CD133-CARTs is limited. This raises significant concerns. It appears that CARTs derived from only one donor were evaluated. Please provide data for at least two independent donors.

4) The authors use immunodeficient xenograft models to study the immunosuppressive tumor microenvironment and the effects of CARTs on tumour associated macrophages. The tumour microenvironment in immunodeficient xenograft models is not representative of human tumours and it is critical to conduct studies in immune competent animal models.

Minor concerns:
1) The construction of the CAR is not described in the material and methods section.

2) Limited information is provided on the generated CART products (e.g., efficiency, T cell phenotype, etc).

3) Fig. 3e,f: it is stated that experimental replicates are shown. Please show data for at least three different donors.

4) It appears that CAR expression is not directly demonstrated and only one example is shown (Extended Figure 3). Please show directly that the CAR is expressed and show data for multiple donors.

Referee #3

(Remarks to the Author)
The authors explore GBPNMB as a potential target in glioblastoma because it is expressed in GBM and TAMs using various datasets and also a series of biobanked specimens. However, GPNMB has already been identified as a promising target in various cancers, including GBM, as per the referenced literature in the paper. There have also already been trials of antibodies targeting GPNMB (glembatumumab vedotin), which have been unsuccessful. The authors here generate CARs targeting GPNMB using the same binding regions as the previously tested antibody. They also hypothesize that because of existing literature indicating that tumor-associated macrophages express GPNMB, that the CAR-T cells would target both the GBM and its associated TAMs, and therefore a single CAR would function as a combination strategy. However, the manuscript has modest novelty since nowadays building a CAR to an established antigen, using binding domains from an established antibody, and testing it in vitro and in vivo is fairly routine. Furthermore, the anti-tumor effects in the in vivo experiments was quite modest as well.

I have several concerns.
1. GPNMB is thought to play a critical role in the survival of motor neurons, and is thought to potentially be associated with ALS. The authors should consider whether targeting this molecule could result in death of motor neurons, which could be a severe toxicity. Furthermore, on-target toxicities toward bone and skin could be serious (and the skin toxicity was a dose-limiting toxicity of previous antibodies). Its confirmation here is not reassuring. The tissue microarray should also include motor neurons.
2. The biologic significance of GPNMB being upregulated in recurrent as opposed to primary GBM is not clear. Both kinds of GBM are highly proliferative, so the role of GPNMB as being important for proliferation of GBM is important but not necessarily connected?
3. Is there a knockout mouse of GPNMB?
4. In Figure 1, what is the level of expression GPNMB in GBM relative to melanoma and breast cancer, where GV has already been tested?
5. In Figure 3, the GPNMB-CAR-T should also be tested against the GPNMB-KO glioma as target cells, since the authors already made it and would confirm the CAR's specificity.
6. Figure 4—although there is improved survival and some delay in tumor progression, it doesn't seem like either single or dual targeting of GPNMB +/- CD133 induces sustained responses.
7. The abbreviation of the "huNOG-EXL mouse model" is not a strain that is in frequent use in the US. Can you please use the more common strain name or describe what it it is transgenic for and what it is deficient in in the main text?
8. In Figure 5, are the T cells used autologous to the humanized mouse? Allogeneic to the tumor? Again, the degree of tumor control as shown in Fig 5a and 5b is quite modest.

Minor points:
1. Please don't invent a new acronym. "Tumor micronvironment" is often already abbreviated as "TME", it is confusing to call it "tumor immune microenvironment" and abbreviate it as "TIME."

2. Page 17 – “the feasibility of dual-targeting CAR-T cells with two single-domain antibodies has already been shown by the dramatic clinical success and commercial approval of cilta-cel.” This is a mistake. Cilta-cel is not dual-targeting of two different antigens, it is dual targeting of two epitopes of the same antigen (biparatopic).

Version 2:

Reviewer comments:

Referee #1

(Remarks to the Author)
In general, all previously raised issues have been addressed.

Referee #2

(Remarks to the Author)
I appreciate the authors’ efforts in submitting a revised manuscript that addresses several of the concerns raised previously. Nevertheless, substantial issues remain. Foremost among these is the question of conceptual novelty. As noted in my initial review, prior studies have already characterized the role of $GPNMB^{+}$ macrophages in promoting tumor progression, including in GBM (e.g., PMID: 38566120) as well as in other malignancies. Additionally, the biology of GPNMB within the tumor microenvironment has been extensively investigated, with several of these findings summarized in PMID: 36050467. Moreover, as previously mentioned, the strategy of targeting tumor-associated macrophages with CAR T cells has been explored by multiple groups of investigators (e.g., PMID: 36095236; PMID: 33563975). Taken together, these observations continue to raise significant concerns regarding the level of novelty and, consequently, the suitability of this manuscript for a journal with the broad and general readership of Nature.

Referee #3

(Remarks to the Author)
The authors have substantially improved the manuscript.

One note -- some of the responses to reviewers did not result in any changes to the manuscript -- given that reviewers do this in an effort to improve the manuscript, it would be nice if the authors incorporated more of the suggestions even if it didn't result in a new figure or experiment. Just one example --I asked about whether there was a GPNMB KO mice, and in the context of its phenotype as it could relate to safety -- the authors kindly replied that there was and that there are references to the knockout mice in the rebuttal, but these references are not included or commented on in the manuscript.

Version 4:

Reviewer comments:

Referee #1

(Remarks to the Author)
As I have previously contended, this work is completed and worthy of publication here at Nature. It is an elegant demonstration of the development of a dual CAR-T targeting strategy that has profound implications for glioblastoma and other solid tumors which have a high degree of local myeloid suppression.

Referee #3

(Remarks to the Author)
ok

Open Access This Peer Review File is licensed under a Creative Commons Attribution 4.0 International License, which permits use, sharing, adaptation, distribution and reproduction in any medium or format, as long as you give appropriate credit to the original author(s) and the source, provide a link to the Creative Commons license, and indicate if changes were made.
In cases where reviewers are anonymous, credit should be given to 'Anonymous Referee' and the source.
The images or other third party material in this Peer Review File are included in the article's Creative Commons license, unless indicated otherwise in a credit line to the material. If material is not included in the article's Creative Commons license and your intended use is not permitted by statutory regulation or exceeds the permitted use, you will need to obtain permission directly from the copyright holder.
To view a copy of this license, visit https://creativecommons.org/licenses/by/4.0/

We thank the Editor and Reviewers for their thorough feedback, which we have implemented to strengthen this work. In the revised submission, we position GBM as a coupled tumor-myeloid system and demonstrate GPNMB is a therapeutically actionable cell surface antigen that can be targeted with a single CAR-T to simultaneously debulk treatment-resistant malignant populations and remodel the immunosuppressive microenvironment, a new and relevant approach for other solid tumors. In response to the prior reviews, we have comprehensively reworked the entire manuscript over the past year and generated substantial new data. Key additions include 5 new *in vivo* studies showing (*i*) improved efficacy in PDXs using an optimized CAR-T manufacturing protocol, (*ii*) a revised CD133 co-targeting strategy timed to post-treatment recurrence, (*iii*) direct evidence of dual-compartment targeting using an intracranial GBM+TAM co-inoculation model in NSG mice, and (*iv*) validation in immunocompetent models through generation of a new murinized CAR-T targeting the mouse homolog of GPNMB. We further performed multiple *in vitro* studies requested by reviewers, including flow profiling GBM samples benchmarked against non-malignant CNS, melanoma, and breast cancer cell lines, specificity testing, and reproducibility across T cell donors. We hope the reviewers find these revisions comprehensive and satisfactory, and we are grateful for the feedback that has strengthened the manuscript through the review process. Given the overlap in comments, response data have been appended to the end of this document, and Figures are cited throughout.

Referee #1 (Remarks to the Author):

In this manuscript (2024-12-26538), Savage et al. leverage patient glioblastoma (GBM) samples to identify GPNMB as a novel therapeutic target for GBM cells as well as tumor associated macrophages (TAMs). Using this information, the authors engineer chimeric antigen receptor (CAR) T cells, which show efficacy against each population (GBM cells and TAMs) with CAR T cells again CD133. In general, this is an exciting set of findings that shows a novel target against both GBM cells and TAMs, which can drive GBM progression through immune suppression. This is also a great example of using unbiased analysis of human samples to find novel therapeutic targets and then develop CARs against them. While my enthusiasm for this work is quite high, there are some technical issues that need to be resolved/improved to ensure the conclusions made a robustly supported by the data provided.

1. The use of the GL261 model is not optimal in terms of its ability to recapitulate the cellular and molecular complexity of GBM, as well as the TIME and overall immune response. The studies using human models are well done and it would be nice to match these with more representative mouse models. Validation in a more representative syngeneic mouse model and/or GEMM model would be useful here.

Response: We thank the reviewer for raising this comment regarding model choice. In the revised manuscript, we substantially expanded our immunocompetent syngeneic in vivo testing (detailed in later responses). We agree chemically induced syngeneic glioma models do not fully recapitulate the genetic diversity and immune microenvironment of human GBM, but are nevertheless a useful and convenient model for immunocompetent testing. In our recent work,[1] we performed extensive characterization of these murine cell lines through *in vivo* and *in vitro* scRNA-seq (**Response Fig. 1A**) and genome-wide CRISPR screening. We found both GL261 and CT2A exhibit high transcriptomic similarity to WHO Grade IV GBM[2,3] (**Response Fig. 1B**) and retain multiple hallmark gene programs observed in human disease, including developmental, proliferative, and mesenchymal gene programs (**Response Fig. 1C**). Additionally, single-cell profiling of the tumor microenvironment shows both models are dominated by myeloid populations (**Response Fig. 1D**), and functional annotation of in vivo immune subsets identifies conserved

immune states also detectable across human GBM scRNA-seq datasets (**Response Fig. 1E-F**), suggesting the TME of murine gliomas can recapitulate hallmark features of the human immune response in GBM. In parallel, we explored somatically engineered glioma models generated by neonatal intracranial electroporation of Cdk4 and RAS_{V12} (piggyBac–mediated overexpression) with CRISPR-mediated Trp53 knockout. Tumors arising in these mice were cultured *ex vivo* and re-engrafted to establish transplantable lines; however, latency and variable penetrance limited their suitability for the timelines and therapeutic testing required in this revision.

2. The authors do an exceptional job of validating GPNMB expression in GBM cells and TAMs, both using RNA and protein data and many relevant controls. However, the cell surface expression and antibodies studies (Extended Data Fig. 3) are much less robust. It would be nice to see some additional models (mouse/human GBM) and relevant controls (astrocytes, NSCs, other immune lineages) to show cell surface expression via flow cytometry.

Response: We thank the reviewer for this comment. As suggested, we have assayed GPNMB expression by flow cytometry across 15 patient GSC samples, normal CNS cells (astrocytes and fetal neural stem cells), and benchmarked against melanoma and breast cancer cell lines (**Response Fig. 2**). These data confirm low-to-undetectable surface GPNMB on normal healthy cells, and substantially increased expression in GBM samples. Flow data have been included as the new Figure 1C in the revised manuscript.

3. The claims in GPNMB targeting impacting both GBM cells and TAMs could be further strengthened by doing some studies with the CAR T cells in the context of TAM depletion (humanized mouse model would be an obvious choice, but in an immune competent mouse model would be fine as well). This would help show the extent to which survival is impacted by TAM targeting via GPNMB.

Response: We thank the reviewer for this very insightful recommendation. To compare the effectiveness of GPNMB CAR-T targeting in the presence or absence of macrophages, we implemented an intracranial GBM cell + macrophage co-injection model in NSG mice, using GBM8-ffluc GSCs combined with immunosuppressive cytokine-conditioned U937 macrophages. Given the paucity of functional endogenous bone marrow-derived macrophages in NSG mice, this approach enables the introduction of a defined immunosuppressive macrophage compartment while maintaining a human GBM tumor model. Consistent with the established pro-tumorigenic role of macrophages in GBM,[4] co-injection significantly accelerated tumor growth and reduced survival relative to macrophage-deficient (GBM8-only) tumors (**Response Fig. 3A-B**). Strikingly, treatment with GPNMB CAR-Ts resulted in complete clearance of both GPNMB+ tumor cells and $GPNMB^{+}IBA1^{+}$ macrophages (**Response Fig. 3C**), despite the co-injected cohort beginning treatment with ~10-fold higher tumor burden than the GBM8-only cohort. In addition, in our new murinized CAR-T studies performed in a fully immunocompetent syngeneic setting (described below), we likewise observe robust elimination of both GPNMB+ tumor cells and intratumoral macrophages (**Response Fig. 8D**). Together, these models strengthen the conclusion that anti-GPNMB CAR-T cells mediate dual targeting of malignant cells and macrophages *in vivo*, and that this dual activity contributes to prolonged survival.

4. I am not sure the GSC terminology is needed earlier in the paper and the focus could just be on targets in CD133- populations. This could be framed in the context of previous work from the Singh lab marking CAR T cells against CD133+ cells and then supporting the need to find targets for the CD133- population. In line with this, I am a bit confused by the data in Extended Data Fig 5b as GPNMB was identified as the top hit in CD133- cells but

these data show it is present in a subset of CD133+ cells. This would be worth a brief explanation.

Response: We thank the reviewer for their thoughtful feedback, and have used it to center the clinical rationale around antigen escape from CD133-directed therapy. In line with the data in Figure 1A, we emphasize rebound tumors post-CD133 CAR-T treatment are $CD133^{-}$ and diffusely $GPNMB^{+}$ (**Response Fig. 4A-B**), supporting GPNMB as a rational candidate to address the post-CD133 CAR-T disease state, as postulated by the reviewer. Indeed, sequential administration of GPNMB CAR-Ts following an initial CD133 CAR-T regimen induced sustained remission, despite starting from high tumor burden (**Response Fig. 4C-D**). Regarding the questions raised by Extended Data Fig. 5b in the previous manuscript, we agree this requires clarification. Our identification of GPNMB as an enriched hit in $CD133^{-}$ cells reflects relative enrichment within the $CD133^{-}$ compartment, rather than exclusivity to $CD133^{-}$ cells. Accordingly, a subset of $CD133^{+}$ cells still express GPNMB. To avoid confusion, we have removed the data in Extended Data Fig. 5b from the revised manuscript.

Referee #2 (Remarks to the Author):

In the submitted manuscript, the authors report on the role on GPNMB in GBM, demonstrate that it is expressed in TAMs, develop GPNMB-CARTs to deplete TAMs, and perform in vitro as well as in vivo studies. While the studies are well conducted and interesting, there are significant concerns.

Major concerns:

1. Limited conceptual novelty: other investigators have published on the role of GPNMB+ macrophages in promoting tumor progression including for GBM (e.g., https://doi.org/10.1186/s40478-024-01754-7). Likewise, targeting TAMs with CARTs has been explored by others (e.g., https://doi.org/10.1158/2326-6066.CIR-21-1075).

Response: We thank the reviewer for raising this point. Indeed, there have been a number of recent studies supporting the functional role of $GPNMB^{+}$ macrophage in GBM progression; however, none have designed or tested GPNMB-directed therapies. The central advance of our manuscript is the nomination of GPNMB as an actionable cell-surface antigen shared by malignant cells and pro-tumorigenic TAMs, and the development of an anti-GPNMB therapy that functionally eliminates both compartments. Dual-compartment targeting is a key and novel differentiator from tumor-restricted or macrophage-restricted approaches, such as F4/80 CARs or TAM depletion, which did not show clinical trial success.[5] Given myeloid-dominant immunosuppressive TMEs are increasingly recognized as a major barrier to effective immunotherapy in solid tumors, a single therapeutic that simultaneously targets both malignant and suppressive TAMs is a significant conceptual and translational advance. Consistent with this rationale, GPNMB CAR-Ts have demonstrated robust anti-tumor activity across multiple preclinical and immunocompetent models, with high translational potential.

2. Limited novelty regarding T cell engineering: no novel CAR design or genetic engineering approach of CARTs is explored.

Response: We agree with the reviewer on this point, although CAR engineering is beyond the scope of this manuscript. The primary focus of this study is target identification and validation. However, we agree entirely with the reviewer, and have included discussion on the design of novel CAR engineering approaches towards clinical translation. This includes autologous “off-the-shelf” CAR-Ts, or the use of logic gated CARs to further ameliorate safety concerns.

3. Tumour models: the anti-tumour activity of GPNMB-CARTs as a single agent or in combination with CD133-CARTs is limited. This raises significant concerns. It appears that CARTs derived from only one donor were evaluated. Please provide data for at least two independent donors.

Response: We thank the reviewer for highlighting these issues regarding the strength of anti-tumor response of GPNMB CAR-Ts. Since these studies were initially conducted, we have substantially improved our CAR-T manufacturing workflow using new expansion reagents, as evidenced by our recent publication.[6] These improvements yielded a higher quality final GPNMB CAR-T product that we tested *in vivo* in the updated manuscript. With optimized manufacturing, GPNMB CAR-T treatment produced a striking anti-tumor response and is fully curative in multiple PDX GBM models, with tumor control extending beyond 100-150 days (**Response Fig. 5)**. Additionally, to rule out donor dependence, we generated GPNMB CAR-Ts from two additional healthy donors (Leuk16 and PC63), which show equivalent efficacy, activation, and cytokine release as Leuk17-derived CAR-Ts used in the initial experiments (**Response Fig. 6)**.

4. The authors use immunodeficient xenograft models to study the immunosuppressive tumor microenvironment and the effects of CARTs on tumour associated macrophages. The tumour microenvironment in immunodeficient xenograft models is not representative of human tumours and it is critical to conduct studies in immune competent animal models.

Response: We thank the reviewer for this important comment and agree on immunocompetent testing. In response, we have substantially expanded the revised study to include rigorous syngeneic testing.

Specifically, we generated and validated a murinized anti-mouse GPNMB CAR-T using the binder sequence derived from the commercial antibody clone CSTREVL (**Response Fig. 7A-B**). We confirmed specificity and on-target activity using $GPNMB^{+}$ or $GPNMB^{KO}$ B16F10 cells (**Response Fig. 7C**), and demonstrated potent cytotoxicity against the GL261 murine glioma model *in vitro* (**Response Fig. 7D**). In C57BL/6 mice bearing orthotopic GL261 tumors, murinized GPNMB CAR-T treatment elicited a strong therapeutic response and achieved durable, long-term tumor control exceeding 100 days without any toxicities (**Response Fig. 8A-C**).

To directly assess effects on the TME, we analyzed brains from control and treated animals at early post-treatment time points (6- and 10-days post tumor inoculation), as well as at endpoint in control mice. Consistent with our observations in the xenograft co-injection setting, we find robust depletion of both $GPNMB^{+}IBA1^{-}$ tumor cells and $GPNMB^{+}IBA1^{+}$ macrophages following CAR-T treatment (**Response Fig. 8D**). Together, these data demonstrate GPNMB CAR-T therapy retains potent efficacy in an intact immune system and mediates dual targeting of tumor and myeloid compartments within a representative syngeneic GBM microenvironment.

Minor concerns:

1. The construction of the CAR is not described in the material and methods section.

2. Limited information is provided on the generated CART products (e.g., efficiency, T cell phenotype, etc).

3. Fig.. 3e,f: it is stated that experimental replicates are shown. Please show data for at least three different donors.

4. It appears that CAR expression is not directly demonstrated and only one example is shown (Extended Figure 3). Please show directly that the CAR is expressed and show data for multiple donors.

Response: We sincerely thank the reviewer for catching these oversights. We have addressed these in the revised manuscript. We have included more information on CAR construction in the methods section, and included CAR expression (**Response Fig. 6A**), efficacy (**Response Fig 6B-G**), and phenotypic profiling (**Response Fig. 12A**) data for a total of 3 CAR-T donors.

Referee #3 (Remarks to the Author):

The authors explore GPNMB as a potential target in glioblastoma because it is expressed in GBM and TAMs using various datasets and also a series of biobanked specimens. However, GPNMB has already been identified as a promising target in various cancers, including GBM, as per the referenced literature in the paper. There have also already been trials of antibodies targeting GPNMB (glembatumumab vedotin), which have been unsuccessful. The authors here generate CARs targeting GPNMB using the same binding regions as the previously tested antibody. They also hypothesize that because of existing literature indicating that tumor-associated macrophages express GPNMB, that the CAR-T cells would target both the GBM and its associated TAMs, and therefore a single CAR would function as a combination strategy. However, the manuscript has modest novelty since nowadays building a CAR to an established antigen, using binding domains from an established antibody, and testing it in vitro and in vivo is fairly routine. Furthermore, the anti-tumor effects in the in vivo experiments was quite modest as well.

Response: We thank the reviewer for their comments and feedback. We agree GPNMB has been studied in multiple solid tumors, and antibody-based targeting strategies have seen limited clinical success. We view this prior work as important orthogonal validation that GPNMB is a clinically relevant antigen; however, these efforts do not establish strong GPNMB-directed therapies. In this context, the principal contribution of our study is testing the first GPNMB CAR-T in GBM for single-product, dual-compartment targeting of malignant GBM cells and pro-tumorigenic TAMs. Given myeloid-dominant, suppressive microenvironments are increasingly recognized as a key barrier to effective immunotherapy in solid tumors, we believe a strategy that concomitantly debulks tumor cells while eliminating suppressive TAMs represents a meaningful conceptual and translational advance in GBM beyond prior GPNMB biology or antibody-based approaches.

1. GPNMB is thought to play a critical role in the survival of motor neurons, and is thought to potentially be associated with ALS. The authors should consider whether targeting this molecule could result in death of motor neurons, which could be a severe toxicity. Furthermore, on-target toxicities toward bone and skin could be serious (and the skin toxicity was a dose-limiting toxicity of previous antibodies). Its confirmation here is not reassuring. The tissue microarray should also include motor neurons.

Response: We thank the reviewer for raising important safety considerations. We previously stained several brain regions by immunohistochemistry and did not detect GPNMB expression in normal brain parenchyma (**Response Fig. 9**). In addition, in our expanded immunocompetent syngeneic CAR-T studies, we stained whole-mouse brain sections for GPNMB and again observed no detectable expression throughout cortical tissue, with signal restricted to the tumor graft (**Response Fig. 8D**). Consistent with these findings, animals treated with murinized GPNMB CAR-Ts were tumor-free without toxicity (**Response Fig. 8C**). We also expanded our flow cytometry panel to include non-malignant human CNS cell controls (fetal neural stem cells, differentiated neural stem cells, and primary human astrocytes), all of which showed virtually

undetectable GPNMB surface expression (**Response Fig 2**). With respect to skin toxicity, our co-submission, Zemp *et al.* performed the first-in-human trial of IV-infused GPNMB CAR-Ts with no significant skin or neurological toxicity, and observed no toxicity in a skin graft mouse model. In all, these data support the safety of GPNMB CAR-T therapy.

2. The biologic significance of GPNMB being upregulated in recurrent as opposed to primary GBM is not clear. Both kinds of GBM are highly proliferative, so the role of GPNMB as being important for proliferation of GBM is important but not necessarily connected?

Response: We thank the reviewer for this insightful comment. We agree increased GPNMB expression at recurrence is likely independent of its role in proliferation. Rather, our interpretation is that GPNMB is upregulated post-treatment owing to well-described shifts in tumor cell state and microenvironment upon recurrence: Neftel et al.[7] described a recurring set of GBM transcriptional programs comprising neural progenitor cell–like (NPC-like), oligodendrocyte progenitor cell–like (OPC-like), astrocyte-like (AC-like), and mesenchymal-like (MES-like) states that can co-exist within individual tumors. Of these states, MES-like are often seen as the most aggressive and treatment refractory.[8,9] Several groups have reported enrichment of MES-like cells post-treatment[10-12]. In parallel, MES-like programs can be promoted by immune and myeloid-derived cues[10,13,14], and recurrent GBM is characterized by increased myeloid infiltration[10].

Projecting these transcriptional states onto GBM scRNA-seq data[1,3,7], we found GPNMB expression is highest in MES-like cancer cells (**Response Fig. 10**). Additionally, in the scRNA-seq analysis of our *Gpnmb*^{KO} and WT murine glioma tumors, we observed *Gpnmb*^{KO} show an enrichment in development (NPC- and OPC-like) and astrocyte-like states, but crucially, not MES-like states (**Response Fig. 11**), suggesting *Gpnmb* helps maintain MES-like states, which dominate at recurrence.

3. Is there a knockout mouse of GPNMB?

Response: Yes, GPNMB^{KO} mice have been described in the literature, though are more often used in the context of obesity research. These reports largely agree GPNMB loss-of-function leads to heightened inflammatory phenotypes in obese mice,[15,16] further supporting the role of GPNMB^{+} macrophages as an immunosuppressive population. A recent study employed GPNMB^{KO} mice in epilepsy models, finding GPNMB^{KO} microglia display attenuated phagocytosis of degenerated neuronal debris, and GPNMB knockdown in the microglia-like cell line BV2 resulted in increased IL6 and TNFα expression.[17]

4. In Figure 1, what is the level of expression GPNMB in GBM relative to melanoma and breast cancer, where GV has already been tested?

Response: We have profiled surface GPNMB expression in 15 patient-derived GSC samples, fetal neural stem cells, normal human astrocytes, and benchmarked expression against melanoma and breast cancer lines as suggested by the reviewer (**Response Fig. 2**). We observed high GPNMB positivity in most GSCs, and several were comparable to SK-MEL-2 melanoma cells. In contrast, the MDA-MB-231 line displayed only modest GPNMB surface positivity. These data have been included as Fig. 1C in the revised manuscript.

5. In Figure 3, the GPNMB-CAR-T should also be tested against the GPNMB-KO glioma as target cells, since the authors already made it and would confirm the CAR's specificity.

Response: We generated GPNMB^{KO} GBM8-ffluc cells, and confirmed cytotoxicity of CAR-Ts is substantially lower across all E:T ratios (**Response Fig. 12B-C**). These data have been included Extended Data Fig. 3g-h the revised manuscript.

6. Figure 4—although there is improved survival and some delay in tumor progression, it doesn't seem like either single or dual targeting of GPNMB +/- CD133 induces sustained responses.

Response: The point of modest CAR-T activity *in vivo* is well taken. In the revised work, we addressed this by repeating and expanding *in vivo* studies using an improved CAR-T manufacturing protocol we have implemented since the original studies were conducted (as described earlier). Using these optimized GPNMB CAR-Ts, we now observe considerably enhanced anti-tumor activity that is reproducible across multiple independent CAR-T donors. Importantly, *in vivo* testing in multiple GBM PDXs demonstrates durable tumor control, including sustained responses extending beyond 100 days in several models.

7. The abbreviation of the "huNOG-EXL mouse model" is not a strain that is in frequent use in the US. Can you please use the more common strain name or describe what it is transgenic for and what it is deficient in in the main text?

Response: We thank the reviewer for this clarification. HuNOG-EXL mice (Taconic Biosciences) are transgenic NOG mice expressing human *IL3* and *CSF1*, transplanted with CD34^{+} HSCs[18]. We have clarified strain details in the main text.

8. In Figure 5, are the T cells used autologous to the humanized mouse? Allogeneic to the tumor? Again, the degree of tumor control as shown in Fig. 5a and 5b is quite modest.

Response: The T cells used in Figure 5 are allogeneic to the humanized mice and tumor. We agree the degree of tumor control in this experiment is quite modest, reflecting the variability and technical challenges of humanized mouse models for intracranial GBM. To further validate the dual targeting capacity of GPNMB CAR-Ts, we have introduced several new *in vivo* models. First, we used an intracranial co-inoculation model in NSG mice using ffluc-expressing GSCs with immunosuppressive cytokine–conditioned macrophages to impart a defined myeloid compartment. In this setting, macrophage co-injection markedly accelerated tumor growth (some mice reached 10^9 flux just 3 days post-inoculation, for reference, mice typically reach symptomatic endpoint at 10^{10}-10^{11} flux), yet GPNMB CAR-T treatment rapidly eliminated tumor signal up to 100,000-fold within 1 week (**Response Fig. 3**). Second, we generated a murinized anti-mouse GPNMB CAR-T (**Response Fig. 7**) and tested it in an immunocompetent, syngeneic GL261 model, as described earlier. In C57BL/6 mice, autologous murinized GPNMB CAR-Ts achieved long-term tumor control and demonstrated robust elimination of both GPNMB^{+}/IBA1^{-} tumor cells and GPNMB^{+}/IBA1^{+} TAMs (**Response Fig. 8**). Collectively, these complementary *in vivo* studies strengthen the conclusion that GPNMB CAR-T cells can mediate anti-tumor activity in immunocompetent settings.

Minor points:

1. Please don't invent a new acronym. "Tumor micronvironment" is often already abbreviated as "TME", it is confusing to call it "tumor immune microenvironment" and abbreviate it as "TIME."

Response: We thank the reviewer for this feedback, and have reverted to the TME abbreviation throughout the revised manuscript.

2. Page 17 – “the feasibility of dual-targeting CAR-T cells with two single-domain antibodies has already been shown by the dramatic clinical success and commercial approval of cilta-cel.” This is a mistake. Cilta-cel is not dual-targeting of two different antigens, it is dual targeting of two epitopes of the same antigen (biparatopic).

Response: We thank the reviewer for catching this oversight, and have removed this in the revised manuscript.

References

1 Mikolajewicz, N. *et al.* Functional profiling of murine glioma models highlights targetable immune evasion phenotypes. *Acta Neuropathol* **148**, 74 (2024). https://doi.org/10.1007/s00401-024-02831-w
2 Yu, K. *et al.* Surveying brain tumor heterogeneity by single-cell RNA-sequencing of multi-sector biopsies. *Natl Sci Rev* **7**, 1306–1318 (2020). https://doi.org/10.1093/nsr/nwaa099
3 Abdelfattah, N. *et al.* Single-cell analysis of human glioma and immune cells identifies S100A4 as an immunotherapy target. *Nature Communications* **13** (2022). https://doi.org/10.1038/s41467-022-28372-y
4 Gu, D. *et al.* Stress-induced pro-inflammatory glioblastoma stem cells secrete TNFAIP6 to enhance tumor growth and induce suppressive macrophages. *Dev Cell* (2025). https://doi.org/10.1016/j.devcel.2025.04.027
5 Butowski, N. *et al.* Orally administered colony stimulating factor 1 receptor inhibitor PLX3397 in recurrent glioblastoma: an Ivy Foundation Early Phase Clinical Trials Consortium phase II study. *Neuro Oncol* **18**, 557–564 (2016). https://doi.org/10.1093/neuonc/nov245
6 Chokshi, C. R. *et al.*Targeting axonal guidance dependencies in glioblastoma with ROBO1 CAR T cells. *Nature Medicine* (2024). https://doi.org/10.1038/s41591-024-03138-9
7 Neftel, C. *et al.* An Integrative Model of Cellular States, Plasticity, and Genetics for Glioblastoma. *Cell* **178**, 835–849.e821 (2019). https://doi.org/10.1016/j.cell.2019.06.024
8 Bhat, K. P. L. *et al.* Mesenchymal differentiation mediated by NF-kappaB promotes radiation resistance in glioblastoma. *Cancer Cell* **24**, 331–346 (2013). https://doi.org/10.1016/j.ccr.2013.08.001
9 Carro, M. S. *et al.* The transcriptional network for mesenchymal transformation of brain tumours. *Nature* **463**, 318–325 (2010). https://doi.org/10.1038/nature08712
10 Wang, Q. *et al.* Tumor Evolution of Glioma-Intrinsic Gene Expression Subtypes Associates with Immunological Changes in the Microenvironment. *Cancer Cell* **32**, 42–56 e46 (2017). https://doi.org/10.1016/j.ccell.2017.06.003
11 Wang, L. *et al.* A single-cell atlas of glioblastoma evolution under therapy reveals cell-intrinsic and cell-extrinsic therapeutic targets. *Nat Cancer* **3**, 1534–1552 (2022). https://doi.org/10.1038/s43018-022-00475-x
12 Hendriksen, J. D. *et al.* Immunotherapy drives mesenchymal tumor cell state shift and TME immune response in glioblastoma patients. *Neuro Oncol* **26**, 1453–1466 (2024). https://doi.org/10.1093/neuonc/noae085
13 Hara, T. *et al.* Interactions between cancer cells and immune cells drive transitions to mesenchymal-like states in glioblastoma. *Cancer Cell* **39**, 779–792 e711 (2021). https://doi.org/10.1016/j.ccell.2021.05.002
14 Varn, F. S. *et al.* Glioma progression is shaped by genetic evolution and microenvironment interactions. *Cell* **185**, 2184–2199 e2116 (2022). https://doi.org/10.1016/j.cell.2022.04.038
15 Prabata, A., Ikeda, K., Rahardini, E. P., Hirata, K. I. & Emoto, N. GPNMB plays a protective role against obesity-related metabolic disorders by reducing macrophage inflammatory capacity. *J Biol Chem* **297**, 101232 (2021). https://doi.org/10.1016/j.jbc.2021.101232
16 Nickl, B., Qadri, F. & Bader, M. Anti-inflammatory role of Gpnmb in adipose tissue of mice. *Sci Rep* **11**, 19614 (2021). https://doi.org/10.1038/s41598-021-99090-6
17 Liu, M. *et al.* GPNMB and ATP6V1A interact to mediate microglia phagocytosis of multiple types of pathological particles. *Cell Rep* **44**, 115343 (2025). https://doi.org/10.1016/j.celrep.2025.115343
18 Willis, E. *et al.* Humanization with CD34-positive hematopoietic stem cells in NOG-EXL mice results in improved long-term survival and less severe myeloid cell hyperactivation phenotype relative to NSG-SGM3 mice. *Vet Pathol* **61**, 664–674 (2024). https://doi.org/10.1177/03009858231222216

FIGURE REDACTED

TEXT REDACTED

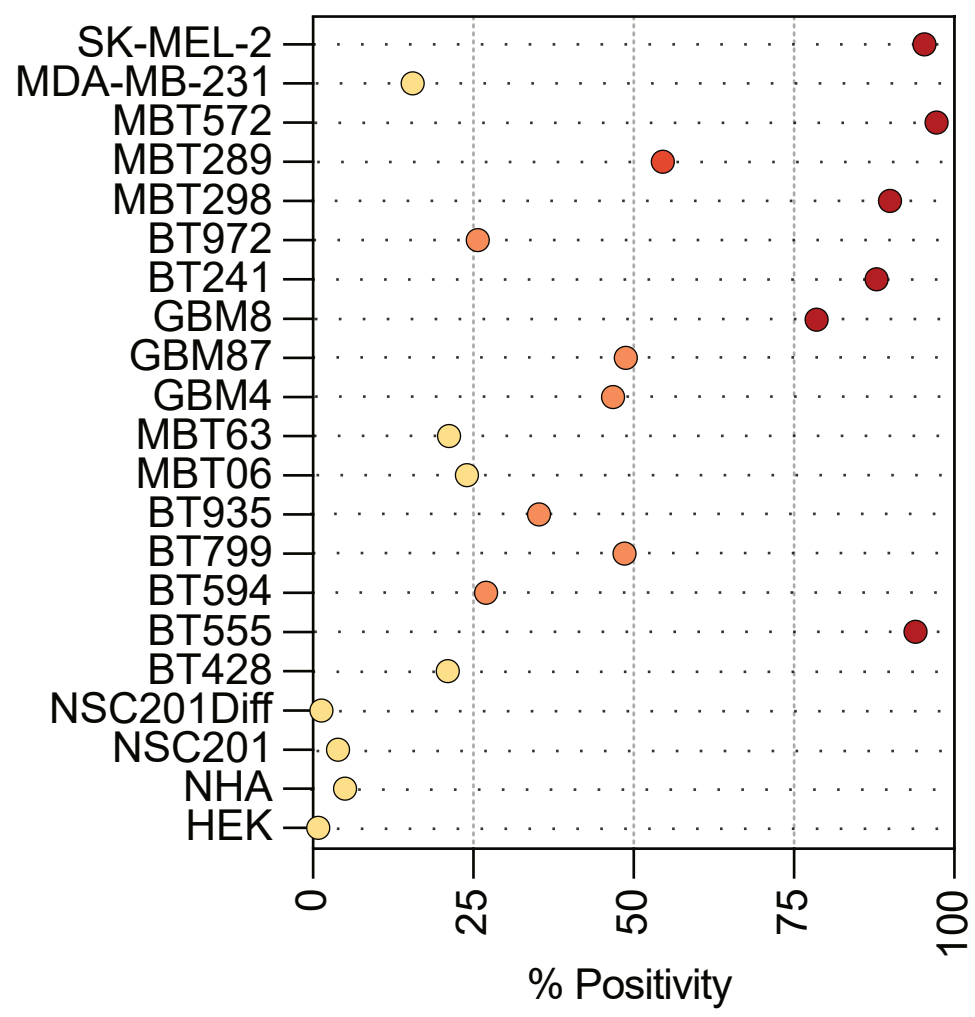

Response Figure 2. GPNMB flow cytometry characterization. Non-cancerous cells (normal human astrocytes (NHA), fetal neural stem cells (NSC), or differentiated NSCs), patient GSC samples, and a melanoma (SK-MEL-2) and breast cancer (MDA-MB-231) cell line where characterized for surface GPNMB by flow cytometry using the HOST5DS commercial antibody clone. Response Figure 2 has been included as Figure 1C in the revised manuscript.

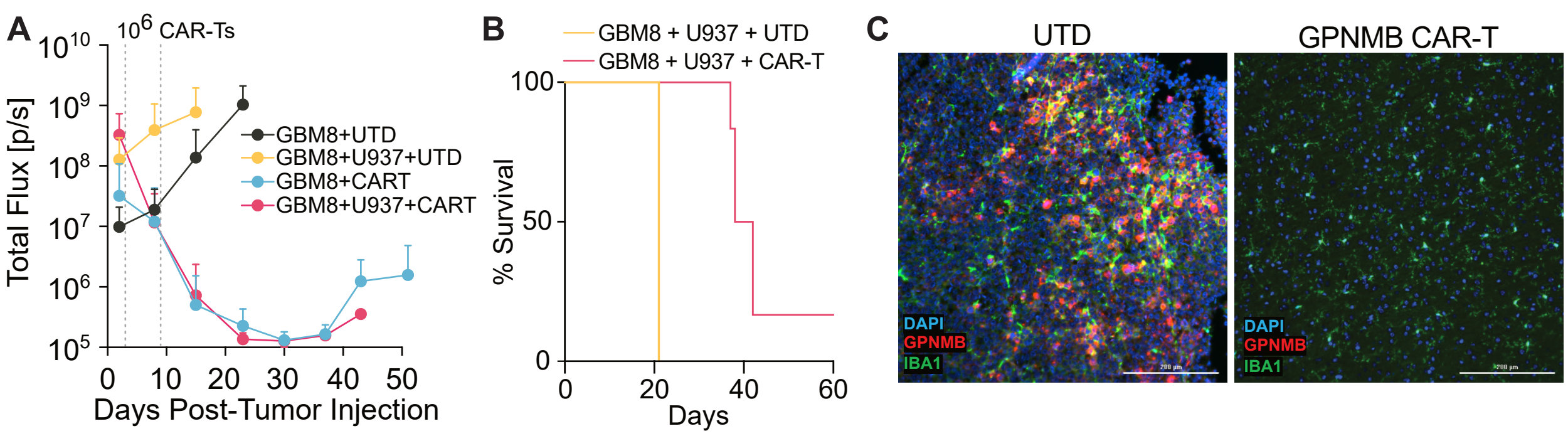

Response Figure 3. GBM8-U937 co-injection model. (A-B) 1x10[5] GBM8-ffluc cells were intracranially injected either alone, or with equal number of IL4/IL10/TGFb-conditioned U937 macrophages, then treated with two doses of UTD or GPNMB CAR-T cells. (C) Immunofluorescence analysis of GPNMB and IBA1 in co-injected untransduced (*top*) or GPNMB CAR-T (*bottom*) treated mice. These data have been included as Figures 4h-j in the revised manuscript.

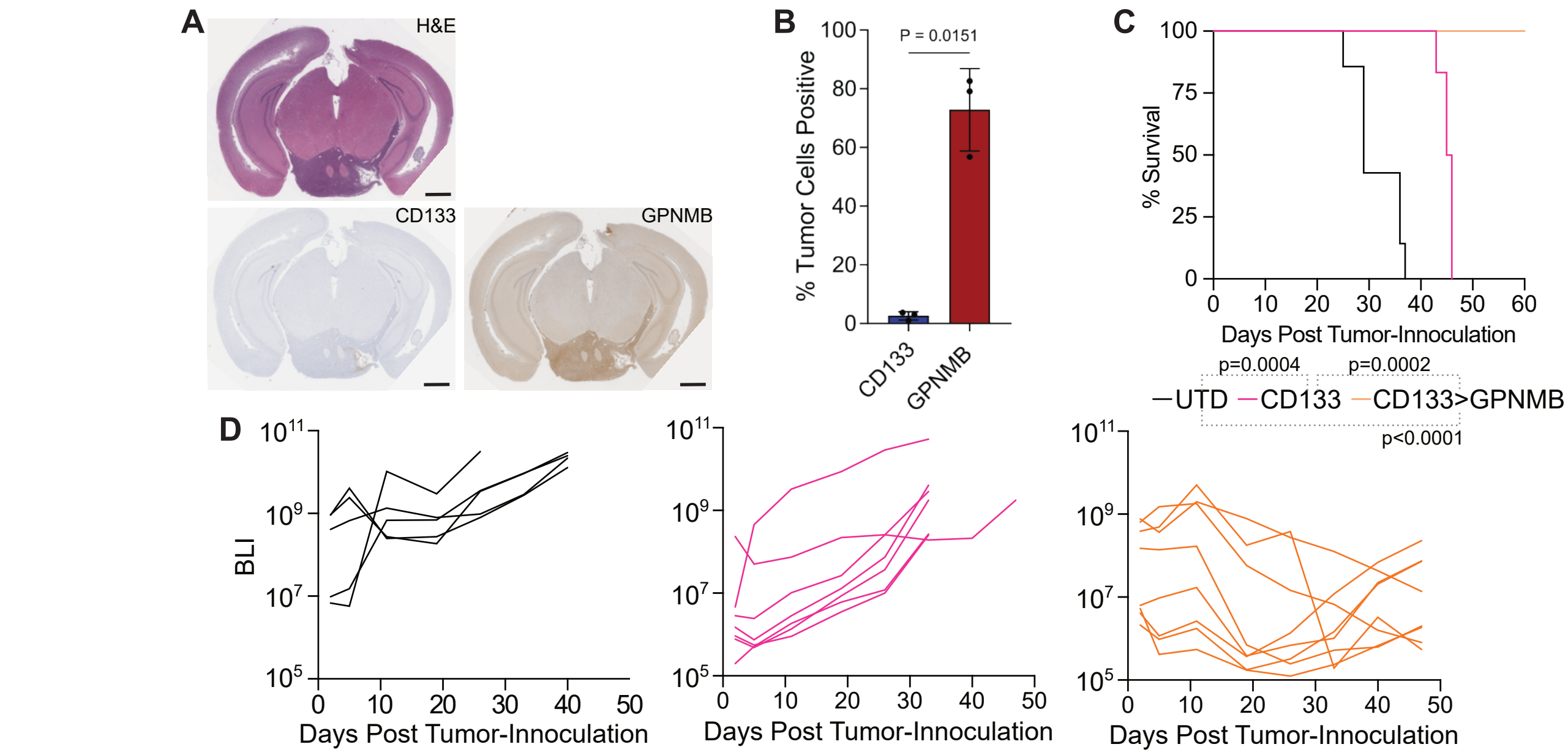

Response Figure 4. GPNMB CAR-Ts clear residual tumors following CD133 CAR-T treatment. (A) Representative IHC analysis of post-CD133 CAR-T rebound tumors. **(B)** Quantification of CD133 or GPNMB positive tumor cells in rebound tumors. Each dot is the average positivity from all tumor-burdened regions per mouse (n = 3). P value from paired T test of matched brains. Full data presented in Extended Data Fig 6 in the revised manuscript. **(C-D)** Evaluation of sequential CD133-to-GPNMB CAR-T therapy in orthotopic GBM PDX models. Mice engrafted with $1x10^5$ GBM8-ffluc were treated intracranially with 2 doses of $1x10^6$ CD133 CAR-Ts alone (pink), or followed up with 2 doses of $1x10^6$ GPNMB CAR-Ts (orange). These data are presented in Figure 3l-m in the revised manuscript.

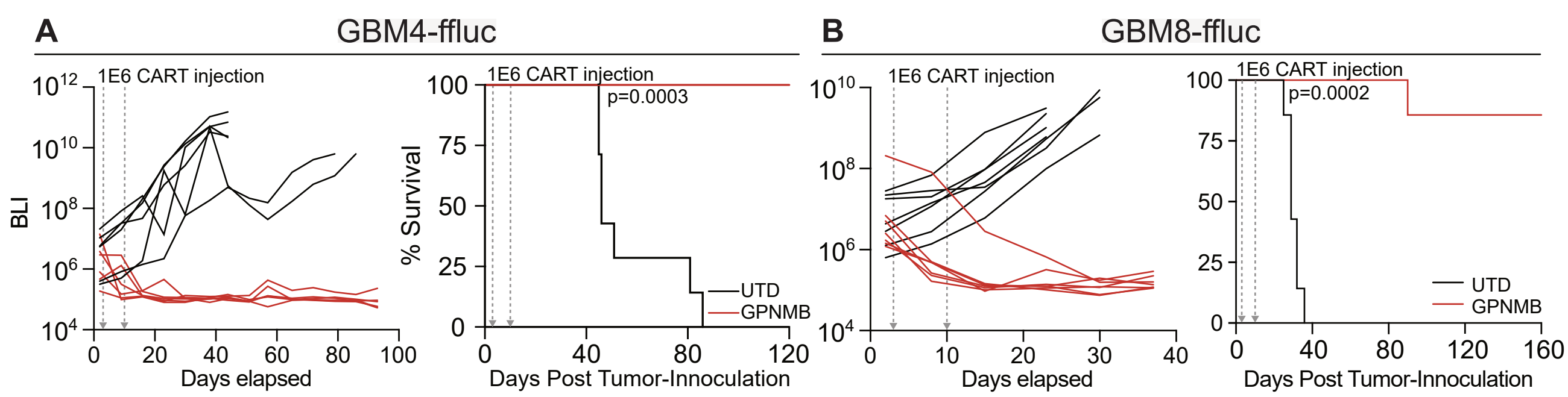

Response Figure 5. Optimized GPNMB CAR-T manufacturing yields curative response in GBM PDX models. (A) GBM4-ffluc bearing mice were treated with 2 doses of 1x106 UTD or GPNMB CAR-T cells. Tumor bioluminescence (BLI) (A) and Kaplan-Meier survival analysis (B) are shown. **(B)** GBM8-ffluc bearing mice were treated as A-B. These data are presented in Figure 3g-j in the revised manuscript.

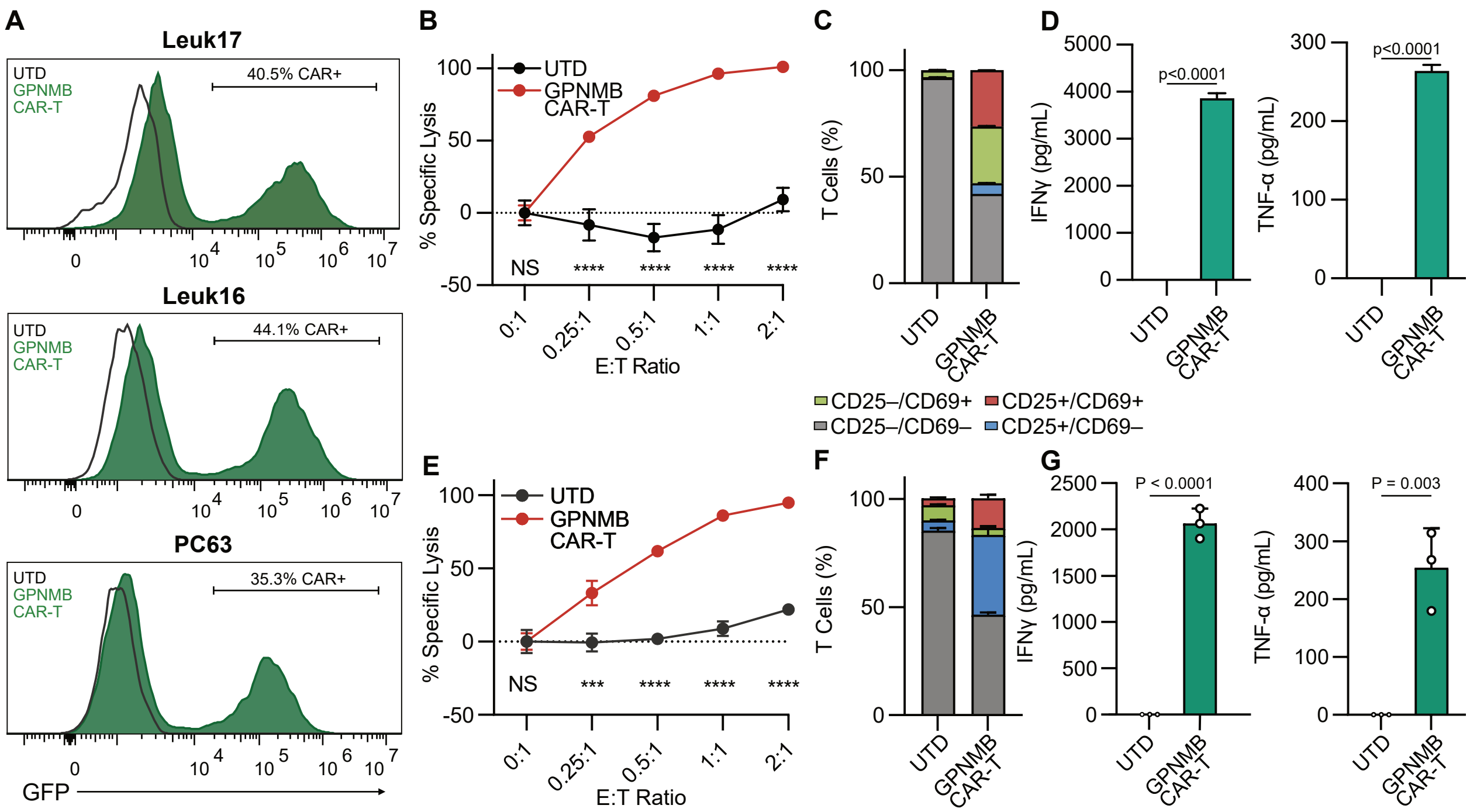

Response Figure 6. *In vitro* activity of additional CAR-T donors. (**A**) Flow cytometry of CAR transduction efficiency (GFP⁺) across 3 PBMC donors. (B-D) Cytotoxicity against GBM8 cells after 48hr coculture (B), and CD25/CD69 expression (C) and IFNy/TNFa secretion after 24hr coculture by Leuk16-derived CAR-Ts. (**E-H**) GPNMB CAR transduction efficiency (E), cytotoxicity against GBM8 cells after 48 hours of coculture (F), and CD25/CD69 expression (G) and IFNy/TNFa release (H) after 24 hours of coculture by PC63-derived CAR-Ts. These data, plus 2 additional GSC targets, are presented in Extended Data Figures 4-5 in the revised manuscript.

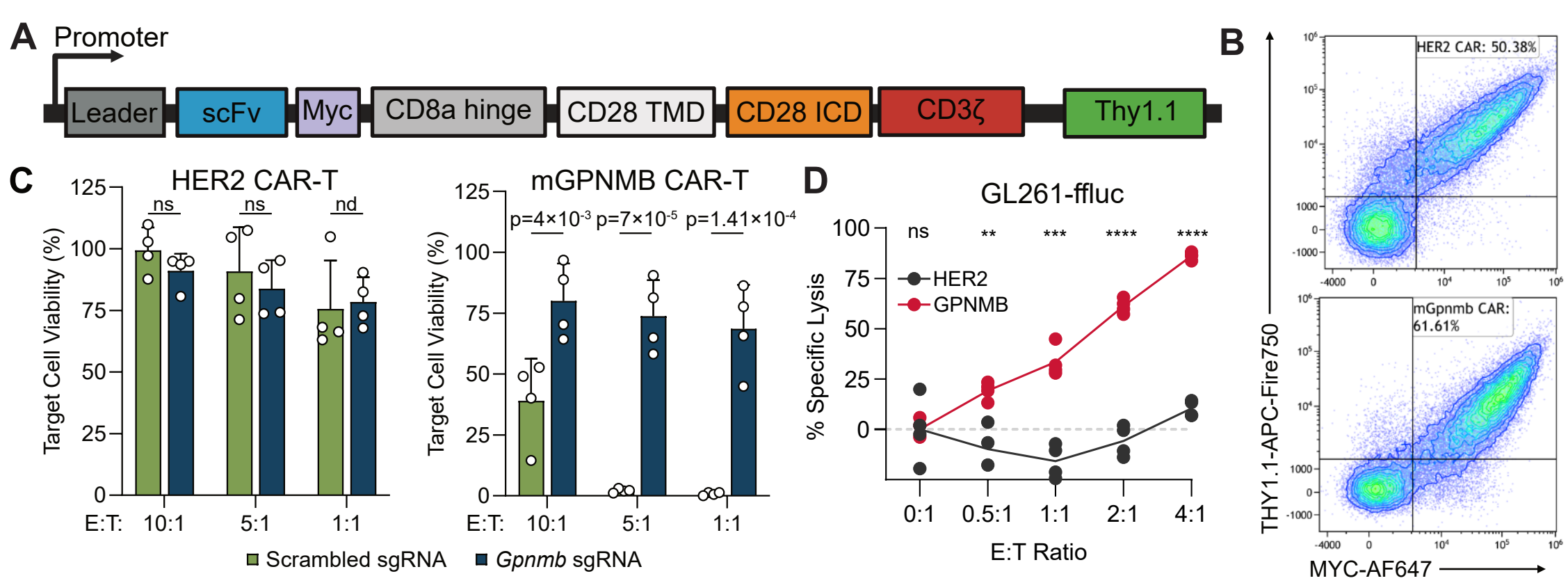

Response Figure 7. Development of a murinized anti-mouse GPNMB CAR-T cells. **(A)** Anti-mouse GPNMB (mGPNMB) CAR schematic. **(B)** Retroviral transduction of splenic Thy1.1⁺ T cells with Myc-tagged mGPNMB CAR vector. **(C)** Cytoxicity of mGPNMB CAR-T cells against B16F10 cells transduced with GPNMB-targeting or scrambled (scram) sgRNA. **(D)** *In vitro* cytotoxicity of anti-mGPNMB or non-targeting anti-HER2 CAR-T cells against GL261-ffluc murine glioma cells after 48 hours of coculture.

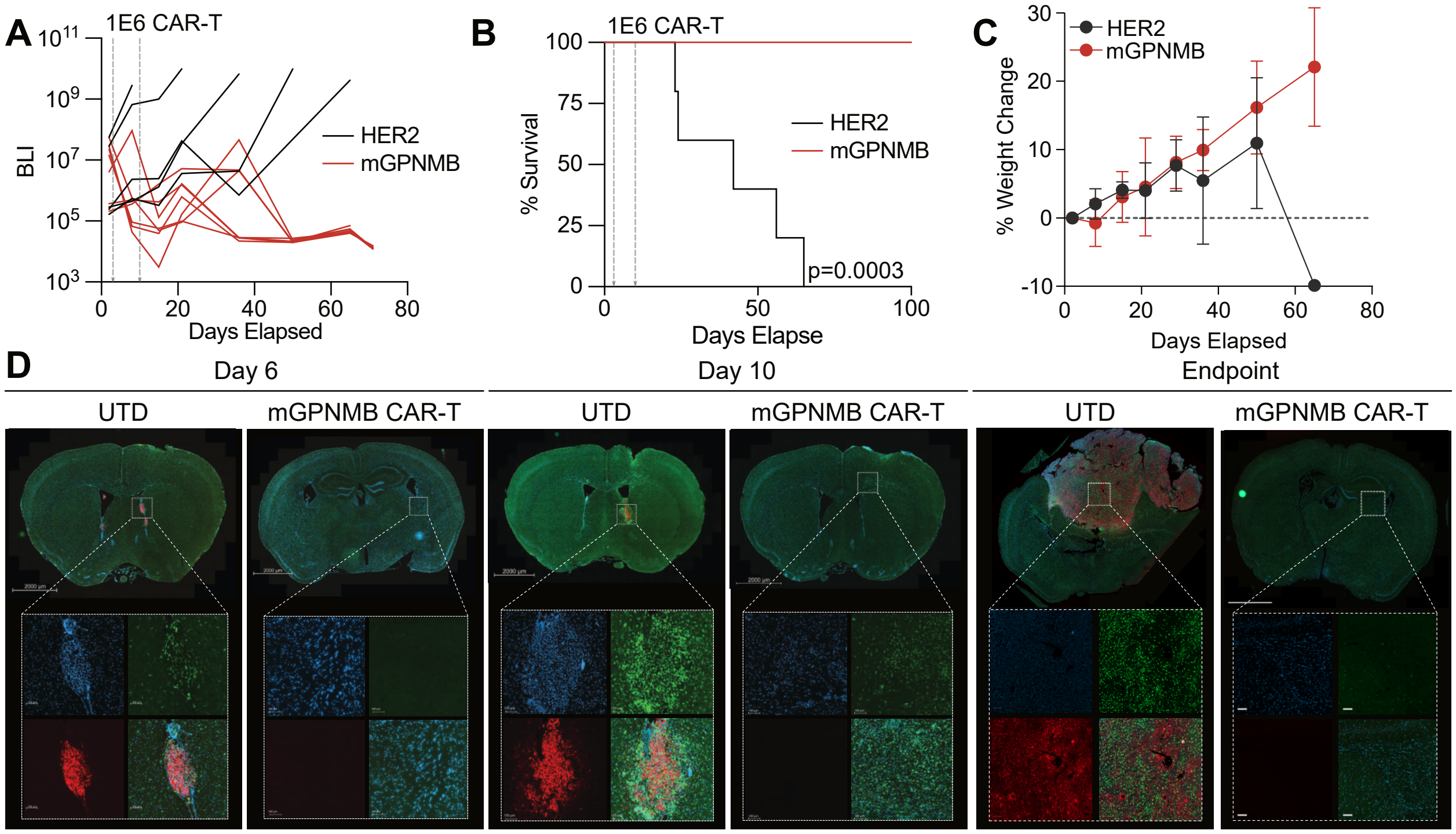

Response Figure 8. *In vivo* trial of anti-mGPNMB CAR-Ts against syngeneic GBM models. (A-B) Tumor bioluminescence (A) and Kaplan-Meier survival (B) of GL261^{ffluc}-bearing C57BL/6 mice treated with syngeneic non-targeting anti-HER2 (n=5) or anti-mGPNMB CAR-T (n=7) cells. **(C)** Weight change in mice over course of study. **(D)** Whole brain sections of GL261-bearing mice treated with control or anti-mGPNMB CAR-Ts 6-, 10-days post-tumor inoculation, or Day 28 (endpoint for UTD). DAPI = blue; GPNMB = red; IBA1 = green. These data are presented in Figure 5c-e and Extended Data Fig 11d-e in the revised manuscript.

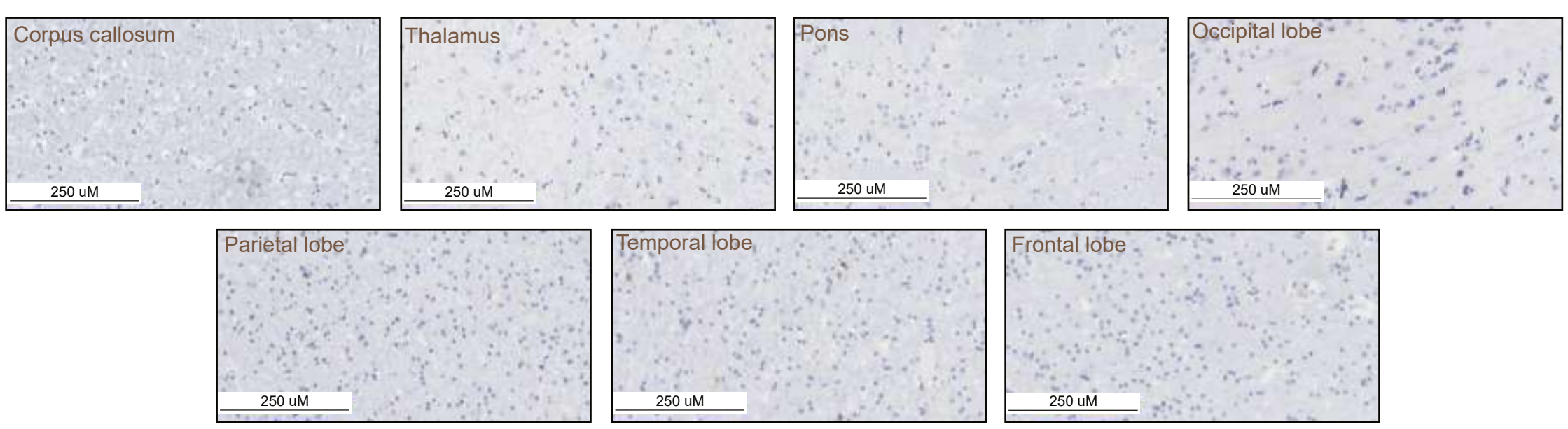

Response Figure 9. GPNMB IHC staining across healthy adult brain tissue. These data are presented in Extended Data Fig 1d.

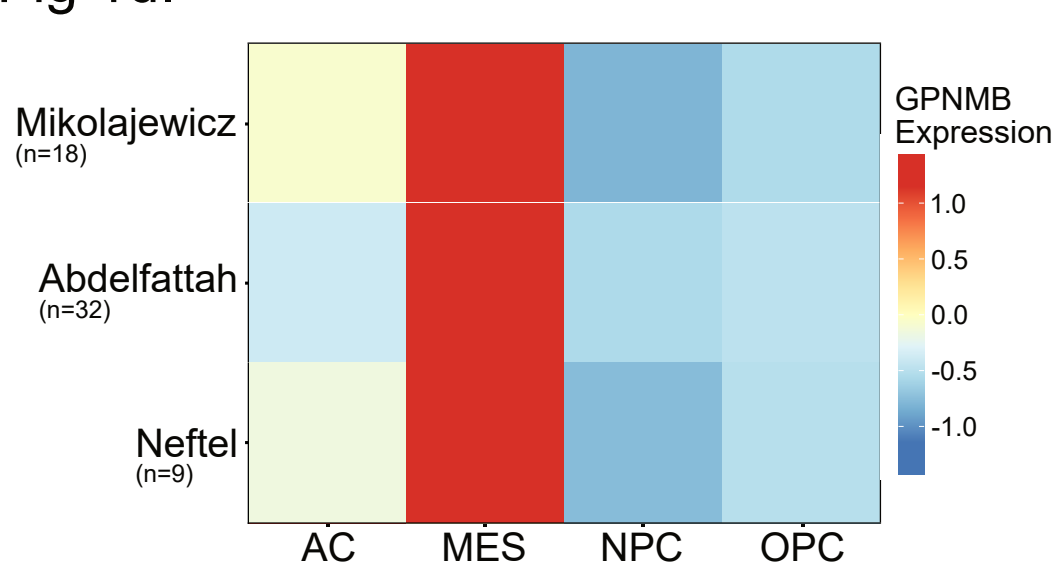

Response Figure 10. *GPNMB* expression between malignant cell states in scRNA-seq data. Scaled GPNMB (within each cohort) expression across astrocyte (AC)-like, mesenchymal (MES), neural progenitor (NPC), or oligodendrocyte progenitor (OPC)-like tumor cells.

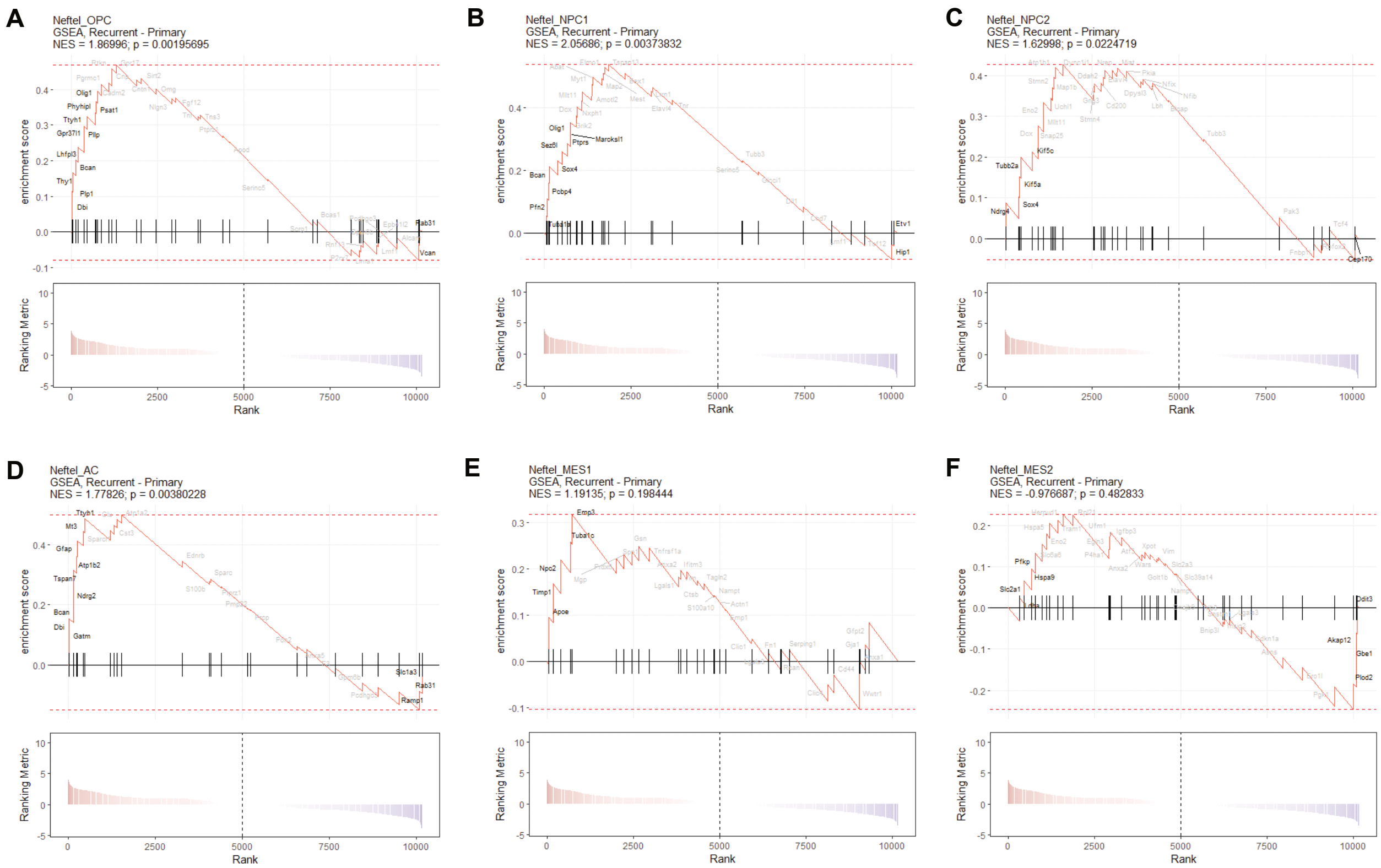

Response Figure 11. Cell state comparison between *Gpnmb*^{KO} and WT GL261 tumors. Neftel *et al.*[11] cell state gene sets compared between *Gpnmb*^{KO} and WT tumors. *Gpnmb*^{KO} tumors show enrichment of developmental (OPC, NPC1, NPC2) and astrocyte cell states (A-D), but not mesenchymal states (E-F). These data are presented in Extended Data Fig 2i-n in the revised manuscript.

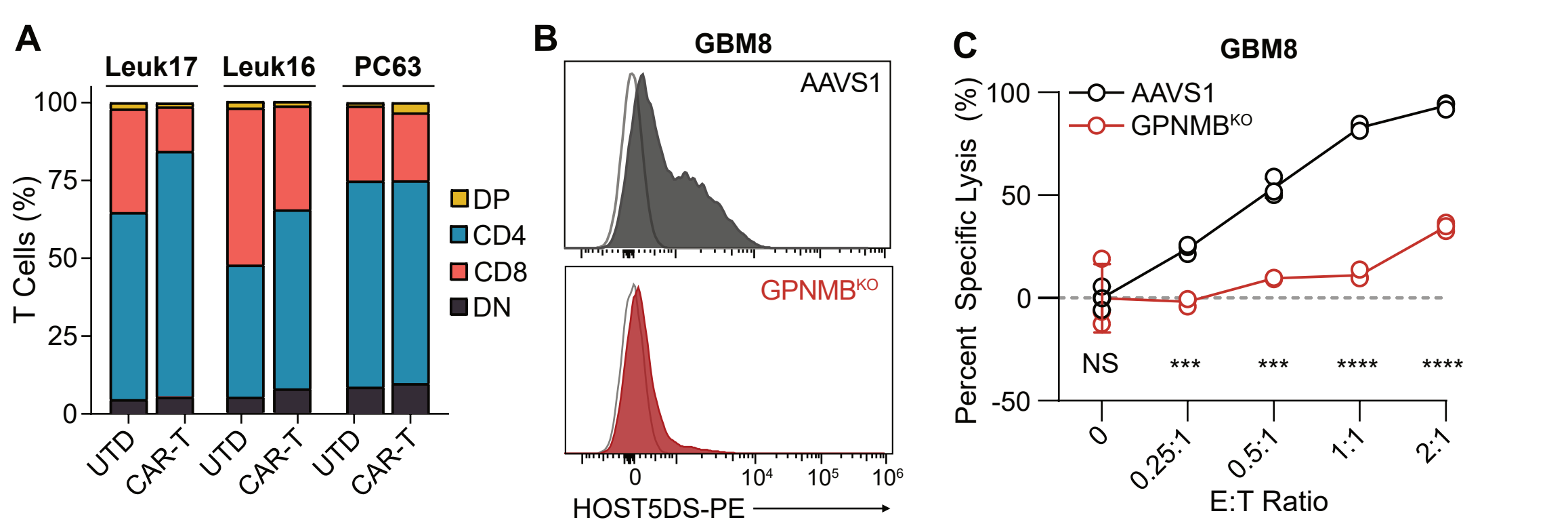

Response Figure 12. Specificity of GPNMB CAR-Ts to GPNMB^{+} GSCs. (A) CD4/CD8 phenotype of untransduced (UTD) and CAR-transduced products from each donor (double-negative [DN], CD4^{+}, CD8^{+}, double-positive [DP] fractions). **(B)** Surface GPNMB expression of GBM8-ffluc cells transduced with AAVS1 non-targeting or *GPNMB*-targeting sgRNA. **(C)** Cytotoxicity of GPNMB CAR-Ts cocultured with GBM8-ffluc cells transduced with non-targeting (AAVS1) or GPNMB-targeting sgRNA. These data are presented in Extended Data Fig 3g-h in the revised manuscript.

Response to Reviewers
Nature Manuscript # 2024-12-26538D

We thank the Editor and Reviewers for their evaluation of our revised manuscript. In this cycle, Referee #1 indicated all previously raised issues have been addressed, and Referee #3 notes that the manuscript has been substantially improved, with one additional request to ensure certain points raised in the rebuttal are also reflected in the manuscript. The remaining concerns are centered on Referee #2's assessment of conceptual novelty. As detailed below, we respectfully maintain that the novelty of our study is not the initial characterization of GPNMB biology, but the demonstration that a single CAR-T can exploit GPNMB as a shared, therapeutically actionable antigen to simultaneously target glioma stem cells (GSCs) and pro-tumorigenic macrophages *in vivo*, producing durable, curative outcomes across preclinical models. We further clarify how this differs from prior work on $GPNMB^+$ macrophages and from prior TAM-targeting CAR-T approaches.

Referee #1

"In general, all previously raised issues have been addressed."

Response: We thank the Reviewer for their reassessment of the revised manuscript. We appreciate the Reviewer's feedback throughout the process.

Referee #2

"I appreciate the authors' efforts in submitting a revised manuscript that addresses several of the concerns raised previously. Nevertheless, substantial issues remain. Foremost among these is the question of conceptual novelty. As noted in my initial review, prior studies have already characterized the role of $GPNMB^+$ macrophages in promoting tumor progression, including in GBM (e.g., PMID: 38566120) as well as in other malignancies. Additionally, the biology of GPNMB within the tumor microenvironment has been extensively investigated, with several of these findings summarized in PMID: 36050467. Moreover, as previously mentioned, the strategy of targeting tumor-associated macrophages with CAR T cells has been explored by multiple groups of investigators (e.g., PMID: 36095236; PMID: 33563975). Taken together, these observations continue to raise significant concerns regarding the level of novelty and, consequently, the suitability of this manuscript for a journal with the broad and general readership of Nature."

Response: We thank Referee #2 for their continued engagement and for acknowledging that the revision addressed several previously raised points. We have acknowledged the Reviewer's points in the Discussion of our revised manuscript (Lines 291-306; 312-316; 331-334). We respectfully disagree that the existence of prior literature on GPNMB biology or TAM-targeting CAR-T approaches negates the conceptual novelty and significance of our study.

We agree that GPNMB has been studied in other contexts and that $GPNMB^+$ macrophages have been implicated in tumor progression. Our work does not seek to re-establish these associations. Rather, the principal advance is demonstrating GPNMB is a therapeutically actionable, shared cell-surface antigen in GBM that can be exploited to achieve single-product, dual-compartment targeting. This strategy has not been demonstrated *in vivo* in solid tumors in the manner we report, and it directly addresses myeloid-mediated resistance, a widely recognized barrier to effective immunotherapy[1]. The cited studies on $GPNMB^+$ macrophages provide important context and are

appropriately cited in our manuscript. However, these studies do not evaluate a therapeutic approach that targets GPNMB to deliver concurrent cytotoxic activity against both malignant cells and suppressive myeloid populations.

Prior TAM-targeting CAR-T approaches are not equivalent to dual tumor-myeloid targeting with a single therapeutic. We agree that macrophage-directed CAR-T strategies have been explored previously, but are generally myeloid-restricted, and do not establish dual-compartment targeting of tumor cells and TAMs via a shared antigen. The recent publication of two independent TAM-targeting CAR-T studies in Cancer Cell[2,3] highlights broad interest in macrophage-directed cellular immunotherapy, even though both targets in these studies, FOLR2[4] and TREM2[5,6], have been extensively characterized already. Our work conceptually advances from TAM-only targeting to a dual-functional CAR-T strategy that eliminates GSCs and pro-tumorigenic TAMs. Moreover, the cited examples are not presented in the context of GBM—one of the most myeloid-rich, immunosuppressed tumors[7]—and do not demonstrate curative outcomes comparable to those achieved in our *in vivo* studies. On this note, while we recognize the value of CAR engineering, it would be challenging to test experimentally here as improvements may not be detectable in our *in vivo* systems given the potent efficacy achieved with the present CAR. We have, however, acknowledged CAR engineering as an important future direction towards clinical translation of anti-GPNMB CAR-T cells in GBM (Lines 331-338).

We respectfully submit that this work is well suited for a broad readership because it advances a generalizable therapeutic concept: overcoming solid tumor immunosuppression by simultaneously debulking malignant cells and removing suppressive myeloid populations. The GBM context is particularly compelling because myeloid dominance is a defining feature of the disease and a key reason why many immunotherapies have failed to achieve durable benefit[1,7]. In this setting, demonstration that a single CAR-T can mediate bicompartmental targeting with durable efficacy provides translational value beyond GBM.

Referee #3

"The authors have substantially improved the manuscript.

One note -- some of the responses to reviewers did not result in any changes to the manuscript -- given that reviewers do this in an effort to improve the manuscript, it would be nice if the authors incorporated more of the suggestions even if it didn't result in a new figure or experiment. Just one example -- I asked about whether there was a GPNMB KO mice, and in the context of its phenotype as it could relate to safety -- the authors kindly replied that there was and that there are references to the knockout mice in the rebuttal, but these references are not included or commented on in the manuscript."

Response: We thank the Reviewer for their reassessment and for noting the manuscript has been substantially improved. We also appreciate the Reviewer's previous guidance on the study. As requested, we have ensured all suggestions are incorporated into the manuscript. We have included information on $GPNMB^{KO}$ mice (Lines 301-306) and the role of GPNMB in mesenchymal-like GBM cells (Lines 318-326) in the discussion, based on our previous response to reviewers.

References

1 Begley, S. L., O'Rourke, D. M. & Binder, Z. A. CAR T cell therapy for glioblastoma: A review of the first decade of clinical trials. *Mol Ther* **33**, 2454–2461 (2025). https://doi.org/10.1016/j.ymthe.2025.03.004

2 Mateus-Tique, J. *et al.* Armored macrophage-targeted CAR-T cells reset and reprogram the tumor microenvironment and control metastatic cancer growth. *Cancer Cell* (2026). https://doi.org/10.1016/j.ccell.2025.12.021

3 Yagel, G. *et al.* Tumor-antigen-independent targeting of solid tumors by armored macrophage-directed anti-TREM2 CAR T cells. *Cancer Cell* (2025). https://doi.org/10.1016/j.ccell.2025.11.009

4 Rodriguez-Garcia, A. *et al.* CAR-T cell-mediated depletion of immunosuppressive tumor-associated macrophages promotes endogenous antitumor immunity and augments adoptive immunotherapy. *Nat Commun* **12**, 877 (2021). https://doi.org/10.1038/s41467-021-20893-2

5 Zhong, J. *et al.* Distinct roles of TREM2 in central nervous system cancers and peripheral cancers. *Cancer Cell* (2024). https://doi.org/10.1016/j.ccell.2024.05.001

6 Katzenelenbogen, Y. *et al.* Coupled scRNA-Seq and Intracellular Protein Activity Reveal an Immunosuppressive Role of TREM2 in Cancer. *Cell* **182**, 872–885 e819 (2020). https://doi.org/10.1016/j.cell.2020.06.032

7 Miller, T. E. *et al.* Programs, origins and immunomodulatory functions of myeloid cells in glioma. *Nature* (2025). https://doi.org/10.1038/s41586-025-08633-8
